# Effects of volenrelaxin in worsening heart failure with preserved ejection fraction: a phase 2 randomized trial

**DOI:** 10.1038/s41591-025-03939-6

**Published:** 2025-08-31

**Authors:** Barry A. Borlaug, Jeffrey M. Testani, Mark C. Petrie, Zhenzhong Wang, Jonathan Cunningham, Kirkwood F. Adams, Offer Amir, Jan Bělohlávek, Edimar Bocchi, Aguinaldo Freitas, Miguel Hominal, Toshiaki Kadokami, Bela Merkely, Christopher A. Miller, Julio Nuñez, Subodh Verma, Mehmet Birhan Yilmaz, Ena Oru, Flora Sam

**Affiliations:** 1 Department of Cardiovascular Diseases, Mayo Clinic, Rochester, MN USA; 2 Department of Internal Medicine, Section of Cardiovascular Medicine, Yale University School of Medicine, New Haven, CT USA; 3 School of Cardiovascular and Medical Sciences, British Heart Foundation Glasgow Cardiovascular Research Centre, University of Glasgow, Glasgow, UK; 4 Eli Lilly and Company, Indianapolis, IN USA; 5 Brigham and Women’s Hospital, Harvard Medical School, Boston, MA USA; 6 Departments of Medicine and Radiology, University of North Carolina, Chapel Hill, NC USA; 7 Heart Institute, Hadassah Medical Center, Jerusalem, Israel; 8 2nd Department of Internal Medicine, Cardiovascular Medicine, General University Hospital and 1st Faculty of Medicine, Charles University in Prague, Prague, Czechia; 9 Institute for Heart Diseases, Wroclaw Medical University, Wrocław, Poland; 10 Heart Failure Clinics, Instituto do Coração do Hospitasl das Clínicas da Faculdade de Medicina da USP, São Paulo, Brazil; 11 Universidade de São Paulo, São Paulo, Brazil; 12 Centro de Investigaciones Clínicas del Litoral, Santa Fe, Argentina; 13 Futsukaichi Hospital of Saiseikai Imperial Gift Foundation, Fukuoka, Japan; 14 Heart and Vascular Center, Semmelweis University, Budapest, Hungary; 15 Division of Cardiovascular Sciences, University of Manchester, Manchester Academic Health Science Centre, Manchester, UK; 16 BHF Manchester Centre for Heart and Lung Magnetic Resonance Research, Manchester University NHS Foundation Trust, Manchester, UK; 17 Department of Cardiology, Hospital Clínico Universitario de Valencia, Valencia, Spain; 18 INCLIVA and Department of Medicine, Universidad de Valencia, Valencia, Spain; 19 Division of Cardiac Surgery, St. Michael’s Hospital Unity Health Toronto, Toronto, Ontario Canada; 20 Department of Surgery, University of Toronto, Toronto, Ontario Canada; 21 Department of Cardiology, Faculty of Medicine, Dokuz Eylul University, Izmir, Turkey

**Keywords:** Outcomes research, Medical research

## Abstract

Relaxin is a peptide hormone that may decrease circulatory congestion and improve kidney function. In this study, we conducted a double-blind, international, multicenter trial to test whether volenrelaxin, a long-acting form of human relaxin, can improve left atrial (LA) function, reduce congestion and improve kidney function in patients with heart failure and preserved ejection fraction (HFpEF). We randomly assigned patients with New York Heart Association (NYHA) class II–IV HFpEF and recent heart failure (HF) decompensation to 25-mg, 50-mg or 100-mg volenrelaxin or placebo administered subcutaneously once weekly. The primary outcome was the change in LA reservoir strain at 26 weeks, with key secondary endpoints including changes in N-terminal pro-B-type natriuretic peptide (NT-proBNP), estimated glomerular filtration rate (eGFR) and safety. The trial was stopped early by the sponsor because of evidence for worsening congestion after 332 participants had been enrolled (mean age 74 years, 49% women, mean body mass index 30.6 kg m^−^^2^, 31.9% NYHA class III–IV). Compared to placebo, 25-mg volenrelaxin improved LA reservoir strain (+3.9%, 95% confidence interval (CI): 1.1–6.6, *P* = 0.006) but did not have effects on this outcome at 50-mg (+1.3%, 95% CI: −1.3 to 3.9, *P* = 0.332) or 100-mg (+0.9%, 95% CI: −1.8 to 3.6, *P* = 0.521) doses. At 26 weeks, volenrelaxin (pooling all dosages) increased NT-proBNP levels (+24.5%, 95% CI: 2.0–51.8) and had no significant effect on eGFR (+2.2 ml min^−1^ 1.73 m^−^^2^, 95% CI: −1.8 to 6.3). Volenrelaxin was also associated with a non-significant increase in risk for HF hospitalization compared to placebo (hazard ratio = 2.64, 95% CI: 0.93–7.56, *P* = 0.070), along with signals for an increased number of cardiovascular and renal serious adverse events (odds ratio = 2.52, 95% CI: 0.95–6.68, *P* = 0.056). In conclusion, despite some evidence for improvement in LA function at a low dose, treatment with this long-acting form of human relaxin was associated with worsening congestion in patients with recently decompensated HFpEF. ClinicalTrials.gov identifier: NCT05592275.

## Abstract (web summary)

In a multicenter, randomized trial of patients with heart failure with preserved ejection fraction and recent decompensation, treatment with volenrelaxin, a long-acting form of human relaxin, was associated with worsening congestion across multiple endpoints as compared to placebo.

## Main

Over half of all patients with HF have HFpEF^1,2^. In contrast to HF with reduced EF (HFrEF), there are fewer proven effective treatments for HFpEF, particularly those targeting patients with recently decompensated HFpEF, a group at very high risk who may respond differently than more stable, compensated patients. Individuals with current or prior decompensated HFpEF display more severe LA myopathy, greater burden of atrial fibrillation, more severe chronic kidney disease and often greater volume overload and sodium avidity than those without prior hospitalization, suggesting that therapies targeting these abnormalities may help reduce risk for subsequent events^3–5^.

Relaxin is a peptide hormone and cognate agonist at the relaxin family peptide receptor 1 (RXFP1) with purported vasodilatory, antiinflammatory and antifibrotic effects, which may decrease circulatory congestion and improve kidney function, and receptors are also expressed in atrial myocardium where activation may enhance atrial function^6–8^. Continuous intravenous infusion of short-acting recombinant human relaxin has demonstrated potentially beneficial acute effects in patients with acute HF, but previous trials were limited by the short-term (48-hour) exposure, which may explain why these acute beneficial effects did not translate into improvement in longer-term outcomes^9–14^. The effects of longer-term relaxin therapy in HF remain unknown. Volenrelaxin is a long-acting form of human relaxin modified to bind to serum albumin, extending its half-life to enable once-weekly dosing, allowing for longer-term RXFP1 activation^15^. In the present study, we performed a multicenter, randomized, placebo-controlled trial to determine whether treatment with volenrelaxin for 26 weeks would improve LA function, reduce congestion and improve kidney function in patients with recent worsening HFpEF.

## Results

**Fig. 1:**
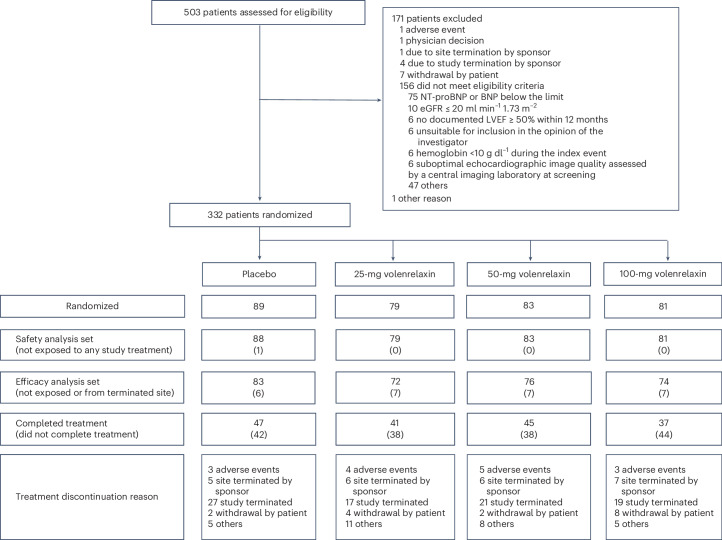
Participant flow diagram. Final disposition of participants from eligibility assessment to study completion is depicted, along with safety and efficacy analysis sets.

Between 3 February 2023 and 22 January 2025, 503 patients were screened and 332 patients were enrolled and randomly assigned to volenrelaxin 25 mg (*n* = 79), volenrelaxin 50 mg (*n* = 83), volenrelaxin 100 mg (*n* = 81) or placebo (*n* = 89) at 64 centers in 10 countries (Fig. 1). The study was stopped early by the sponsor after the first interim analysis because a low probability of observing benefit with continued enrollment was interpreted. Although there was not evidence of increased HF events in the volenrelaxin group, multiple signals for worsening congestion were perceived, which motivated the decision to terminate the study prematurely. A total of 170 patients completed the full 26-week protocol.

The baseline characteristics of the patient groups were similar and typical of individuals with worsening HFpEF (Table 1). Mean age was 74 years; 49% were women; mean body mass index (BMI) was 30.6 kg m^−^^2^; over half of the participants (50.6%) were in atrial fibrillation or flutter; and 31.9% of participants were determined to be in NYHA class III–IV at enrollment. At baseline, participants had severely impaired LA reservoir strain (17.8%, normal > 39%), elevated NT-proBNP (1,299, interquartile range (IQR) 711–2,348 pg ml^−1^), myocardial injury (troponin T 24, IQR 18–38 pg ml^−1^), a reduced eGFR (52 ± 21 ml min^−1^ 1.73 m^−^^2^) and high prevalence of albuminuria (43.7% of participants). Background medical therapy consisted of loop diuretics in 92.8%, sodium glucose co-transporter 2 (SGLT2) inhibitors in 40.4% and mineralocorticoid receptor antagonists (MRAs) in 43.4% (Table 1).

**Table 1:** Baseline characteristics

	Placebo (*n* = 89)	Volenrelaxin 25 mg (*n* = 79)	Volenrelaxin 50 mg (*n* = 83)	Volenrelaxin 100 mg (*n* = 81)	Total (*n* = 332)
Age (years)	74.6 (9.1)	74.1 (10.1)	74.7 (8.1)	72.4 (9.7)	74.0 (9.3)
Women, *n* (%)	29 (32.6)	46 (58.2)	45 (54.2)	44 (54.3)	164 (49.4)
Race					
Asian	7 (7.9)	3 (3.8)	4 (4.9)	3 (3.8)	17 (5.2)
Black or African American	3 (3.4)	4 (5.1)	3 (3.7)	8 (10.0)	18 (5.5)
White	79 (88.8)	72 (91.1)	74 (91.4)	68 (85.0)	293 (89.1)
Other or mixed race	0 (0.0)	0 (0.0)	0 (0.0)	1 (1.2)	1 (0.3)
Ethnicity					
Hispanic or Latino	37 (41.6)	41 (51.9)	39 (47.0)	34 (42.0)	151 (45.5)
Not Hispanic or Latino	52 (58.4)	37 (46.8)	44 (53.0)	45 (55.6)	178 (53.6)
Region					
North America	6 (6.7)	5 (6.3)	8 (9.6)	8 (9.9)	27 (8.1)
Europe	44 (49.4)	39 (49.4)	41 (49.4)	40 (49.4)	164 (49.4)
Latin America	32 (36.0)	33 (41.8)	32 (38.6)	30 (37.0)	127 (38.3)
Asia	7 (7.9)	2 (2.5)	2 (2.4)	3 (3.7)	14 (4.2)
BMI (kg m^−^^2^)	28.7 (6.3)	30.5 (5.9)	31.4 (6.0)	32.0 (8.0)	30.6 (6.7)
HF severity and phenotyping					
NYHA class II	58 (65.2)	55 (69.6)	61 (73.5)	51 (63.0)	225 (67.8)
NYHA class III–IV	31 (34.8)	24 (30.4)	22 (26.5)	29 (35.8)	106 (31.9)
NT-proBNP, median (IQR), pg ml^−1^	1,374 (711, 2,664)	1,158 (558, 2,011)	1,391 (873, 2,614)	1,239 (595, 2,218)	1,299 (711, 2,348)
Estimated plasma volume (ml)	2,648 (640)	2,627 (477)	2,732 (560)	2,795 (691)	2,699 (598)
Systolic blood pressure (mmHg)	123 (15)	126 (16)	126 (15)	126 (16)	125 (15)
KCCQ-TSS (points)	61.1 (23.4)	53.1 (22.4)	55.7 (19.8)	52.9 (23.9)	55.8 (22.6)
KCCQ-CSS (points)	56.0 (22.0)	49.0 (20.8)	52.2 (20.7)	50.9 (21.9)	52.1 (21.4)
KCCQ-OSS (points)	56.1 (19.5)	52.5 (19.8)	52.8 (20.8)	53.2 (21.2)	53.7 (20.3)
Troponin T, median (IQR), ng l^−1^	26 (18, 40)	23 (17, 35)	23 (18, 37)	23 (18, 38)	24 (18, 38)
Atrial fibrillation/flutter, *n* (%)	48 (53.9)	37 (46.8)	43 (51.8)	40 (49.4)	168 (50.6)
Hypertension, *n* (%)	83 (93.3)	71 (89.9)	73 (88.0)	71 (87.7)	298 (89.8)
Diabetes mellitus, *n* (%)	35 (39.3)	34 (43.0)	37 (44.6)	37 (45.7)	143 (43.1)
Coronary artery disease	31 (34.8)	22 (27.8)	21 (25.3)	31 (38.3)	105 (31.6)
Medications					
Diuretics	82 (92.1)	74 (93.7)	78 (94.0)	76 (93.8)	310 (93.4)
ACEi, ARB or ARNi	58 (65.2)	62 (78.5)	62 (74.7)	60 (74.1)	242 (72.9)
Beta-blocker	72 (80.9)	61 (77.2)	69 (83.1)	59 (72.8)	261 (78.6)
MRA	41 (46.1)	36 (45.6)	33 (39.8)	34 (42.0)	144 (43.4)
SGLT2 inhibitor	34 (38.2)	31 (39.2)	37 (44.6)	32 (39.5)	134 (40.4)
Cardiac structure and function					
LV ejection fraction, %	55.2 (9.6)	59.8 (9.3)	57.5 (9.1)	58.7 (10.5)	57.7 (9.7)
LV mass index, g m^−^^2^	114.5 (32.9)	108.9 (36.2)	106.7 (31.9)	107.4 (36.1)	109.4 (34.3)
LV global longitudinal strain, %	−13.5 (3.3)	−14.4 (3.9)	−13.9 (4.0)	−14.0 (3.8)	−13.9 (3.7)
LA maximal volume index, ml m^−^^2^	45.3 (18.6)	41.5 (19.8)	42.9 (15.4)	41.7 (15.6)	42.9 (17.4)
LA minimal volume index, ml m^−^^2^	31.3 (17.4)	29.0 (19.4)	29.7 (13.6)	28.4 (14.9)	29.7 (16.4)
LA reservoir strain, %	17.8 (7.3)	19.0 (9.4)	17.1 (6.9)	17.5 (7.7)	17.8 (7.8)
E/e′ ratio	12.7 (4.9)	12.8 (6.6)	13.0 (4.3)	13.8 (5.7)	13.1 (5.4)
Transmitral E/A ratio	2.1 (1.4)	2.0 (1.5)	2.1 (1.6)	2.2 (1.7)	2.1 (1.5)
Cardiac output (ml min^−1^)	4,343 (1,585)	4,217 (1,762)	4,101 (1,388)	4,317 (1,410)	4,252 (1,529)
Total peripheral resistance (WU)	23.7 (8.6)	24.9 (9.1)	25.1 (8.2)	23.4 (8.5)	24.2 (8.6)
Kidney function					
eGFR (ml min^−1^ 1.73 m^−^^2^)	52.4 (21.0)	52.5 (22.4)	50.8 (19.4)	52.4 (21.4)	52.0 (21.0)
Serum creatinine (mg dl^−1^)	1.2 (0.9, 1.5)	1.1 (0.8, 1.5)	1.1 (0.9, 1.5)	1.1 (0.8, 1.5)	1.1 (0.9, 1.5)
Cystatin C (mg l^−1^)	1.5 (1.3, 2.0)	1.6 (1.2, 2.0)	1.6 (1.3, 1.8)	1.6 (1.2, 2.0)	1.6 (1.2, 1.9)
UACR (mg g^−1^)	20.5 (9.3, 57.5)	24.5 (10.5, 102.0)	20.5 (12.0, 76.0)	26 (7.5, 62.0)	23 (9.5, 70.0)
Microalbuminuria, *n* (%)	32 (40.0)	25 (32.9)	25 (32.1)	25 (33.3)	107 (34.6)
Macroalbuminuria, *n* (%)	4 (5.0)	7 (9.2)	7 (9.0)	10 (13.3)	28 (9.1)

Values show number (percentage), mean (s.d.) or median (25th, 75th percentile).

ACEi, angiotensin converting enzyme inhibitor; ARB, angiotensin receptor blocker; ARNi, angiotensin receptor/neprilysin inhibitor.

### Primary outcome

The primary outcome of change in LA reservoir strain at 26 weeks was improved compared to placebo with volenrelaxin 25 mg (+3.9% (95% CI: 1.1–6.6), *P* = 0.006), with no significant effect noted with volenrelaxin 50 mg (+1.3% (95% CI: −1.3 to 3.9), *P* = 0.332) or volenrelaxin 100 mg (+0.9% (95% CI: −1.3 to 3.6), *P* = 0.521) or the pooled 50-mg and 100-mg dosages (Table 2). No significant effect was observed on LA reservoir strain compared to placebo pooling all doses of volenrelaxin (+2.0% (95% CI: −0.2 to 4.2), *P* = 0.075). Sex disaggregated findings are presented in Extended Data Tables 1 and 2.

**Table 2:** Effects of volenrelaxin on primary and secondary endpoints

	Placebo	Volenrelaxin	Treatment difference (95% CI)	*P* value
Primary endpoint				
Change in LA reservoir strain at 26 weeks, %	*n* = 38			
25-mg volenrelaxin (*n* = 32)	−1.9	1.9	+3.9 (1.1–6.6)	0.006
50-mg volenrelaxin (*n* = 37)	−1.9	−0.6	+1.3 (−1.3 to 3.9)	0.332
100-mg volenrelaxin (*n* = 31)	−1.9	−1.0	+0.9 (−1.8 to 3.6)	0.521
Pooled 50/100-mg volenrelaxin (*n* = 68)	−2.0	0.8	+1.1 (−1.2 to 3.4)	0.329
Secondary endpoints				
Change in NT-proBNP at 12 weeks, %	*n* = 58			
25-mg volenrelaxin (*n* = 50)	−10.6	+17.3	+31.3 (5.4–63.5)	0.015
50-mg volenrelaxin (*n* = 55)	−10.6	−8.8	+2.1 (−17.8 to 26.8)	0.851
100-mg volenrelaxin (*n* = 47)	−10.6	+1.6	+13.7 (−8.9 to 42.0)	0.256
Pooled 50/100-mg volenrelaxin (*n* = 102)	−10.7	−3.6	+8.0 (−10.5 to 30.3)	0.423
Change in NT-proBNP at 26 weeks, %	*n* = 44			
25-mg volenrelaxin (*n* = 39)	−22.9	+9.9	+42.6 (11.3–82.7)	0.005
50-mg volenrelaxin (*n* = 43)	−22.9	−18.7	+5.5 (−17.3 to 34.4)	0.667
100-mg volenrelaxin (*n* = 36)	−22.9	+1.0	+31.1 (1.9–68.5)	0.035
Pooled 50/100-mg volenrelaxin (*n* = 79)	−23.0	−9.7	+17.2 (−5.2 to 44.8)	0.142
Change in eGFR at 12 weeks, ml min^−1^ 1.73 m^−2^	*n* = 60			
25-mg volenrelaxin (*n* = 51)	+0.4	+5.5	+5.1 (1.3–8.8)	0.009
50-mg volenrelaxin (*n* = 54)	+0.4	+6.3	+5.9 (2.2–9.6)	0.002
100-mg volenrelaxin (*n* = 47)	+0.4	+3.5	+3.0 (−0.8 to 6.8)	0.116
Pooled 50/100-mg volenrelaxin (*n* = 101)	+0.4	+5.0	+4.5 (1.3–7.7)	0.007
Change in eGFR at 26 weeks, ml min^−1^ 1.73 m^−2^	*n* = 45			
25-mg volenrelaxin (*n* = 38)	+1.8	+4.8	+3.1 (−2.0 to 8.1)	0.236
50-mg volenrelaxin (*n* = 44)	+1.8	+4.3	+2.5 (−2.5 to 7.4)	0.322
100-mg volenrelaxin (*n* = 37)	+1.8	+3.0	+1.3 (−3.9 to 6.4)	0.627
Pooled 50/100-mg volenrelaxin (*n* = 81)	+1.8	+3.7	+1.9 (−2.4 to 6.2)	0.390
Change in LA reservoir strain at 12 weeks, %	*n* = 45			
25-mg volenrelaxin (*n* = 40)	−1.0	+0.3	+1.2 (−1.9 to 4.3)	0.440
50-mg volenrelaxin (*n* = 44)	−1.0	−0.1	+0.8 (−2.2 to 3.8)	0.591
100-mg volenrelaxin (*n* = 41)	−1.0	+1.3	+2.3 (−0.8 to 5.3)	0.145
Pooled 50/100-mg volenrelaxin (*n* = 85)	−1.0	+0.5	+1.5 (−1.1 to 4.1)	0.250
Change in LA maximal volume index at 12 weeks, ml m^−2^	*n* = 47			
25-mg volenrelaxin (*n* = 41)	+2.7	+4.9	+2.2 (−1.7 to 6.2)	0.268
50-mg volenrelaxin (*n* = 45)	+2.7	+3.7	+1.0 (−2.9 to 4.9)	0.610
100-mg volenrelaxin (*n* = 43)	+2.7	+3.6	+1.0 (−3.0 to 4.9)	0.624
Pooled 50/100-mg volenrelaxin (*n* = 88)	+2.7	+3.8	+1.1 (−2.3 to 4.4)	0.534
Change in LA maximal volume index at 26 weeks, ml m^−2^	*n* = 38			
25-mg volenrelaxin (*n* = 33)	+1.0	+2.9	+2.0 (−2.7 to 6.6)	0.403
50-mg volenrelaxin (*n* = 38)	+1.0	+6.4	+5.4 (0.9–9.9)	0.019
100-mg volenrelaxin (*n* = 32)	+1.0	+2.5	+1.5 (−3.1 to 6.1)	0.525
Pooled 50/100-mg volenrelaxin (*n* = 70)	+1.0	+4.7	+3.7 (−0.3 to 7.6)	0.069

Treatment comparison is assessed using two-sided *t*-test based on least square means from MMRM. No adjustment for multiplicity was performed.

For NT-proBNP, log transformation is applied first on the data, and then the estimation of change from baseline in log scale is transformed back into percentage from baseline in original scale.

Abbreviations are as in Table 1.

### Secondary outcomes

**Fig. 2:**
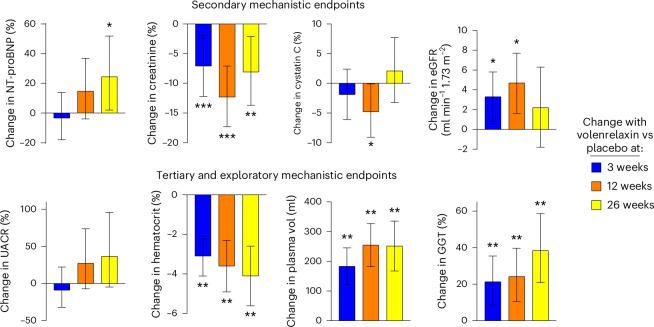
Effects of volenrelaxin as compared to placebo on measures of congestion and kidney function. The graphs show effects of volenrelaxin versus placebo on secondary endpoints (NT-proBNP, creatinine, cystatin C and eGFR) and tertiary and exploratory endpoints (UACR, hematocrit, estimated plasma volume and GGT) at 3 weeks, 12 weeks and 26 weeks. Boxes indicate the least square mean change difference between groups, with error bars showing the 95% CI. Sample sizes for comparisons of the change from baseline between groups for NT-proBNP at 3 weeks *n* = 189 volenrelaxin-treated/66 placebo-treated, at 12 weeks *n* = 152/58 and at 26 weeks *n* = 118/44; for estimated plasma volume change, *n* = 189/62, *n* = 166/60, *n* = 126/39; for hematocrit change, *n* = 190/62, *n* = 166/61, *n* = 126/39; for GGT change, *n* = 213/79, *n* = 190/69, *n* = 136/45; for UACR change, *n* = 172/62, *n* = 143/56, *n* = 106/38; for creatinine change, *n* = 188/74, *n* = 153/62, *n* = 120/45; for cystatin C change, *n* = 190/73, *n* = 154/60, *n* = 119/45; for eGFR change, *n* = 185/72, *n* = 152/60, *n* = 119/45. Treatment comparisons are assessed using a two-sided *t*-test based on least square means from MMRM. *P* values of each parameter (week 3/week 12/week 26) are as follows: NT-proBNP (0.6827/0.1274/0.0310); creatinine (0.0069/<0.0001/0.0092); cystatin C (0.3798/0.0435/0.4456); eGFR (0.0105/0.0027/0.2751); UACR (0.5364/0.1313/0.0886); hematocrit (<0.0001/<0.0001/<0.0001); plasma volume (<0.0001/<0.0001/<0.0001); GGT (0.0006/0.0003/<0.0001). No adjustment for multiplicity was performed. *P < 0.05 for volenrelaxin group (pooled dosages) versus placebo; **P < 0.01 for volenrelaxin group (pooled dosages) versus placebo; ***P < 0.001 for volenrelaxin group (pooled dosages) versus placebo. vol, volume.

Compared to placebo, volenrelaxin increased or tended to increase plasma NT-proBNP levels at 12 weeks (+31.3%, +2.1% and +13.7% for 25 mg, 50 mg and 100 mg, respectively; Table 2), with greater increases at 26 weeks (+42.6%, +5.5% and +31.1%). Pooling all doses, volenrelaxin increased NT-proBNP at 26 weeks compared to placebo (+24.5% (95% CI: 2.0–51.8%), *P* = 0.031; Table 3 and Fig. 2). At 26 weeks, volenrelaxin increased or tended to increase LA maximal volume (+2.0, +5.4 and +1.5 ml m^−^^2^ for 25 mg, 50 mg and 100 mg, respectively; Table 2) and LA minimal volume (+1.1, +4.4 and +1.8 ml m^−^^2^ for 25 mg, 50 mg and 100 mg, respectively; Extended Data Table 3) as compared to placebo.

**Table 3:** Physiologic effects of volenrelaxin (pooled dosages) on congestion, hemodynamics and renal function

	3-week LS mean change difference (95% CI)	12-week LS mean change difference (95% CI)	26-week LS mean change difference (95% CI)
Measures of circulatory congestion			
NT-proBNP (% change) (*n* = 189/66, *n* = 152/58, *n* = 118/44)	−3.4 (−18.0 to 13.9)	+14.7 (−3.9 to 36.9)	+24.5 (2.0–51.8) *
E/e′ ratio (–, *n* = 100/40, *n* = 84/34)	–	+0.5 (−1.1 to 2.0)	+1.7 (0.2–3.2) *
Hematocrit (%) (*n* = 190/62, *n* = 166/61, *n* = 126/39)	−3.1 (−4.1 to −2.2) ***	−3.6 (−4.9 to −2.3) ***	−4.1 (−5.6 to −2.6) ***
Hemoglobin (mg dl^−1^) (*n* = 190/62, *n* = 166/61, *n* = 126/39)	−0.8 (−1.1 to −0.6) ***	−1.0 (−1.4 to −0.6) ***	−1.2 (−1.7 to −0.8) ***
Serum sodium (mEq l^−1^) (*n* = 212/79, *n* = 189/69, *n* = 136/45)	−1.2 (−1.8 to −0.5) **	−0.4 (−1.1 to 0.4)	−1.3 (−2.3 to −0.3) *
Estimated plasma volume (ml) (*n* = 189/62, *n* = 166/60, *n* = 126/39)	+182 (125–238) ***	+225 (160–291) ***	+220 (140–300) ***
GGT (% change) (*n* = 213/79, *n* = 190/69, *n* = 136/45)	+21.3 (8.7–35.4) **	+24.2 (10.5–39.6) **	+38.4 (20.8–58.6) ***
Measures of hemodynamics			
Systolic blood pressure (mmHg) (*n* = 222/81, *n* = 195/69, *n* = 140/45)	−3.1 (−6.6 to 0.3)	−2.9 (−6.6 to 0.8)	0.1 (−4.2 to 4.3)
Mean blood pressure (mmHg) (*n* = 222/81, *n* = 195/69, *n* = 139/45)	−2.5 (−4.8 to −0.2) *	−2.1 (−4.6 to 0.4)	−1.5 (−4.4 to 1.3)
Cardiac output (ml min^−1^) (–, *n* = 82/35, *n* = 66/30)	–	+561 (72–1,050) *	+575 (73–1,076) *
Total peripheral resistance (WU) (–, *n* = 82/34, *n* = 65/30)	–	−4.3 (−7.0 to -1.6) **	−4.4 (−7.4 to −1.4) **
Measures of renal function			
eGFR (ml min^−1^ 1.73 m^−2^) (*n* = 185/72, *n* = 152/60, *n* = 119/45)	+3.3 (0.8–5.8) *	+4.7 (1.6–7.7) **	+2.2 (−1.8 to 6.3)
Creatinine (% change) (*n* = 188/74, *n* = 153/62, *n* = 120/45)	−7.1 (−12.0 to −2.0) ***	−12.3 (−17.3 to −7.1) ***	−8.1 (−13.7 to −2.1) **
Cystatin C (% change) (*n* = 190/73, *n* = 154/60, *n* = 119/45)	−1.9 (−6.1 to 2.4)	−4.8 (−9.1 to −0.1) *	2.1 (−3.2 to 7.7)
UACR (% change) (*n* = 172/62, *n* = 143/56, *n* = 106/38)	−8.9 (−32.1 to 22.3)	+27.2 (−7.0 to 73.9)	+36.7 (−4.6 to 95.9)
UACR (% change) (with baseline > 30 mg g^−1^) (*n* = 78/26, *n* = 64/24, *n* = 44/16)	−15.9 (−46.5 to 32.2)	+53.0 (−5.2 to 146.8)	+52.9 (−13.3 to 169.6)
Troponin T (% change) (*n* = 181/63, *n* = 139/55, *n* = 110/41)	−0.5 (−10.1 to 10.1)	0.5 (−10.0 to 12.1)	4.9 (−7.0 to 18.2)

Treatment comparison was assessed using two-sided *t*-test based on least square means from MMRM. No adjustment for multiplicity was performed.

For NT-proBNP, log transformation is applied first on the data, and then the estimation of change from baseline in log scale (LS) is transformed back into percentage from baseline in original scale.

*P < 0.05 for change with volenrelaxin versus change with placebo; **P < 0.01 for change with volenrelaxin versus change with placebo; ***P < 0.001 for change with volenrelaxin versus change with placebo.

Abbreviations are as in Table 1.

Compared to placebo, volenrelaxin increased eGFR at 12 weeks (+5.1, +5.9, +3.0 ml min^−1^ 1.73 m^−^^2^ for 25 mg, 50 mg and 100 mg, respectively; Table 2 and Fig. 2), with no significant effect at 26 weeks (+3.1, +2.5, +1.3 ml min^−1^ 1.73 m^−2^) and no effect pooling all volenrelaxin doses at 26 weeks (+2.2 (95% CI: −1.8 to 6.3) ml min^−1^ 1.73 m^−2^, *P* = 0.275; Table 3). Serum creatinine was reduced with volenrelaxin versus placebo at 12 weeks (−16.3%, −12.0%, −8.3%; Extended Data Table 3), with trends to reduction at 26 weeks (−11.1%, −7.3%, −5.7%) and a significant reduction pooling all doses (−8.1% (95% CI: −13.7% to −2.1%), *P* = 0.009; Table 3). Volenrelaxin had a trend of reducing cystatin C versus placebo at 12 weeks (−5.5%, −5.2%, −3.3%; Extended Data Table 3), with no effect at 26 weeks (2.2%, 2.3%, 1.8%) and no significant effect reduction pooling all doses (2.1% (95% CI: −3.2% to 7.7%), *P* = 0.446).

### Clinical events and safety

**Extended Data Fig. 1:**
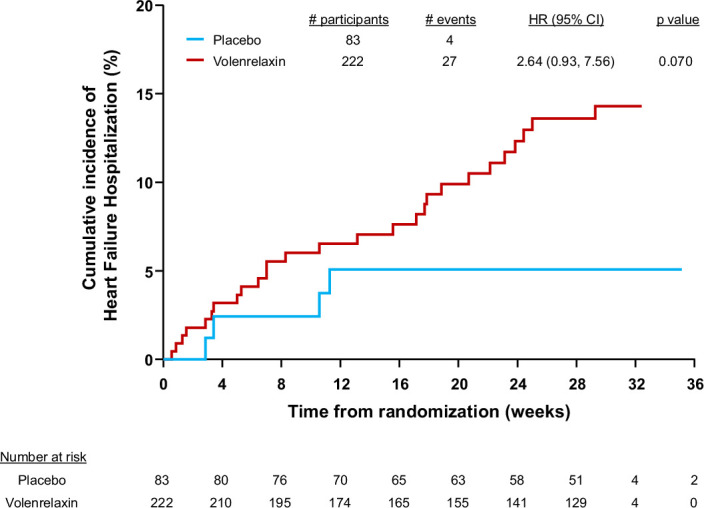
Effects of volenrelaxin on heart failure hospitalization. The graph shows the results of an exploratory analysis comparing the cumulative incidence of hospitalization for heart failure (HF) in patients treated with volenrelaxin (pooling all dosages) and placebo. Numbers of participants at risk, events, and hazard ratio (HR) for HF hospitalization are depicted for placebo (blue) and pooled volenrelaxin dosages (red). Treatment comparison is assessed using Cox-proportional hazard model. CI, confidence interval.

An adjudicated HF hospitalization occurred in four patients (4.8%) in the placebo group and in 27 patients (12.2%) in the pooled volenrelaxin group—9.9 and 25.3 events per 100 patient-years of follow-up, respectively (hazard ratio (HR) = 2.64; 95% CI: 0.93–7.56, *P* = 0.070; Table 4 and Extended Data Fig. 1). Cardiovascular death, HF hospitalization or urgent HF visit occurred in 12 patients (14.5%) in the placebo group and in 43 patients (19.4%) in the pooled volenrelaxin group—29.6 and 40.4 events per 100 patient-years of follow-up, respectively (HR = 1.40, 95% CI: 0.74–2.65, *P* = 0.308; Table 4).

**Table 4:** Clinical events and safety

	Placebo		Volenrelaxin (pooled dosages)			
	*n* (%)	Rate per 100 PYs	*n* (%)	Rate per 100 PYs	HR or OR (95% CI)	*P* value
Adjudicated clinical events						
HF hospitalization	4 (4.8%)	9.9	27 (12.2%)	25.3	2.64 (0.93–7.56)	0.070
Urgent HF visit	7 (8.4%)	17.3	19 (8.6%)	17.8	1.04 (0.44–2.47)	0.935
CV death	2 (2.4%)	4.9	6 (2.7%)	5.6	1.12 (0.23–5.54)	0.891
HF hospitalization, urgent HF visit or CV death	12 (14.5%)	29.6	43 (19.4%)	40.4	1.40 (0.74–2.65)	0.308
Adverse events						
SAE	15 (17.0%)	–	65 (26.8%)	–	1.78 (0.95–3.32)	0.068
Death	2 (2.3%)	–	8 (3.3%)	–	1.46 (0.31–7.03)	0.632
Cardiac SAE	5 (5.7%)	–	30 (12.4%)	–	2.34 (0.88–6.23)	0.082
Renal SAE	0 (0%)	–	4 (1.7%)	–	–	–
Cardiac or renal SAE	5 (5.7%)		32 (13.2%)	–	2.52 (0.95–6.68)	0.056
Any TEAE	58 (65.9%)	–	158 (65.0%)	–	0.96 (0.58–1.61)	0.881
Cardiac disorders	14 (15.9%)	–	59 (24.3%)	–	1.69 (0.89–3.22)	0.105
Renal disorders	4 (4.6%)	–	27 (11.1%)	–	2.63 (0.89–7.73)	0.070
Cardiac or renal TEAE	18 (20.5%)	–	76 (31.3%)	–	1.77 (0.99–3.18)	0.054
Hypotension	6 (6.8%)	–	13 (5.4%)	–	0.77 (0.28–2.10)	0.612

For outcome events, treatment comparison is assessed using Cox proportional hazard model. Treatment comparison is assessed using the log-rank test. For other clinical events and adverse events, treatment comparison is assessed using OR by Fisher’s exact test or chi-square test, as appropriate.

CV, cardiovascular; PY, patient-year; SAE, serious adverse event; TEAE, treatment-emergent adverse event. Other abbreviations are as in Table 1.

No effect of volenrelaxin was observed compared to placebo on NYHA class at 12 weeks, but, at 26 weeks, NYHA class improvement was noted in 39 patients (28%) in the pooled volenrelaxin group and in 23 patients (49%) in the placebo group (odds ratio (OR) for improvement = 0.40 (95% CI: 0.20–0.80), *P* = 0.008; Extended Data Table 4). Volenrelaxin worsened Kansas City Cardiomyopathy Questionnaire total symptom scores (KCCQ-TSSs), KCCQ clinical summary scores (KCCQ-CSSs) and KCCQ overall summary scores (KCCQ-OSSs) at 12 weeks, with no significant effect at 26 weeks (Extended Data Table 4). Intensification of loop diuretics was observed in 58 patients (26%) in the pooled volenrelaxin group and in 15 patients (18%) in the placebo group (OR = 1.60 (95% CI: 0.85–3.02), *P* = 0.142).

Adverse events were common in both groups but numerically higher in volenrelaxin-treated patients (Table 4 and Extended Data Table 5). The likelihood of cardiovascular or renal serious adverse events tended to be higher in those treated with volenrelaxin compared to placebo (OR = 2.52, 95% CI: 0.95–6.68, *P* = 0.056). Adverse events leading to discontinuation occurred in 15 patients (4.5%) in total: three (3.3%) in the placebo group and 12 (4.9%) in the pooled volenrelaxin group.

### Exploratory analysis of physiologic effects of volenrelaxin

**Extended Data Fig. 2:**
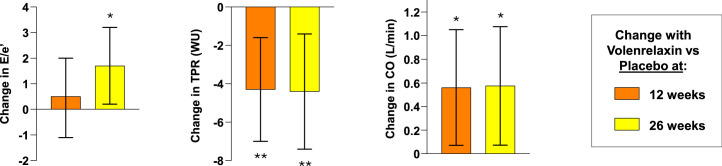
Effects of volenrelaxin on hemodynamics. The graphs show effects of volenrelaxin as compared with placebo on measures of left atrial filling pressures (E/e’ ratio), arterial afterload (total peripheral resistance, TPR) and cardiac output (CO) at 12, and 26 weeks. E/e’ and CO were prespecified tertiary endpoints and TPR was a post hoc exploratory endpoint. Cardiac output was measured using pulse wave Doppler of the left ventricular outflow tract and TPR was calculated as the quotient of mean arterial pressure divided by CO. Boxes indicate the least square mean change difference between groups, with error bars showing the 95% confidence interval. For E/e’ change n = 100/40, n = 84/30; for TPR change n = 82/34, n = 65/30; for CO change n = 82/35, n = 66/30. Treatment comparisons are assessed using a 2-sided t-test based on least square means from mixed model repeated measures model. P value of each parameter (week 12 / week 26): E/e’ (0.5319/0.0315); TPR (0.0024/0.0046), CO (0.0250/0.0251). No adjustment for multiplicity was performed. * p < 0.05 for volenrelaxin group (pooled dosages) vs placebo. ** p < 0.01 for volenrelaxin group (pooled dosages) vs placebo.

To provide greater context and explore the physiologic effects of volenrelaxin treatment over time, we analyzed serial changes in measures of cardiac function, hemodynamics and kidney function from assessments performed over the course of the trial (Table 3, Fig. 2 and Extended Data Fig. 2). Compared to placebo, volenrelaxin increased estimated plasma volume and E/e′ (a surrogate for LA pressure) while decreasing hematocrit, hemoglobin and serum sodium. There was no evidence of volenrelaxin effect on NT-proBNP at 3 weeks but progressively greater increases to 12 weeks, which became statistically significant by 26 weeks. Serum gamma-glutamyl transferase (GGT) levels (a biomarker that is elevated with liver congestion) were higher in patients treated with volenrelaxin versus placebo at all timepoints.

A significant increase in cardiac output was observed at 12 weeks and 26 weeks with volenrelaxin compared to placebo, along with a reduction in total peripheral resistance, translating into no significant effect of volenrelaxin on systolic blood pressure (Table 3 and Extended Data Fig. 2). No significant effect of volenrelaxin was observed on urine albumin-to-creatinine ratio (UACR) or myocardial injury (troponin T; Fig. 2 and Table 3).

## Discussion

This study was prospectively designed to evaluate the effects of a long-acting form of human relaxin on cardiac function, circulatory congestion and kidney function in patients with recent worsening HFpEF, a group at very high risk for recurrent events. The trial was terminated before planned enrollment was completed, limiting power and, therefore, certainty about the conclusions, but, as compared to placebo, low-dose volenrelaxin improved the primary endpoint of LA reservoir strain, with no significant effect at higher dosages. Kidney function was improved with volenrelaxin with shorter duration of treatment, with no significant effect observed at 26 weeks. Despite these favorable signals, there was evidence of worsening congestion across multiple independent assessments, including higher NT-proBNP levels, estimated LA pressures (E/e′ ratio), cardiac output and estimated plasma volume and lower hematocrit, hemoglobin and serum sodium. This phase 2 trial was not powered to detect an effect of volenrelaxin on HF hospitalization or cardiovascular death, but there were signals for greater risk with volenrelaxin, particularly for HF hospitalization, which did not reach statistical significance. A large and rapid improvement in health status and congestion was observed in the placebo group, as expected in recently hospitalized patients who have been treated and are receiving frequent follow-up care, but this effect was muted in the volenrelaxin group, evidenced by lower likelihood of NYHA class improvement at 26 weeks, trends for a greater need for loop diuretic intensification and suggestive evidence of worsening symptom severity based upon changes in KCCQ scores. The evidence for worsening rather than improved congestion with volenrelaxin does not support further evaluation of this molecule as a treatment for patients with worsening HFpEF.

Relaxin is a peptide hormone synthesized by the placenta during pregnancy that has been shown in preclinical and clinical studies to have vasodilatory, antifibrotic and antiinflammatory effects, along with increases in renal plasma flow and potential improvements in eGFR, changes that help support the heightened circulatory and metabolic demands of pregnancy^6–8^. These effects could also be advantageous in patients with HF, forming the basis for clinical trials of short-term relaxin treatment in the setting of acute HF. In the RELAX-AHF trial, 48-hour treatment with intravenous relaxin reduced LA pressure and dyspnea in patients with acute HF but had no effect on hospital readmission^10,12^. In the larger RELAX-AHF-2 trial, the same 48-hour treatment with intravenous relaxin did not reduce the incidence of cardiovascular death at 180 days or worsening HF at 5 days compared to placebo^13^, but, in a large meta-analysis of all six phase 3 trials (*n* = 11,359), 48-hour infusion with relaxin was shown to reduce the risk of worsening HF to day 5 while also reducing serum creatinine, NT-proBNP and troponin levels^14^.

These favorable signals with short-term administration suggest that longer-duration treatment with relaxin could produce salutary effects in patients with HF, but previously tested recombinant human relaxin formulations had very short half-life, requiring continuous infusion, making such trials impractical^14^. Volenrelaxin is a long-acting human relaxin analogue and potent agonist of the RXFP1 receptor that consists of human relaxin fused to the serum albumin-binding VHH domain, allowing for longer half-life and once-weekly dosing without substantial modification of the parent relaxin molecule^15^. Patients with HFpEF, particularly HFpEF with recent worsening congestion, display severe LA myopathy, manifested by atrial fibrillation, LA dilatation and LA dysfunction, along with greater congestion and kidney dysfunction^3–5^. This led us to hypothesize that volenrelaxin would improve LA function and, given the salutary effects with short-term relaxin^9–14^, also reduce congestion and improve kidney function in patients with worsening HFpEF.

Contrary to this hypothesis, we found that congestion worsened with 26 weeks of volenrelaxin treatment. This was evidenced by increases in NT-proBNP, E/e′ ratio and estimated plasma volume (Fig. 2). Hematocrit and hemoglobin decreased, likely reflecting hemodilution from volume expansion. Red blood cell mass was not directly measured to verify this mechanism, but the observed reduction in serum sodium also supports expansion of plasma water. Cardiac output increased, likely related, at least in part, to the Frank–Starling mechanism accompanying volume expansion that accompanied vasodilation, and LA volumes also tended to increase, in keeping with greater congestion. GGT levels are known to increase in the setting of HF decompensation due to liver congestion^16^, and these were also increased in the volenrelaxin group as compared to placebo. Although isolated changes in one or two of these markers of congestion could be explained by the play of chance, the totality and consistency of evidence across multiple independent measures strongly supports the interpretation of an increase in congestion with volenrelaxin.

Biochemical and echocardiographic evidence of volume expansion with volenrelaxin was corroborated by suggestions of worsening clinical status, including lower likelihood of NYHA class improvement at 26 weeks with volenrelaxin (28% versus 49%), even as loop diuretic intensification tended to be greater compared to placebo, and signals of worsening KCCQ scores. No statistically significant differences were observed in the risk for HF hospitalization, but the directionality of relationships favored placebo (HR = 2.64, 95% CI: 0.93–7.56, *P* = 0.070). The numbers of cardiac and renal serious adverse events and treatment-emergent adverse events were also greater in the volenrelaxin group.

The worsening congestion observed with volenrelaxin may appear surprising considering earlier short-term studies with relaxin showing vasodilation, reduction in LA pressure and decreases rather than increases in NT-proBNP^9–14,17^. However, the present results with longer-term RXFP1 activation complement and are compatible with the acute studies, suggesting that longer-duration treatment may be necessary for congestion to manifest. Indeed, we observed no evidence of effect on NT-proBNP at the 3-week assessment, but, with longer-duration administration, an increase in NT-proBNP became evident (Fig. 1). Similar trends were observed for E/e′. Relaxin is a known vasodilator, which was confirmed by the reductions in total peripheral resistance at 12 weeks and 26 weeks. Acute vasodilation can improve congestion through redistribution of blood volume, but, if this vasodilation triggers a compensatory reduction in sodium excretion, congestion will worsen^18^. This increase in sodium avidity may precipitate sodium retention and plasma volume expansion, as shown in older studies with direct vasodilators^19,20^. Indeed, excessive vasodilation is also known to be a key cause of high-output HF^21^. Such effects may be even greater with relaxin (versus other vasodilators) given the teleologic need for volume expansion in the gravid state, although the specific role of relaxin to promote volume expansion compared to other pregnancy-associated hormones remains unclear^6–8^.

LA reservoir strain was improved with low-dose volenrelaxin but not with higher dosages or pooling dosages. RXFP1 receptors are expressed in atrial tissue, where their activation enhances cAMP–PKA signaling pathways to improve contractile and potentially lusitropic performance^6–8^. This could explain the trend for improved LA function, although it is also possible that Frank–Starling effects from volume loading contributed, which would be consistent with the fact that NT-proBNP levels also increased to the greatest extent in patients randomized to the 25-mg dose of volenrelaxin. Regardless, the deleterious consequences of worsening congestion overrode any favorable effect on LA function that might have been possible.

We observed that kidney function was improved with shorter durations of treatment with volenrelaxin, an effect that was no longer significant by 26 weeks. This is consistent with preclinical studies showing increases in renal plasma flow and GFR^6–8^ as well as human clinical trial evidence with short-term relaxin treatment, where serum creatinine was reduced with 48-hour infusion^14^. We observed that the early favorable effect on eGFR was muted with longer duration of treatment, and there were numerically more renal adverse events. This is consistent with an earlier study of acute intravenous relaxin where potential HF benefits were not found to be related to improvements in renal function^22^. Kidney function is known to worsen with increasing congestion in HF^23^, and it may be that progressive congestion with longer treatment duration might have offset ostensible direct renal vasodilatory effects by the conclusion of the trial. Of note, beneficial effects on eGFR were not coupled with favorable effects on glomerular function, as albuminuria tended to worsen with longer duration of volenrelaxin. This may be related to worsening congestion, as previous studies showed that acute stressors, such as acute myocardial infarction, HF decompensation and even exercise or febrile illness, can be powerful drivers of proteinuria^24–28^.

The major limitation relates to the fact that this trial was stopped early before the target enrollment was achieved, decreasing power. Multiple dosages of volenrelaxin were evaluated, introducing heterogeneity, although there were not clear dose–response relationships or significant treatment interaction by dosage. Plasma volume was estimated using a validated formula, rather than direct measurement, and circulatory congestion was not directly assessed using invasive hemodynamics, which would not be feasible in a trial of this size. However, multiple studies have validated the biomarkers (NT-proBNP) and echocardiographic measures (E/e′) employed here to estimate circulatory congestion^29–31^. This study was not powered to evaluate effects on clinical HF events, so we cannot reach any conclusions regarding potential effects on hard outcomes. We evaluated an advanced HFpEF cohort with severe congestion, cardiac dysfunction and sodium avidity, as we hypothesized that potentially favorable effects of volenrelaxin might be most apparent in this cohort. Ongoing trials testing other agents in the class will provide more insight into effects in patients with less advanced HF^32^. Despite these limitations, this trial has several notable strengths, including the novelty of the intervention, which has shown promise in shorter trials but never been evaluated for this sustained duration, and the robust, multimodal assessment of cardiorenal function and hemodynamics, using complementary blood biomarkers and echocardiography, with multiple serial repeated measurements to provide greater physiologic insight.

Despite some evidence for improvement in LA function at low dose, treatment with the long-acting human relaxin agonist volenrelaxin was associated with worsening congestion across multiple independent imaging, laboratory and clinical domains in patients with recently decompensated HFpEF. These data do not support volenrelaxin as a useful therapy in this setting.

## Methods

The RELAXIN-LA (A Phase 2, Randomized, Double-Blind, Placebo-Controlled Study to Investigate the Efficacy and Safety of LY3540378 in Adults with Worsening Chronic Heart Failure with Preserved Ejection Fraction) trial protocol and the statistical analysis plan are included in the online supplement. The ethics committee at each investigative site approved the trial, and all patients provided written informed consent. The ClinicalTrials.gov identifier is NCT05592275. The sponsor was Eli Lilly and Company.

### Study patients

Patients were aged 18 years or older who had chronic NYHA class II–IV HF, left ventricular ejection fraction of 50% or higher and a recent (within 2 weeks) hospitalization for HF or treatment for an urgent visit for worsening HF with intravenous diuretics. Patients were also required to fulfill the following additional criteria: (1) elevated NT-proBNP (>300 pg ml^−1^ in sinus rhythm or >600 pg ml^−1^ in atrial fibrillation or flutter) or elevated B-type natriuretic peptide (BNP, >100 pg ml^−1^/>200 pg ml^−1^); (2) chronic treatment with a loop diuretic prior to recent worsening but transitioned from intravenous to oral loop diuretics at the time of enrollment; and (3) eGFR > 20 ml min^−1^ 1.73 m^−2^. Full eligibility criteria are detailed in the online supplement.

### Study procedures

After a 2-week screening period, eligible patients were randomized double blind (1:1:1:1) to receive volenrelaxin 25 mg, volenrelaxin 50 mg or volenrelaxin 100 mg per week or placebo subcutaneously, in addition to usual therapy. In April 2024 (after 225 participants had been randomized), a modification was made to randomize 1:2:2:2 to ensure greater representation from the volenrelaxin 50 mg and volenrelaxin 100 mg groups based upon the presumption that efficacy would be greater with higher rather than lower dosages. Randomization was performed by using a permuted block design using an interactive web response system and was stratified by (1) presence of atrial fibrillation or atrial flutter on screening electrocardiogram and (2) region of enrollment.

After randomization, patients were evaluated at weekly office or telehealth study visits for 12 weeks and then biweekly, where vital signs, physical examination, NYHA classification, assessments of HF symptoms and major worsening HF events, changes in HF medications and adverse events for 26 weeks were obtained during the double-blind treatment period, with an additional safety follow-up visit 4 weeks later. Laboratory results, including hematology, chemistries and biomarkers, were assessed at screening, randomization and subsequent study visits.

Cystatin C was determined using a turbidimetric assay (Roche cobas chemistry analyzer). eGFR was determined by the Chronic Kidney Disease Epidemiology Collaboration (CKD-EPI) method from creatinine and cystatin C^33^. Urine albumin was measured using a turbidimetric assay; urine creatinine was measured by colorimetric assay; and GGT was measured using the Roche cobas 8000 chemistry analyzer. UACR was calculated. Myocardial injury was assessed by high-sensitivity troponin T. Transthoracic two-dimensional and Doppler echocardiography was performed at baseline, at week 12 and at week 26 according to current guidelines^34,35^ and interpreted in a blinded fashion at a central core laboratory.

### Prespecified primary and secondary endpoints

The primary endpoint of this mechanistic trial was LA reservoir strain, measured by speckle tracking echocardiography^36,37^. LA reservoir strain is a robust, non-invasive indicator of LA filling pressures in HF^38,39^ that quantifies the global ability of the LA to act as a conduit, reservoir and booster pump and was shown in previous studies to improve after treatment for decompensated HF in relation to the extent of decongestion^40^. LA reservoir strain is more strongly correlated with LA pressures than LA volume index and is also more strongly associated with risk for worsening HF events^39,41^. Because relaxin was shown in previous studies to have arterial and venous vasodilatory effects and to reduce LA pressures^12,17^, and because the receptors RXFP1 are expressed in atrial myocardium^6–8^, change in LA reservoir strain at 26 weeks was selected as the primary endpoint of the trial. Secondary endpoints included measures of hemodynamic congestion, specifically changes in NT-proBNP and LA volumes, and measures of kidney function, specifically changes in eGFR, serum creatinine and cystatin C, as well as safety endpoints.

### Tertiary endpoints

Multiple additional mechanistic measures of hemodynamic congestion were evaluated by echocardiography, including left ventricular end diastolic volume, left ventricular global longitudinal strain and LA filling pressure estimated by the E/e′ ratio. Cardiac output was measured by the product of stroke volume (determined by pulsed wave Doppler echocardiography) multiplied by heart rate. NYHA class was evaluated as a measure of physician-rated HF severity. Patient-reported symptom severity and health impairment were quantified by the KCCQ-TSS, the KCCQ-CSS and the KCCQ-OSS^42^.

HF hospitalizations, urgent outpatient HF visits and death (both cardiovascular and non-cardiovascular) were adjudicated by an independent blinded clinical endpoint committee. Urgent HF visits included any urgent outpatient visit, unscheduled office visit or emergency room visit for HF and did not require treatment with intravenous diuretics to fulfil this definition. Scheduled outpatient visits where intravenous diuretics were administered were also classified as urgent HF visits. Other non-fatal cardiovascular events, including myocardial infarction, hospitalization for unstable angina, coronary intervention and cerebrovascular events (stroke or transient ischemic attack), were also adjudicated by the clinical endpoint committee.

### Post hoc exploratory endpoints

Non-prespecified exploratory endpoints included to enhance understanding of physiological effects of treatment included estimated plasma volume, calculated from hematocrit, sex and body weight as (1 − hematocrit) × (a + (b × weight in kg)), where a = 1,530 for men and 864 for women and b = 41 for men and 47.9 for women^43^, and total peripheral resistance, calculated as the quotient of mean arterial pressure and cardiac output determined by echocardiography. To simplify presentation, we also performed a mechanistic analysis comparing placebo-treated to volenrelaxin-treated patients pooling the 25-mg, 50-mg and 100-mg dosages together.

### Statistical methods

The sample size was calculated based on the primary efficacy estimand and its endpoint: change from baseline at week 26 in LA reservoir strain. The goal enrollment included approximately 456 participants randomly assigned to volenrelaxin 25 mg:50 mg:100 mg:placebo (ratio was 1:1:1:1, changed to 1:2:2:2 in April 2024). Assuming a 20% dropout rate, this would result in approximately 91 completers in each of 50-mg, 100-mg and placebo groups. The evaluation of superiority to placebo was to be conducted for volenrelaxin at doses of 25 mg, 50 mg and 100 mg and combination of 50 mg and 100 mg. No adjustment for multiplicity was performed, and *P* values should, therefore, be interpreted cautiously. Assuming an s.d. in LA reservoir strain of 8.5% and a two-sided *α* level of 0.05, 91 completers for 50-mg, 100-mg and placebo groups was calculated to provide 88% power to detect a treatment difference of 4% or more for the primary endpoint.

Analysis of efficacy endpoints, including primary and secondary endpoints, was guided by ‘efficacy estimand’, as this phase 2 study aimed to assess the efficacy of volenrelaxin under the ideal condition that all participants adhere to the randomized treatment. Data collected after permanent discontinuation of study drug were excluded. The sponsor identified a serious breach of protocol from one site in November 2023, in which all enrolled participants were deemed not to meet a key inclusion criterion. Participants from that site (*n* = 26) were excluded from efficacy analysis. For safety analysis, including adverse events, laboratory data except those in primary and secondary endpoints and vital signs, all collected data from randomized patients exposed to at least one dose of volenrelaxin or placebo were included.

The analysis model for comparisons among treatment groups with respect to continuous measurements assessed over time was a mixed model for repeated measures (MMRM) with terms for treatment allocation, study visit, treatment-by-visit interaction, baseline value, stratification strata defined by region (North America, Latin America, Europe and other countries and Asia) and atrial fibrillation or atrial flutter on the screening electrocardiogram and sex. For parameters with skewed distribution (for example, NT-proBNP), MMRM was first applied to the log-transformed values. Least square means of change from baseline and relative difference between volenrelaxin versus placebo were calculated in log scale and then back-transformed to percentage change from baseline and relative percentage difference versus placebo. The primary efficacy comparisons for primary and secondary endpoints are based on the contrast between volenrelaxin doses of 25 mg, 50 mg and 100 mg; combination of 50 mg and 100 mg; and placebo for the absolute changes from baseline to week 26. In post hoc mechanistic analyses, all doses of volenrelaxin were combined and compared to placebo to explore mechanistic effects of treatment on measures of circulatory congestion, hemodynamics and cardiorenal function.

Up to three interim analyses were planned when between 40% and 80% of the participants completed week 26 or discontinued the study. The interim analysis of safety, pharmacokinetics and efficacy measures was reviewed by an assessment committee, independent of the steering committee, to provide recommendations to the sponsor of modifications, no modifications or halting of the trial.

### Reporting summary

Further information on research design is available in the Nature Portfolio Reporting Summary linked to this article.

## Online content

Any methods, additional references, Nature Portfolio reporting summaries, source data, extended data, supplementary information, acknowledgements, peer review information; details of author contributions and competing interests; and statements of data and code availability are available at 10.1038/s41591-025-03939-6.

## Supplementary information

Supplementary Information This includes the study protocol and the statistical analysis plan.

Reporting Summary

## Figures and Tables

**Extended Data Table 1:**
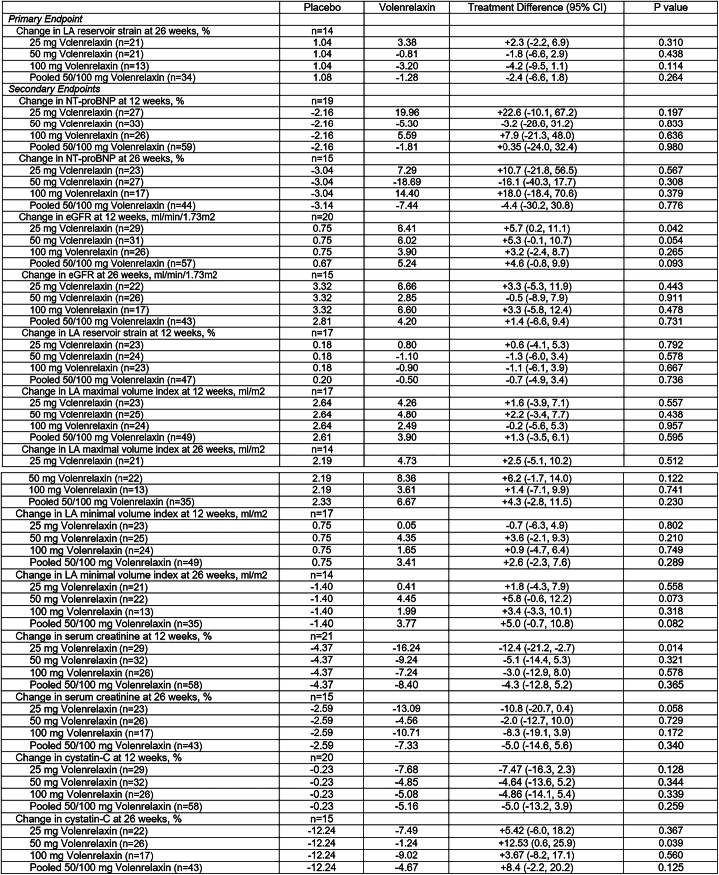
Effects of volenrelaxin on primary and secondary endpoints for females

Treatment comparison is assessed using two-sided *t*-test based on least square means from MMRM. No adjustment for multiplicity was performed. For NT-proBNP, log transformation is applied first on the data, and then the estimation of change from baseline in log scale is transferred back into percentage from baseline in original scale.

**Extended Data Table 2:**
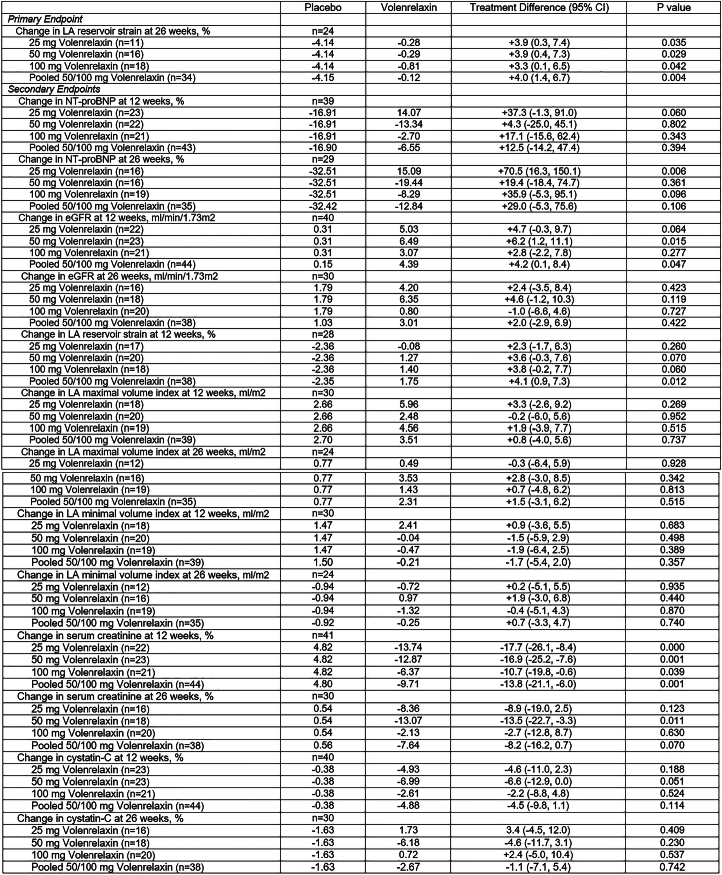
Effects of volenrelaxin on primary and secondary endpoints for males

Treatment comparison is assessed using two-sided *t*-test based on least square means from MMRM. No adjustment for multiplicity was performed. For NT-proBNP, log transformation is applied first on the data, and then the estimation of change from baseline in log scale is transferred back into percentage from baseline in original scale.

**Extended Data Table 3:**
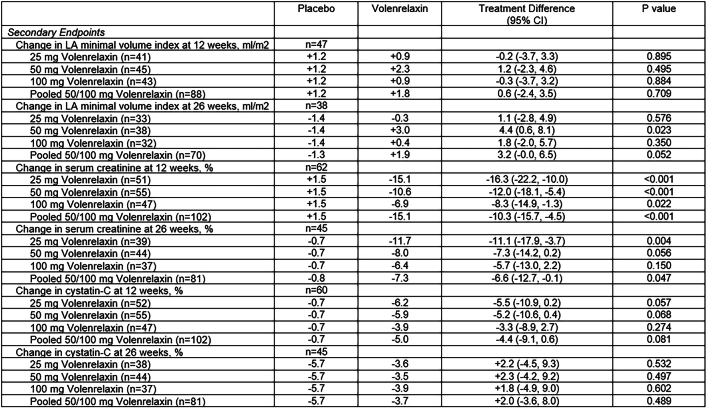
Effects of volenrelaxin on additional secondary endpoints

Treatment comparison is assessed using two-sided *t*-test based on least square means from MMRM. No adjustment for multiplicity was performed. For NT-proBNP, log transformation is applied first on the data, and then the estimation of change from baseline in log scale is transferred back into percentage from baseline in original scale.

**Extended Data Table 4:**
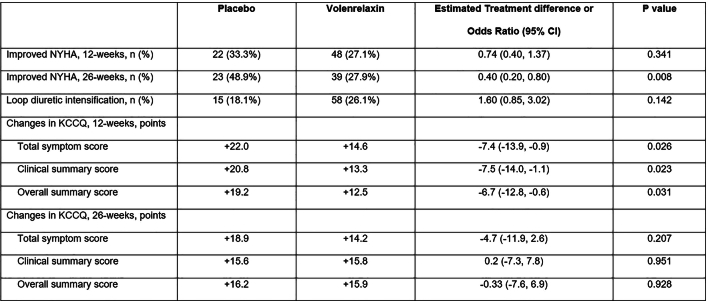
Effects of volenrelaxin (pooled dosages) on HF symptom severity and medication use

Treatment comparison is assessed using two-sided *t*-test based on least square means from MMRM. No adjustment for multiplicity was performed. For categorical variables, including improved NYHA class and loop diuretic intensification, treatment comparison is assessed using OR by Fisher’s exact test or chi-square test, as appropriate.

**Extended Data Table 5:**
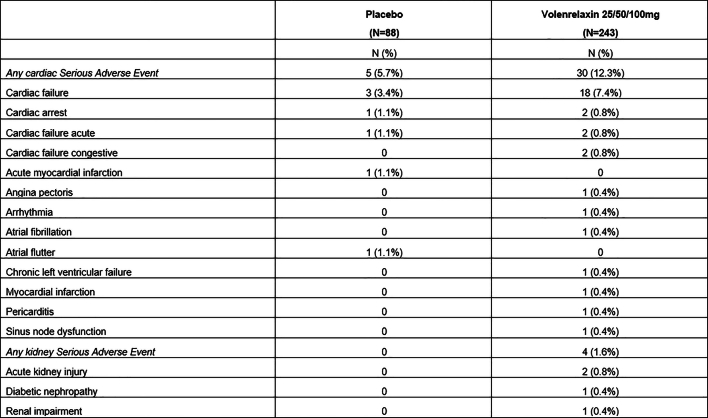
Specific cardiac and kidney serious adverse events by type

Number (*n*) and percentage of patients are shown.

## Data Availability

Eli Lilly and Company provides access to all individual participant data collected during the trial, after anonymization, except for pharmacokinetic or genetic data. Data are available to request 6 months after the indication studied has been approved in the United States and the European Union and after primary publication acceptance, whichever is later. No expiration date of data requests is currently set once data have been made available. Access is provided after a proposal has been approved by an independent review committee identified for this purpose and after receipt of a signed data-sharing agreement. Data and documents, including the study protocol, statistical analysis plan, clinical study report and blank or annotated case report forms, will be provided in a secure data-sharing environment. For details on submitting a request, see the instructions provided at https://vivli.org/.
