# Supplementary material for: Effects of volenrelaxin in worsening heart failure with preserved ejection fraction: a phase 2 randomized trial
Source: Nat Med. 2025 Aug 31;31(11):3853–61. doi: 10.1038/s41591-025-03939-6 (PMC12618225; doi:10.1038/s41591-025-03939-6)
Supplement: Supplementary file 1 — This includes the study protocol and the statistical analysis plan. [file 41591_2025_3939_MOESM1_ESM.pdf]

nature medicine

Supplementary information https://doi.org/10.1038/s41591-025-03939-6

Effects of volenrelaxin in worsening heart failure with preserved ejection fraction: a phase 2 randomized trial

In the format provided by the authors and unedited

Statistical Analysis Plan (J3E-MC-EZDB): A Phase 2, Randomized, Double-Blind, Placebo-Controlled Study to Investigate the Efficacy and Safety of LY3540378 in Adults with Worsening Chronic Heart Failure with Preserved Ejection Fraction (HFpEF)

Protocol Title: A Phase 2, Randomized, Double-Blind, Placebo-Controlled Study to Investigate the Efficacy and Safety of LY3540378 in Adults with Worsening Chronic Heart Failure with Preserved Ejection Fraction (HFpEF)

Protocol Number: J3E-MC-EZDB

Compound: LY3540378

Brief Title: Efficacy and Safety of LY3540378 in Adults with Worsening Chronic Heart Failure with Preserved Ejection Fraction

Sponsor Name: Eli Lilly and Company

Legal Registered Address: Indianapolis, Indiana, USA
46285

Regulatory Agency Identifier Numbers

IND: 152593

EudraCT Number: 2022-000780-48

EU CT Number: 2023-505902-40-00

Confidential Information

The information contained in this document is confidential and the information contained within it may not be reproduced or otherwise disseminated without the approval of Eli Lilly and Company or its subsidiaries

Note to Regulatory Authorities: This document may contain protected personal data and/or commercially confidential information exempt from public disclosure. Eli Lilly and Company requests consultation regarding release/redaction prior to any public release. In the United States, this document is subject to Freedom of Information Act (FOIA) Exemption 4 and may not be reproduced or otherwise disseminated without the written approval of Eli Lilly and Company or its subsidiaries.

Document ID: VV-CLIN-131968

Table of Contents

Table of Contents 2
Version history 6
1. Introduction 7
1.1. Objectives, Endpoints, and Estimands 7
1.2. Study Design 12
2. Statistical Hypotheses 14
2.1. Multiplicity Adjustment 14
3. Analysis Sets 15
4. Statistical Analyses 16
4.1. General Considerations 16
4.2. Participant Dispositions 23
4.3. Primary Endpoint Analysis 23
4.3.1. Definition of Endpoint 23
4.3.2. Main Analytical Approach 23
4.3.3. Supplemental Analyses 23
4.4. Secondary Efficacy Endpoints Analysis 24
4.4.1. Secondary Efficacy Endpoints 24
4.5. Tertiary Endpoints Analysis 26
4.5.1. Tertiary Endpoints in Selected ECHO Parameters, Lab Variables and Patient Reported Outcomes 26
4.5.2. Clinical outcome events of Heart Failure 30
4.5.3. Outpatient Hemodynamic CV Medications 30
4.5.4. Pharmacokinetic and Pharmacokinetic/Pharmacodynamic Methods 30
4.5.5. Immunogenicity 31
4.5.6. Bayesian Analyses for Dose-Response 31
4.6. Safety Analyses 33
4.6.1. Extent of Exposure 33
4.6.2. Adverse Events 34
4.6.3. Patient Narratives 35
4.6.4. Vital Signs 35
4.6.5. Clinical Laboratory Evaluation 36
4.6.6. Additional Safety Assessments 37
4.7. Other Analyses 40
4.7.1. Subgroup Analyses 40
4.8. Interim Analyses 40

5. Sample Size Determination 42
6. Supporting Documentation...43
6.1. Appendix 1: Demographic and Baseline Characteristics 43
6.2. Appendix 2: Historical Illnesses and Pre-existing Conditions 43
6.3. Appendix 3: Treatment Compliance 43
6.4. Appendix 4: Concomitant Medications...44
6.5. Appendix 5: Important Protocol Deviations...44
6.6. Appendix 6: Searching Criteria for Additional Safety Assessments...............................44
6.7. Appendix 7: The Kansas City Cardiomyopathy Questionnaire Scoring Instructions...45
7. References 50

Table of Contents

Tables **Page**

Table EZDB.1.1. Attributes for Efficacy Estimand of Primary Endpoint 9

Table EZDB.1.2. Attributes of Treatment Policy Estimand of Primary and Secondary Endpoints 11

Table EZDB.4.1. Back-transformation for Analysis Results of Log-transformed Variables...18

Table EZDB.4.2. Baseline and Postbaseline Definitions and Patient Population by Type of Analysis....19

Table EZDB.4.3. Definition of Change from Baseline in Secondary Endpoints 25

Table EZDB.4.4. Tertiary Endpoints Analysis....27

Table EZDB.4.5. Summary Tables and Figures Related to Hepatic Safety 37

Table of Contents

Figure **Page**

Figure EZDB.1.1. Illustration of study design for clinical protocol J3E-MC-EZDB. 13

Version history

This Statistical Analysis Plan (SAP) for Study J3E-MC-EZDB is based on the protocol (b) dated 09 April 2024.

SAP Version History Summary

SAP Version	Approval Date	Change	Rationale
1	See date on Page 1	Not Applicable	Original version

1. Introduction

There are no changes to the analyses described in the protocol.

1.1. Objectives, Endpoints, and Estimands

Objectives	Endpoints
Primary	
To demonstrate that LY3540378 administered SC QW is superior to placebo for improving atrial myopathy in participants with worsening chronic HFpEF	Change from baseline to Week 26 in LARS
Secondary	
To compare the effect of LY3540378 administered SC QW on participants with worsening chronic HFpEF	Change from baseline to Weeks 12 in LARS<br>Change from baseline to Weeks 12 and 26 in<br>• Log-transformed NT-proBNP[a]<br>• LAEDVI<br>• LAESVI<br>• eGFR (CKD-EPI Creatinine-Cystatin equation [Inker et al. 2021])<br>• log-transformed serum creatinine[a], and<br>• log-transformed cystatin-C[a]
To assess safety and tolerability of LY3540378 administered SC QW	• AE overall<br>• safety topics of special interest
Tertiary	
To compare the effect of LY3540378 administered SC QW on participants with worsening chronic HFpEF	• Change from baseline to Weeks 12 and 26 in<br>o LA emptying fraction<br>o LVGLS<br>o E/A<br>o E/e’<br>o LVM (LVMI)<br>o log-transformed high sensitivity troponin, (hs-cTnT)[a]<br>o NYHA class, and<br>o log-transformed BNP[a]<br>• Change from baseline to the average of Week 24 and Week 26 in log-transformed NT-proBNP<br>• Blood pressure and pulse rate

Objectives	Endpoints
Clinical outcome events of HF	Incidence of<br>• All deaths (CV and non-CV)<br>• HF event:<br>○ hospitalized (HF hospitalization) and<br>○ non-hospitalized HF events (urgent outpatient visits, unscheduled office, or emergency visit for HF)
Change in outpatient hemodynamic CV medications	Change in the dosing of<br>• diuretics (loop and thiazide)<br>• RAAS inhibitor<br>• SGLT-2i<br>• Beta blockers<br>• ARNI, and<br>• MRA
To assess the effect of LY3540378 on patient-reported outcomes	Change from baseline through Week 26 of<br>• Severity of Most Bothersome Symptom (either dyspnea, edema, or fatigue)<br>• Dyspnea NRS<br>• Edema NRS<br>• Fatigue NRS<br>• PGIS-HF Overall Health<br>• PGIC-HF Overall Health<br>• PGIS-HF Symptom Severity<br>• PGIC-HF Symptom Severity, and<br>• KCCQ<br>○ Total Symptom Score<br>○ Clinical Summary Score, and<br>○ Overall Summary Score
To assess presence of anti-LY3540378 antibodies	ADAs against LY3540378 including<br>• treatment-emergent ADAs, and<br>• neutralizing antibodies
To assess LY3540378 PK and the relationship between LY3540378 dose or exposure and clinical endpoints and potential participant factors that may influence these relationships	PK parameters of LY3540378 (C_{max}, AUC).<br>Dose or exposure-response analyses for key efficacy and safety endpoints

Abbreviations: ADA = anti-drug antibody; AE = adverse event; ARNI = angiotensin receptor neprilysin inhibitor; BNP = brain natriuretic peptide; CV = cardiovascular; ECG = electrocardiogram; eGFR = estimated glomerular filtration rate; HF = heart failure; HFpEF = heart failure with preserved ejection fraction; KCCQ = Kansas City Cardiomyopathy Questionnaire; LA = left atrium; LAEDVI = left atrial end-diastolic volume index; LAESVI = left atrial end-systolic volume index; LARS = left atrial reservoir strain; LVGLS = left ventricular global longitudinal strain; LVM = left ventricular mass; LVMI = left ventricular mass index; MRA = mineralocorticoid receptor antagonists; NRS = Numeric Rating Scale; NT-proBNP = N-terminal pro-B-type natriuretic peptide; NYHA = New York Heart Association; PGIC-HF = Patient Global Impression of Change – Heart Failure; PGIS-HF = Patient Global Impression of Status – Heart Failure; PRO = patient-reported outcome; RAAS = Renin-angiotensin-aldosterone system; SC = subcutaneous; SGLT-2i = sodium-glucose cotransporter-2 inhibitor; QW = weekly.

a: For NTproBNP, serum creatinine, cystatin-C, BNP and hs-cTnT, observations will be log-transformed first prior to conducting analysis. Then results will be back-transformed to percentage difference in the original scale. Refer to Section 4.1 for details.

Primary estimand

The primary clinical question of interest is

> What is the treatment difference in LARS change from baseline after 26 weeks of treatment in study participants who would have completed the treatment period?

Efficacy estimand attributes

Table EZDB.1.1 describes the efficacy estimand attributes.

Table EZDB.1.1. Attributes for Efficacy Estimand of Primary Endpoint

Efficacy Estimand Attribute	Description
Population	Participants who meet the inclusion criteria.<br>Further details can be found in Sections 5 and 9. of the protocol J3E-MC-EZDB(b).
Endpoint	Change from baseline in LARS at Week 26.
Treatment condition	The randomized treatment with allowance for dose modification based on hypotension and temporary discontinuation for safety (Section 6.5 and 7.1.4 of the protocol J3E-MC-EZDB(b)).
Population-level summary	Difference in mean absolute changes in LARS at Week 26 between LY3540378 and placebo.

Abbreviations: LARS = left atrial reservoir strain; TD = temporary discontinuation.

Intercurrent events

The intercurrent event, "permanent discontinuation of intervention," is handled by the hypothetical strategy. The potential outcome of interest is the response in the efficacy measurement if participants adhere to the randomized treatment.

Rationale for the efficacy estimand

This Phase 2 study aims to study the efficacy of LY3540378 under the ideal condition that all participants adhere to the randomized treatment.

Estimand(s) for Secondary Objectives

The same estimand for the primary objective will be used for the following efficacy endpoints for the secondary objective:

- Change from baseline to Weeks 12 in LARS, and
- Change from baseline to Weeks 12 and 26 in
  - Log-transformed NT-proBNP
  - LAEDVI
  - LAESVI
  - eGFR (calculated by creatinine and cystatin-C)
  - log-transformed serum creatinine, and
  - log-transformed cystatin-C

Unless specified otherwise, safety and tolerability assessments will be guided by an estimand comparing safety of LY3540378 doses with placebo irrespective of adherence to study intervention, including data collected during the treatment period and safety follow-up from all randomized participants who are exposed to at least 1 dose of study drug, regardless of adherence of study drug.

Estimand(s) in Exploratory Analyses

The “treatment policy” estimand, which represents the efficacy irrespective of adherence to study intervention, will also be used to compare the efficacy of LY3540378 doses with placebo for primary and secondary endpoints in the exploratory analyses, which includes:

Change from baseline to Weeks 12 and 26 in

- LARS
- log-transformed NT-proBNP
- LAEDVI
- LAESVI
- eGFR (calculated by creatinine and cystatin-C)
- log-transformed serum creatinine, and
- log-transformed cystatin-C

The clinical question of interest in the exploratory analyses is: What is the intervention difference in change from baseline at Week 12 and 26 of the primary and secondary endpoints in participants who meet the inclusion criteria regardless of treatment discontinuation for any reason?

Treatment policy estimand attributes

Table EZDB.1.2 describes the treatment policy estimand attributes.

Table EZDB.1.2. Attributes of Treatment Policy Estimand of Primary and Secondary Endpoints

Efficacy Estimand Attribute	Description
Population	Participants who meet the inclusion criteria.<br>Further details can be found in Sections 5 and 9. of the protocol J3E-MC-EZDB(b).
Endpoints	Change from baseline to Weeks 12 and 26 in<br>• LARS<br>• log-transformed NT-proBNP<br>• LAEDVI<br>• LAESVI<br>• eGFR (CKD-EPI Creatinine-Cystatin equation [2021])<br>• log-transformed serum creatinine[a], and<br>• log-transformed cystatin-C[a]
Treatment condition	The randomized treatment with allowance for dose modification based on hypotension and temporary discontinuation for safety (Section 6.5 and 7.1.4 of the protocol J3E-MC-EZDB(b)).
Population-level summary	Difference in mean changes between LY3540378 and placebo[a].

Abbreviations: ADA = anti-drug antibody; AE = adverse event; ARNI = angiotensin receptor neprilysin inhibitor; BNP = brain natriuretic peptide; CKD-EPI = Chronic Kidney Disease Epidemiology Collaboration; CV = cardiovascular; ECG = electrocardiogram; eGFR = estimated glomerular filtration rate; HF = heart failure; HFpEF = heart failure with preserved ejection fraction; KCCQ = Kansas City Cardiomyopathy Questionnaire; LA = left atrium; LAEDVI = left atrial end-diastolic volume index; LAESVI = left atrial end-systolic volume index; LARS = left atrial reservoir strain; LVGLS = left ventricular global longitudinal strain; LVM = left ventricular mass; LVMI = left ventricular mass index; MRA = mineralocorticoid receptor antagonists; NRS = Numeric Rating Scale; NT-proBNP = N-terminal pro-B-type natriuretic peptide; NYHA = New York Heart Association; PGIC-HF = Patient Global Impression of Change – Heart Failure; PGIS-HF = Patient Global Impression of Status – Heart Failure; PRO = patient-reported outcome; RAAS = Renin-angiotensin-aldosterone system; SC = subcutaneous; SGLT-2i = sodium-glucose cotransporter-2 inhibitor; QW = weekly. LARS = left atrial reservoir strain; TD = temporary discontinuation.

a For NTproBNP, serum creatinine and cystatin-C, observations will be log-transformed first prior to conducting analysis. Then results will be back-transformed to percentage difference in the original scale. Refer to Section 4.1 for details

Intercurrent events

The intercurrent event, “permanent discontinuation of intervention,” is handled by the treatment policy strategy, meaning all the observed values for the variable of interest are used regardless of whether or not the intercurrent event occurs.

Rationale for the treatment policy estimand

This estimand aims to study the efficacy of LY3540378 that reflects the real-life behavior of the target population.

1.2. Study Design

Study EZDB is a Phase 2, multicenter, randomized, double-blind, placebo-controlled study that will investigate the effects of treatment with LY3540378 compared with placebo on participants with worsening chronic HFpEF.

For participants who joined study before amendment (b), they are randomized 1:1:1:1 to the following intervention groups:

- LY3540378 25 mg SC QW
- LY3540378 50 mg SC QW
- LY3540378 100 mg SC QW, and
- Placebo.

For participants who joined study after amendment (b) being active, participants will be randomized 1:2:2:2 to the above intervention groups.

Intervention administration is by subcutaneous injection, and dosing will occur every week.

The maximum total duration of study participation for each participant, including screening and safety follow-up periods, is approximately 32 weeks, across the following study periods:

- Screening: up to 14 days
- Double-Blind Treatment: 26 weeks, and
- Safety Follow-Up: 4 weeks.

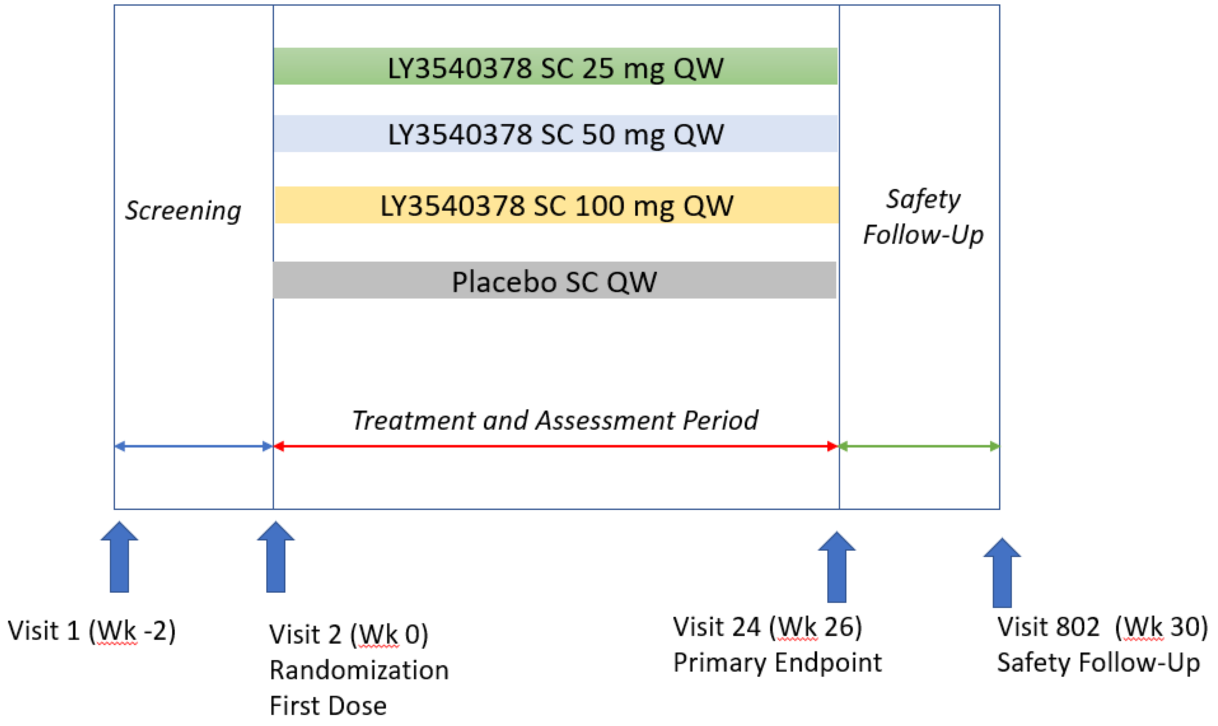

Figure EZDB.1.1. Illustration of study design for clinical protocol J3E-MC-EZDB.

2. Statistical Hypotheses

The primary objective is to demonstrate that LY3540378 administered SC QW is superior to placebo for change from baseline in LARS at Week 26 in participants with worsening chronic HFpEF. Thus, the null hypothesis to be tested in relation to the primary estimand is as follows:

- Null hypothesis: LY3540378 is not different from placebo with respect to change from baseline in LARS at Week 26.

The null hypotheses corresponding to the secondary estimands are as follows:

- LY3540378 is not different from placebo with respect to change from baseline to Week 12 in LARS.
- LY3540378 is not different from placebo with respect to change from baseline to Week 12 or Week 26 in:
  - log-transformed NT-proBNP
  - LAEDVI
  - LAESVI
  - eGFR (calculated by creatinine and cystatin-C)
  - log-transformed serum creatinine, and
  - log-transformed cystatin-C

2.1. Multiplicity Adjustment

No adjustment for multiplicity will be performed.

3. Analysis Sets

This table defines the analysis population and datasets for the purposes of analysis.

Participant Analysis Set	Description
Screened	All participants who signed informed consent.
Randomized	All participants who are randomly assigned to a treatment arm.
Efficacy Analysis Set (EAS)	Data obtained during the treatment period from all randomly assigned participants who are exposed to at least 1 dose of intervention. Participants from site 86127 will be excluded. Excludes data after permanent discontinuation of intervention. Participants will be included in the treatment group to which they were randomly assigned.
Full Analysis Set (FAS)	Data obtained during the treatment period from all randomly assigned participants who are exposed to at least 1 dose of intervention, regardless of adherence to intervention. Participants from site 86127 will be excluded. Participants will be included in the treatment group to which they were randomly assigned.
Safety Analysis Set (SS)	Data obtained during the treatment period plus safety follow-up from all randomly assigned participants who are exposed to at least 1 dose of intervention, regardless of adherence to intervention. Participants will be included in the treatment group to which they were randomly assigned.

4. Statistical Analyses

4.1. General Considerations

Statistical analysis of this study will be the responsibility of Eli Lilly and Company (Lilly) or its designee. Some analyses and summaries described in this analysis plan may not be conducted if not warranted by data (for example, few events to justify conducting an analysis). Additional analyses of the data may be conducted as deemed appropriate.

Unless otherwise noted, tests of treatment effects will be conducted at a 2-sided alpha level of 0.05, and the confidence interval (CI) will be calculated at 95% 2-sided. All tests of interactions between treatment groups and other factors will be conducted at a 2-sided alpha level of 0.10.

All participants from site 86127 will be excluded from the analysis for primary and secondary objectives as the site was terminated following the identification of a serious and egregious breach of protocol. All participants in the study were deemed not to meet key inclusion criteria 2. Detailed information was documented in the 86127 Serious Breach Assessment 2 02 Nov 2023 in eTMF.

Unless otherwise specified, the efficacy analysis will be conducted using Efficacy Analysis Set (EAS) and the safety analysis will be conducted using Safety Analysis Set (SS).

Unless stated otherwise, statistical summaries and analyses will be conducted based on planned randomized treatment group (Placebo, LY3540378 25 mg, LY3540378 50 mg and LY3540378 100 mg, regardless of the actual treatment(s) received by the participant due to any dose modification. The evaluation of the efficacy and safety endpoints will be conducted for LY3540378 25 mg, LY3540378 50 mg and LY3540378 100 mg and compared with placebo.

The primary estimand (a precise definition of the treatment effect to be estimated) of interest in comparing efficacy of LY3540378 doses with placebo is the "efficacy estimand" (Section 1.1). The primary efficacy assessment, guided by the "efficacy estimand" will be conducted using the EAS. A restricted maximum likelihood-based, mixed-effect model repeated measures (MMRM) analysis will be used to analyze continuous longitudinal variables. All the longitudinal observations at each scheduled postbaseline visit will be included in the analysis. The model for primary efficacy endpoint of change from baseline in LARS will include Change from baseline in LARS as the dependent variable. Independent variables include the fixed class effects of treatment group (LY 25 mg, LY 50 mg, LY 100 mg and placebo), visit, treatment-by-visit interaction, gender (male, female), continuous baseline value of LARS, stratification strata defined by region (North America, Latin America, Europe and other countries, Asia) and atrial fibrillation or atrial flutter on the screening ECG (Yes, No), treatment-by-baseline interaction, treatment-by-stratum interaction. An unstructured covariance structure will be used to model the within-participant errors. If this analysis fails to converge, the following covariance structures will be tested in order:

- Heterogeneous toeplitz
- Heterogeneous autoregressive(1)

- Heterogeneous compound symmetry
- Toeplitz
- Autoregressive(1), and
- Compound symmetry.

The first covariance structure that converges will be used. Comparisons of difference in LARS change from baseline for each treatment group of LY3540378 versus placebo reference group will be made by using contrasts of LS means.

Patients in the 100 mg group are allowed to lower the dose level to 50 mg (Section 6.5 of the protocol). For the primary and secondary endpoints, an additional analysis will be conducted to pool the two highest doses (50 mg and 100 mg) together and compare with placebo group. The same above statistical model will be used as the primary analysis except that the fixed class effects will be 25 mg, pooled 50/100 mg, and placebo groups.

Baseline is defined as the last nonmissing measurement recorded on or before the randomization visit, prior to the first dose of intervention, unless otherwise specified. Table EZDB.4.2 summarizes the definition of baseline, postbaseline, and patient population for different endpoints.

Data may exist at visits where the variable was not scheduled to be collected. In these situations, data from the early discontinuation visit that does not correspond to the planned collection schedule will be excluded from the MMRM, analysis of covariance (ANCOVA), or logistic regression analysis.

For laboratory values, both conventional (CN) and International System of Units (SI) units will be presented.

For continuous measures which does not need log transformation, summary statistics will include sample size, mean, standard deviation (SD), minimum, and maximum for both the actual and the change from baseline measurements. LS means and standard errors derived from the analysis models will also be displayed for the change from baseline measurements. Treatment comparisons will be displayed showing the treatment difference LS means and the 95% CIs for the treatment differences, along with the p-values for the treatment comparisons.

For continuous variables that are log-transformed due to skewed distribution (e.g. NTproBNP, BNP, serum creatinine, cystatin-C and hs-cTnT, UACR), summary statistics will include sample size, median, interquartile range (IQR), minimum and maximum for actual value. In addition, log transformation will be applied and then summary statistics including mean and SD will be conducted on log-transformed values. Statistical model will be applied on the log-transformed values. LS means, standard errors, treatment difference LS means and 95% CIs for the treatment differences for the log-transformed variable will be derived and displayed. Then treatment difference and corresponding 95% CIs will be subsequently back-transformed to percentage difference in the original scale. Table EZDB.4.1 lists the back-transformation in details.

Table EZDB.4.1. Back-transformation for Analysis Results of Log-transformed Variables

Quantity		Change from baseline in log-transformed value	Back-transformation to percentage change in original scale
Within Treatment group k	Estimate	$\hat{\mu}_k$	$[\exp(\hat{\mu}_k) - 1] \times 100\%$
	SE	$\widehat{SE}_k$	NR
	95% CI	$(LL_{\mu,k}, UL_{\mu,k})$	$([\exp(LL_{\mu,k}) - 1] \times 100\%, [\exp(UL_{\mu,k}) - 1] \times 100\%)$
Between-Treatment Difference (Treatment k vs. reference placebo r)	Estimate	$\hat{\mu}_{k\ vs\ r}$	$[\exp(\hat{\mu}_{k\ vs\ r}) - 1] \times 100\%$
	SE	$\widehat{SE}_{k\ vs\ r}$	NR
	p-value	$p_{\mu,k\ vs\ r}$	$p_{\mu,k\ vs\ r}$
	95% CI	$(LL_{\mu,k\ vs\ r}, UL_{\mu,k\ vs\ r})$	$([\exp(LL_{\mu,k\ vs\ r}) - 1] \times 100\%, [\exp(UL_{\mu,k\ vs\ r}) - 1] \times 100\%)$

Abbreviations: CI = confidence interval; NR = not reported; SE = standard error.

For categorical measures, summary statistics will include sample size, frequency, and percentages. A logistic regression model may be used to examine the treatment difference in binary efficacy outcomes with missing endpoints imputed. Fisher's exact test or Pearson's chi-square test will be used for treatment comparisons in other categorical outcomes.

Details about the analyses regarding demographic and baseline characteristics, historical illnesses, and preexisting conditions, treatment compliance, concomitant medications, and important protocol deviations can be found in Appendices 1 through 5 (Section 6.1 through Section 6.5, respectively.

End of study participation for a participant will be the earliest of date of death or date of withdrawal from further participation in the study, or date of safety follow-up visit (Visit 802). For participants considered to be lost-to-follow-up, end of study participation will be the date of lost-to-follow-up reported by the investigator. Participant data included in the database after the last date of study participation will be excluded from statistical analysis.

Statistical treatment comparisons will only be performed between LY3540378 and placebo. Because the trial is not adequately powered to detect differences among LY3540378 doses, comparisons across LY3540378 doses will not be performed unless otherwise specified.

Not all analyses described in this SAP will necessarily be included in the clinical study report (CSR). Any analysis described in this SAP and not provided in the CSR will be available upon request.

Table EZDB.4.2. Baseline and Postbaseline Definitions and Patient Population by Type of Analysis

Analysis Type	Participant Population	Baseline Observations	Postbaseline Observations
26 weeks treatment period plus 4 weeks safety follow-up			
ECHO parameters listed in primary, secondary and tertiary objectives, including LARS, LAEDVI, LAESVI, LA emptying fraction, LVGLS, E/A, E/e', LVM and LVMI (MMRM, EAS for efficacy estimand)	All randomized participants who are exposed to at least 1 dose of study drug and have a baseline and at least 1 post-baseline observation prior to permanent discontinuation of study drug. Participants from site 86127 will be excluded.	Visit 1	Visit 16, Visit 24 prior to permanent discontinuation of study drug
ECHO parameters listed in primary, secondary and tertiary objectives, including LARS, LAEDVI, LAESVI, LA emptying fraction, LVGLS, E/A, E/e', LVM and LVMI (MMRM, FAS for treatment policy estimand)	All randomized participants who are exposed to at least 1 dose of study drug and have a baseline and at least 1 post-baseline observation. Participants from site 86127 will be excluded.	Visit 1	Visit 16, Visit 24 with imputation for participants who had missing values at Visits 16 and 24
Biomarker parameters listed in the secondary objectives, including NT-proBNP, eGFR (CKD-EPI creatinine-cystatin equation [2021]), serum creatinine and cystatin-C (MMRM, EAS for efficacy estimand)	All randomized participants who are exposed to at least 1 dose of study drug and have a baseline and at least 1 post-baseline observation prior to permanent discontinuation of study drug. Participants from site 86127 will be excluded.	Last nonmissing measurement recorded on or before the randomization visit (Visit 2), prior to the first dose of intervention	Visit 3, 5, 10, 14, 16, 18, 22, 24 prior to permanent discontinuation of study drug
Biomarker parameters listed in the secondary objectives, including NT-proBNP, eGFR (CKD-EPI creatinine-cystatin equation [2021]), serum creatinine, cystatin-C (MMRM, FAS for treatment policy estimand)	All randomized participants who are exposed to at least 1 dose of study drug and have a baseline and at least 1 post-baseline observation. Participants from site 86127 will be excluded.	Last nonmissing measurement recorded on or before the randomization visit (Visit 2), prior to the first dose of intervention	Visit 3, 5, 10, 14, 16, 18, 22, 24 with imputation for participants who had missing values at these visits
Biomarker parameters listed in the tertiary objectives, including BNP, high sensitivity troponin (hs-cTnT) (MMRM, EAS for efficacy estimand)	All randomized participants who are exposed to at least 1 dose of study drug and have a baseline and at least 1 post-baseline observation prior to permanent discontinuation of study drug. Participants from site 86127 will be excluded.	Last nonmissing measurement recorded on or before the randomization visit (Visit 2), prior to the first dose of intervention	Visit 3, 5, 10, 14, 16, 18, 22, 24 prior to permanent discontinuation of study drug

Analysis Type	Participant Population	Baseline Observations	Postbaseline Observations
Average of Week 24 and Week 26 in log-transformed NT-proBNP (ANCOVA, EAS)	All randomized participants who are exposed to at least 1 dose of study drug and have a baseline and at least 1 post-baseline observation prior to permanent discontinuation of study drug. Participants from site 86127 will be excluded.	Last nonmissing measurement recorded on or before the randomization visit (Visit 2), prior to the first dose of intervention	Visit 22 and 24
NYHA class (shift analysis, EAS)	All randomized participants who are exposed to at least 1 dose of study drug and have a baseline and at least 1 post-baseline observation prior to permanent discontinuation of study drug. Participants from site 86127 will be excluded.	Last nonmissing measurement recorded on or before the randomization visit (Visit 2), prior to the first dose of intervention	Visit 16 and 24
KCCQ scores (MMRM, EAS)	All randomized participants who are exposed to at least 1 dose of study drug and have a baseline and at least 1 post-baseline observation prior to permanent discontinuation of study drug. Participants from site 86127 will be excluded.	Last nonmissing measurement recorded on or before the randomization visit (Visit 2), prior to the first dose of intervention	Visit 16 and 24
Severity of Most Bothersome Symptom, Dyspnea NRS, Edema NRS, Fatigue NRS, PGIS-HF scores, NRS (MMRM, EAS)	All randomized participants who are exposed to at least 1 dose of study drug and have a baseline and at least 1 post-baseline observation prior to permanent discontinuation of study drug. Participants from site 86127 will be excluded.	Last nonmissing measurement recorded on or before the randomization visit (Visit 2), prior to the first dose of intervention	Visit 4, 11, 14, 16, 18, 20, 22 and 24
Treatment-Emergent Adverse Events (SS)	All randomized participants who are exposed to at least 1 dose of study drug. The baseline period is defined as the start of screening and ends prior to the first dose of study drug (Visit 2).	The baseline period is defined as the start of screening and ends prior to the first dose of study drug (Visit 2).	Starts after the first dose of study drug and end of the study period.

Analysis Type	Participant Population	Baseline Observations	Postbaseline Observations
Treatment-Emergent Abnormal Labs (SS)	All randomized participants who are exposed to at least 1 dose of study drug who have a normal baseline (with respect to the direction being analyzed) and a postbaseline observation	Baseline will be all scheduled and unscheduled measurements recorded during the baseline period as defined above (1.1).	Postbaseline will be defined as above (1.1). All scheduled and unscheduled measurements will be included.
Treatment-Emergent Abnormal Vital Signs (SS)	All randomized participants who are exposed to at least 1 dose of study drug and have a baseline and at least 1 post-baseline observation	The last scheduled non-missing assessment recorded prior to the first dose of study treatment during the baseline period defined above (1.1).	Postbaseline will be defined as above (1.1). Only scheduled visits will be included. The early discontinuation visits are considered scheduled visits.
Change from Last Baseline for Labs (SS)	All randomized participants who are exposed to at least 1 dose of study drug and have a baseline and at least 1 post-baseline observation	The last scheduled non-missing assessment recorded prior to the first dose of study treatment during the baseline period defined above (1.1).	Postbaseline will be defined as above (1.1). Only scheduled visits will be included. The early discontinuation visits are considered scheduled visits.
Change from Last Baseline for Blood Pressure and Pulse Rate (SS)	All randomized participants who are exposed to at least 1 dose of study drug and have a baseline and at least 1 post-baseline observation	Average of triplicate VS measurements recorded on the randomization visit, prior to the first dose of intervention	Postbaseline will be defined as above (1.1). Only scheduled visits with triplicates blood pressure and pulse rate measurements will be included. The early discontinuation visits are considered scheduled visits.
Immunogenicity (SS)	All randomized participants who are exposed to at least 1 dose of study drug and have a baseline and at least 1 post-baseline observation.	Baseline is defined as predose collection at Visit 2.	Postbaseline will be defined as above (1.1). Only scheduled visits will be included. The early discontinuation visits are considered scheduled visits.

Abbreviations: BNP = brain natriuretic peptide; CKD-EPI = Chronic Kidney Disease Epidemiology Collaboration; eGFR = estimated glomerular filtration rate; HF = heart failure; HFpEF = heart failure with preserved ejection fraction; KCCQ = Kansas City Cardiomyopathy Questionnaire; LA = left atrium; LAEDVI = left atrial end-diastolic volume index; LAESVI = left atrial end-systolic volume index; LARS = left atrial reservoir strain; LVGLS = left ventricular global longitudinal strain; LVM = left ventricular mass; LVMI = left ventricular mass index; MRA = mineralocorticoid receptor antagonists; NRS = Numeric Rating Scale; NT-proBNP = N-terminal pro-B-type natriuretic peptide; NYHA = New York Heart Association; PGIC-HF = Patient Global Impression of Change – Heart Failure; PGIS-HF = Patient Global Impression of Status – Heart Failure; PRO = patient-reported outcome.

Note: for the continuous analysis of clinical laboratory tests, unscheduled measurements are excluded from analysis to reduce bias (Computational Science Symposium Development of Standard Scripts and Programming Working Group 2013 [WWW]). The early discontinuation (ED) visits are considered scheduled visits.

4.2. Participant Dispositions

A listing and summary of study disposition for all randomized participants will be provided at the primary database lock and final database lock, respectively. Frequency counts and percentages of all participants screened, randomized, and receiving at least 1 dose of study drug will be presented by treatment groups. A listing and summary of randomized participants not receiving study drug will be provided. All participants who discontinue the study and/or study drug will be identified, and the extent of their participation in the study will be reported. If known, a reason for their discontinuation will be given. The primary reasons for discontinuation will be listed and summarized by treatment groups.

4.3. Primary Endpoint Analysis

The primary efficacy assessment, guided by the "efficacy estimand," will be conducted using the EAS. For the "efficacy estimand," the hypothetical strategy is used to handle the intercurrent event (permanent discontinuation of study drug), so only data collected before the occurrence of any such intercurrent events will be used in the MMRM estimation (Section 4.1). Through the MMRM, the potential efficacy measures (after the intercurrent events) had participants not experienced intercurrent events will be implicitly imputed. The primary efficacy comparison will be based on the contrast between each treatment group of LY3540378 and placebo at Week 26 (Visit 24) from the MMRM analysis of change from baseline in LARS using the EAS. (Section 4.1). The analysis model and selection of covariance structure is described in Section 4.1. Treatment comparisons will be performed for the primary objective at the full significance level of 0.05.

4.3.1. Definition of Endpoint

The primary efficacy measure will be change in LARS from baseline to Week 26. The change in LARS at each nominal visit is defined as:

post baseline LARS [%] – baseline LARS [%].

4.3.2. Main Analytical Approach

Change from baseline in LARS will be analyzed using the MMRM model for the "efficacy estimand" as described in Section 4.1.

4.3.3. Supplemental Analyses

Analysis for pooled 50 and 100 mg

Since patients in the 100 mg group are allowed to lower the dose level to 50 mg (Section 6.5 of the protocol). An additional analysis will be conducted using efficacy estimand and pooling the two highest doses (50 mg and 100 mg) together and compare with placebo group. The same statistical approach will be used except that the fixed class effects will be 25 mg, pooled 50/100 mg, and placebo groups.

Treatment Policy Estimand

A supplemental estimand, "treatment-policy estimand" (Section 1.1), will be conducted using data in the FAS.

Treatment-policy estimand is defined as the treatment difference in the mean change in LARS from baseline at Week 26 between LY3540378 and placebo for the study target population with intercurrent events (ICEs) handled by treatment policy strategy. To estimate the "treatment-policy estimand", multiple imputation will be used to impute the corresponding missing potential outcome according to the following table according the scenarios of missingness:

**Scenarios**	**Assumption for Missingness**	**Methods to Handle Missing Values at Endpoint**
Participant has ICE (permanent discontinuation from study treatment)	Missing not at random. Considers that these participants could not adhere to their assigned treatment and may not benefit from the assigned treatment.	Missing values will be imputed using participants in the same treatment arm with similar intercurrent events but non-missing values (retrieved dropout imputation). In cases where there are not enough retrieved dropouts to provide a reliable imputation model, will impute the missing data using the jump-to-reference (placebo) imputation approach.
Participant has missing values without ICEs	Missing at random	Missing values will be imputed using all non-missing data from the same treatment arm.

Change from baseline in LARS will be analyzed using MMRM model (Section 4.1). The comparison will be based on the contrast between each treatment group of LY3540378 and placebo at Week 26 (Visit 24) from the MMRM analysis of change from baseline in LARS using the FAS. In addition, the comparison will also be conducted between pooled 50/100 mg and placebo. The same statistical approach will be used except that the fixed class effects will be 25 mg, pooled 50/100 mg, and placebo groups.

4.4. Secondary Efficacy Endpoints Analysis

The secondary estimands are described in (Section 1.1). The efficacy analyses for the secondary endpoints will use the EAS and MMRM analysis described in Section 4.1. Decision will be guided by the 2-sided p-values in each objective.

4.4.1. Secondary Efficacy Endpoints

4.4.1.1. Definition of Endpoint(s)

Secondary efficacy endpoints include:Change from baseline to Week 12 in LARSChange from baseline to Week 12 and 26 in the following parameters:Log-transformed NT-proBNP

- LAEDVI
- LAESVI
- eGFR (CKD-EPI creatinine-cystatin equation [2021])
- serum creatinine, and

- cystatin-C.

The change for each of the parameters at each nominal visit is defined in Table EZDB.4.3.

Table EZDB.4.3. Definition of Change from Baseline in Secondary Endpoints

Parameter	Calculation of change from baseline at each nominal visit
Log-transformed NTproBNP[a] NTproBNP[a]	post baseline log(NTproBNP[pg/mLng/L]) – baseline log(NTproBNP[pg/mLng/L])
LAEDVI	post baseline LAEDVI[mL/m2mL/m2] – baseline LAEDVI[mL/m2mL/m2],<br>LAEDVI = LAEDV[mL] / BSA[m2m2],<br>BSA[m2m2] = $0.007184 \times height[cm]^{0.725} \times weight(kg)^{0.425}$, height use value at Visit 1, weight use value at Week 12 (Visit 16) and Week 26 (Visit 24), respectively
LAESVI	post baseline LAESVI[mL/m^2] – baseline LAESVI[mL/m^2],<br>LAESVI = LAESV[mL] / BSA[m^2],<br>BSA is same as that in LAEDVI
eGFR	post baseline eGFR[mL/min/1.73m2mL/min/1.73 m2] – baseline eGFR[mL/min/1.73m2mL/min/1.73 m2]
Log-transformed serum creatinine	post baseline log(creatinine[mg/dLmg/dL]) – baseline log(creatinine [mg/dLmg/dL])
Log-transformed cystatin-C[a]	post baseline log(creatinine[mg/Lmg/L]) – baseline log(creatinine [mg/Lmg/L])

Abbreviations: BSA = body surface area.

[a] For NTproBNP, serum creatinine and cystatin-C, observations will be log-transformed first prior to conducting analysis. Then results will be back-transformed to percentage difference in the original scale. Refer to Section 4.1 for details.

For NTproBNP, serum creatinine, cystatin-C, BNP and hs-cTnT, observations will be log-transformed first prior to conducting analysis. Then results will be back-transformed to percentage difference in the original scale. Refer to Section 4.1 for details.

For NTproBNP, serum creatinine, cystatin-C, BNP and hs-cTnT, observations will be log-transformed first prior to conducting analysis. Then results will be back-transformed to percentage difference in the original scale. Refer to Section 4.1 for details.a: For NTproBNP, serum creatinine, cystatin-C, BNP and hs-cTnT, observations will be log-transformed first prior to conducting analysis. Then results will be back-transformed to percentage difference in the original scale. Refer to Section 4.1 for details

For NTproBNP, serum creatinine, cystatin-C, BNP and hs-cTnT, observations will be log-transformed first prior to conducting analysis. Then results will be back-transformed to percentage difference in the original scale. Refer to Section 4.1 for details.

For NTproBNP, serum creatinine and cystatin-C, the definition of endpoint is based on log-transformed values. Refer to Section 14.1 and Table EZDB.4.1 for analysis details of variables which needs log-transformation.

4.4.1.2. Main Analytical Approach

The analysis of the secondary efficacy endpoints will be conducted using MMRM model described in Section 4.1. Refer to Table EZDB.4.2 for the definition of population, baseline and postbaseline for each of secondary efficacy endpoints.

4.4.1.3. Supplementary Analyses

Analysis for pooled 50 and 100 mg

An additional analysis will be conducted to 50 mg and 100 mg together and compare with placebo group. The same statistical approach will be used except that the fixed class effects will be 25 mg, pooled 50/100 mg, and placebo groups.

Treatment Policy Estimand

A supplemental estimand, "treatment-policy estimand" (Section 1.1), will be conducted for each of the secondary efficacy endpoint using data in the FAS. Same approach as the treatment-policy estimand in Section 4.3.3 will be applied.

4.5. Tertiary Endpoints Analysis

Unless otherwise specified, analyses for tertiary and exploratory endpoint will be conducted for EAS. Decision will be guided by the 2-sided p-values in each objective.

4.5.1. Tertiary Endpoints in Selected ECHO Parameters, Lab Variables and Patient Reported Outcomes

For ECHO parameters, lab variables and patient reported outcomes listed in the tertiary endpoints, the definitions of baseline, postbaseline and patient populations are described in Table EZDB.4.2. For variables which distributions are skewed and need log transformation, refer to Section 4.1 and Table EZDB.4.1 for the detailed analysis approach. Detailed analysis approaches for the tertiary endpoints are described in Table EZDB.4.4.

Table EZDB.4.4. Tertiary Endpoints Analysis

Objectives	Relative to the efficacy measure	Endpoint definition	Analysis conducted
To compare the effect of LY3540378 administered SC QW on participants with worsening chronic HFpEF	Change from baseline to Weeks 12 and 26 in: LA emptying fraction (LAEF), LVGLS, E/A, E/e', LVM, LVMI	LAEF = (LAEDV – LAESV) / LAEDV × 100%,<br>LVMI = LVM / BSA,<br>For each ECHO parameter, change from baseline = post baseline value – baseline value	Same MMRM model as that for the primary estimand in Section 4.1. LSM estimates with 95% CIs for Week 12 and 26 will be plotted by study treatment and by Week.
	Change from baseline to Weeks 12 and 26 in BNP, and hs-cTnT	log(BNP post basline) – log(BNP baseline)<br>log(hs-cTnT) – log(hs-cTnT)	Same MMRM model as in Section 4.1 will be applied. Refer to Section 4.1 and Table EZDB.4.1 for analysis details of variables which needs log-transformation.LSM estimates with 95% CIs through Week 26 will be plotted by study treatment.
	Change from baseline to the average of Week 24 and Week 26 in log-transformed NT-proBNP	(log(NTproBNP at Week 26) + log(NTproBNP at Week 24)) / 2 - log(NTproBNP at baseline)	ANCOVA model will be applied. Model includes fixed class effect of treatment groups (LY 25 mg, LY 50 mg, LY 100 mg and placebo). Additional covariates include stratification strata defined by regions (North America, Latin America, Europe and other countries, Asia) and atrial fibrillation or atrial flutter on the screening ECG (Yes, No), gender (male, female) and continuous covariate of baseline log(NTproBNP) value.<br>The save ANCOVA model will also be conducted for comparison between pooled 50/100mg and placebo. The only difference in the ANCOVA model is the fixed class effects being 25 mg, pooled 50/100 mg, and placebo groups.

Objectives	Relative to the efficacy measure	Endpoint definition	Analysis conducted
			LSM estimates with 95% CIs will be plotted by study treatment.
	Categorical Change from Baseline to Week 12 and Week 26 in NYHA class	Categorical Change from Baseline in NYHA class will be one of the following nominal value:<br>Worsened: post baseline NYHA class is larger than baseline NYHA class<br>Unchanged: post baseline NYHA class is the same as baseline NYHA class<br>Improved: post baseline NYHA class is smaller than baseline NYHA class	The number of subjects and corresponding percentage for worsened case, unchanged case and improved case will be summarized for Week 12 and Week 26.<br>The categorical change in New York Heart Association Class (improved, no change, or worsened) from baseline will be analyzed using a longitudinal proportional odds model. The response variable of the analysis model will be the change in NYHA class from baseline. The independent variables of the model will include the categorical effect of treatment, time, treatment-by-time interaction and the stratification factors, and baseline NYHA class as a covariate. Odds ratio and 95% CI relative to placebo will be reported for improved vs no change or worsened, and for improved or no change vs worsened.<br>For missing NYHA change category data, the category worsened is assigned for death. For other reason of missingness, no imputation will be conducted.
To assess the effect of LY3540378 on patient-reported outcomes	Change from baseline through Week 26 of KCCQ:<br>Total Symptom Score (TSS)<br>Clinical Summary Score, and (CSS)	For each score, the change from baseline at each nominal visit is defined as:<br>post baseline score – baseline score<br>Detailed scoring instructions are provided in Appendix 7 (Section 6.7).	Same MMRM model as in Section 4.1 will be applied. LSM estimates with 95% CIs through Week 26 will be plotted by study treatment.

Objectives	Relative to the efficacy measure	Endpoint definition	Analysis conducted
	Overall Summary Score (OSS)		
	Change from baseline through Week 26 of<br>Dyspnea NRS<br>Edema NRS<br>Fatigue NRS<br>PGIS-HF Overall Health<br>PGIS-HF Symptom Severity	For each PRO, the change from baseline at each nominal visit is defined as:<br>post baseline score – baseline score	Same MMRM model as in Section 4.1 will be applied. LSM estimates with 95% CIs through Week 26 will be plotted by study treatment. Same MMRM model as in Section 4.1 will be applied. LSM estimates with 95% CIs through Week 26 will be plotted by study treatment.
	Most Bothersome Symptom (either dyspnea, edema, or fatigue)	Most Bothersome Symptom at each visit	The Summary of number of subjects and percentage of subjects whose the most bothersome symptom is dyspnea, edema and fatigue respectively in Week 0, Week 12, and Week 26

4.5.2. Clinical outcome events of Heart Failure

The following outcome events will be adjudicated by an independent clinical endpoint committee (CEC) external to Lilly to Lilly with cardiology expertise. CV death (CV and non-CV)

- Death: CV death and non-CV death
- hospitalization for heart failure (HF)
- urgent HF visits (urgent outpatient visits, unscheduled office, or emergency visit for HF)

This committee will be blinded to treatment assignment.

Only adjudicated outcome events will be considered as AESI. The counts and percentages of participants with adjudicated events may be summarized by treatment.

A listing of participants reporting the outcome events, either reported by investigator or identified by the CEC, will be provided. The listing will include treatment, participants identification including the site number, date of event, type of event as reported by the investigator, type of event as adjudicated by the CEC, time from first dose of study drug to the event, and time from last dose to the event (if participant has discontinued study drug prior to the event).

The counts and percentages of participants with the composite endpoint of CV death, hospitalization for HF or urgent HF visits will also be summarized by treatment. Kaplan-Meier plots of time to the composite event will be provided.

4.5.3. Outpatient Hemodynamic CV Medications

The endpoints related to outpatient hemodynamic CV medications are Change in the dosing of each of the following medications:

- diuretics (loop and thiazide)
- RAAS inhibitor
- SGLT-2i
- Beta blockers
- ARNI, and
- MRA.

Categorical change from baseline dose level will be one of the following nominal value: reduced, unchanged, increased. The number of subjects and corresponding percentage for reduced case, unchanged case and increased case will be summarized for each scheduled visit.

4.5.4. Pharmacokinetic and Pharmacokinetic/Pharmacodynamic Methods

Pharmacokinetic (PK), pharmacodynamic (PD), and PK/PD analysis are the responsibility of Lilly's PK/PD group.

LY3540378 concentration-time data will be summarized in the clinical study report.

Dose/exposure-response analyses between LY3540378 dose/concentration and key safety, tolerability, and efficacy points may be explored graphically or performed using population PK and population PK/PD approaches implemented in the Nonlinear Mixed Effects Modeling (NONMEM) software. Additionally, the impact of intrinsic and extrinsic factors (such as age, weight, sex, immunogenicity, renal, and hepatic functions) on PK and/or PD parameters may be evaluated where applicable.

4.5.5. Immunogenicity

At the visits and times specified in the protocol schedule of activities (Section 1.3 of the protocol), venous blood samples will be collected (if local regulations and ethical review boards allow) and stored for potential future analysis to determine antibody production against LY3540378. If the data is available at the time of final database lock, the following analysis will be conducted.

Treatment-emergent antidrug antibodies (TE ADAs) are defined as those with a titer 2-fold (1 dilution) greater than the minimum required dilution if no ADAs were detected at baseline (treatment-induced ADA) or those with a 4-fold (2 dilutions) increase in titer compared to baseline if ADAs were detected at baseline (treatment-boosted ADA). A patient is evaluable for TE ADA if the patient has a nonmissing baseline ADA result, and at least 1nonmissing postbaseline ADA result.

Listings of patients who are not TE ADA evaluable, patients with at least one test having detected LY3437943 ADAs, and patients having LY3540378 ADAs present or TEAE: hypersensitivity reactions or injection site reactions will be provided.

The frequency and percentage of patients with preexisting ADA and with TE ADA will be tabulated by dose (if data warrant), where proportions are relative to the number of patients who are TE ADA evaluable. The frequency and percentage of patients with hypersensitivity and injection site reaction treatment-emergent adverse events (TEAEs) by TE ADA status will be tabulated if data warrant.

4.5.6. Bayesian Analyses for Dose-Response

4.5.6.1. Bayesian Analyses for Dose-Response at Week 12 and 26

For change from baseline in a key efficacy endpoint (e.g. LARS) at Week 26, we assume it satisfies a 3-parameter Emax model:

$$Y_i = E_0 + \frac{E_{max} d_i}{ED_{50} + d_i}$$

Here Y_i is the change from baseline at Week 26 for an efficacy endpoint of subject i, d_i is the dose level received by subject i, E_0 represent the basal effect when the dose level is 0 (placebo), E_{max} represents the maximum effect that can be achieved by any dose level on top of placebo, and ED_{50} is the dose level that produces half of the maximum effect.

The estimation of the parameters will be carried out in a Bayesian framework assuming noninformative priors for the hyperparameters in the model as follows:

$$\begin{cases} E_0 \sim N(0, 100^2), \\ E_{max} \sim N(0, 100^2), \\ ED_{50} \sim N^+(0{,}400), \end{cases}$$

where $N^+(0{,}400)$ is truncated normal distribution by bounding above 0. Posterior inference will be drawn for the dose-response at each dose level of LY3540378 and the 95% credible intervals will also be plotted.

The same Bayesian analysis will be applied for change from baseline at Week 12 for a key efficacy endpoint. Other dose-response model may also be explored if the above Emax model is not suitable.

4.5.6.2. Bayesian Analyses for Longitudinal Dose-Response

The longitudinal dose-response model as proposed by Fu and Manner (2010) will be applied here. Let d_{it} be the dose level taken by subject i at Week t , and Y_{it} be the change from baseline of an endpoint (e.g. LARS) for subject i at Week t. The model is as follows

$$Y_{it} = \beta_0 Baseline_i + f(t;k)(\lambda(d_{it}) + S_i) + \varepsilon_{it}.$$

Function $f(t;k)$ handles the time information and is assumed to be monotone with a pattern of exponential decay:

$$f(t;k) = \frac{1 - \exp(k_d t)}{1 - \exp(k_d T)}$$

where $T = 26$ is the maximum duration of the treatment period in weeks. Function $\lambda(d_{it})$ is the dose-response model for the maximum response at dose d, and assumed to be a 3-parameter Emax model:

$$\lambda(d) = \alpha_0 + \frac{\alpha_1 d}{\alpha_2 + d}$$

Here we use different parameters to distinct them from the Emax model in Section 4.5.6.1. In addition, δ_i is the between-subject random error term, ε_{it} is the within-subject random error term. We assume $S_i \sim N(0, \sigma_S^2)$ and $\varepsilon_{it} \sim N(0, \sigma^2)$ are independent.

The estimation of those parameters will be carried out in a Bayesian framework assuming noninformative priors for the hyperparameters in the model as follows:

$$\begin{cases} k_d \sim \text{uniform}(0,1), \\ \alpha_0 \sim N(0, 100^2), \\ \alpha_1 \sim N(0, 100^2), \\ \alpha_2 \sim N^+(0,400), \\ \frac{1}{\sigma_S^2} \sim Gamma(0.01,0.01), \\ \frac{1}{\sigma^2} \sim Gamma(0.01,0.01). \end{cases}$$

Posterior inference will be drawn for the dose-response at Week t of clinical interest and the 95% credible intervals will also be plotted.

4.6. Safety Analyses

Unless specified otherwise, safety assessments will be guided by an estimand comparing safety of LY3540378 doses with placebo irrespective of adherence to study drug. Thus, safety analyses will be conducted using the safety analysis set.

4.6.1. Extent of Exposure

Listing of exposure to LY3540378 and placebo will be provided by treatment group using data from SS. Summary of duration of follow-up (defined as time in days from date of randomization to the date of the last study visit) and/or duration on study treatment (defined as time in days from date of first dose of study treatment to date of last dose of study treatment plus 7 days) will be provided by treatment group using data from SS, in the following period:

- 26 weeks plus safety follow-up (Visit 801 and 802) for all randomized participants.

For the summary of duration on study treatment, the frequency and percentage of participants falling into the following range will be summarized by planned treatment group as well:

- >0,
- ≥4 weeks,
- ≥8 weeks,
- ≥12 weeks,
- ≥16 weeks,
- ≥20 weeks,
- ≥24 weeks,
- and ≥26 weeks.

In addition, the frequency and percentages of participants falling into the following study treatment exposure ranges may be summarized by planned treatment group:

- >0 to <4 weeks,
- ≥4 to <8 weeks,
- ≥8 to <12 weeks,
- ≥12 to <16 weeks,
- ≥16 to <20 weeks,

- ≥20 to <24 weeks,
- ≥24 to ≤26 weeks.

No p-values will be reported in these summaries as they are intended to describe the study populations rather than test hypotheses about them.

*Temporary Discontinuation (TD) of Study Treatment*Number of subjects and percentage who had TD will be summarized by each treatment group. The number of doses which are not administered per protocol schedule of activities (Section 1.3 of the protocol) subject will also be summarized by treatment group, within those subjects who had TD.Subjects one the highest dose 100 mg are allowed to down titrate dose level to 50 mg. Number of subjects and percentage who had this down titration will be summarized by each treatment group. The number of doses which are down titrated per subject will also be summarized within those subjects who had dose down titration.

4.6.2. Adverse Events

A treatment-emergent adverse event (TEAE) is defined as an event that first occurred or worsened in severity after the first dose. The Medical Dictionary for Regulatory Activities (MedDRA) low level term (LLT) will be used in the treatment-emergent derivation. The maximum severity for each LLT during the baseline period including ongoing medical history will be used as baseline severity. Events with a missing baseline severity will be treated as "mild" in severity for determining treatment-emergence. Events with a missing severity during the postbaseline period will be treated as "severe" and treatment-emergence will be determined by comparing to baseline severity.

For events occurring on the day of first taking study medication, the case report form collected information (for example, treatment emergent flag, start time of study treatment and event) will be used to determine whether the event was pre- versus post-treatment if available. If the relevant information is not available, then the events will be counted as posttreatment.

The counts and percentages of participants with TEAEs will be summarized by treatment using MedDRA preferred terms (PTs) nested within system organ class (SOC). Comparisons will be applied at both the SOC and PT levels. Events will be ordered by decreasing frequency within SOC. The SOC will be in alphabetical order. For events that are sex-specific, the denominator and computation of the percentage will include only participants from the given sex.

An overview of the number and percentage of participants who experienced a TEAE, a serious adverse event (SAE), or death, discontinued from study treatment or study due to an AE, and relationship to study drug will be summarized by treatment.

The counts and percentages of patients with TEAEs by maximum severity will be summarized by treatment using MedDRA PT. For each participant and TEAE, the maximum severity for the MedDRA PT is the maximum postbaseline severity observed from all associated LLTs mapping to the MedDRA PT. The maximum severity will be determined based on the non-missing severities. If all severities are missing for the defined postbaseline period of interest, it will show as missing in the table.

4.6.2.1. AE of Special Interest (AESI)

The following are the AESIs:

- vaginal haemorrhage,
- breast tumors,
- hypotension,
- orthostatic hypotension,tissue changes in the female reproductive tracts, and
- anaemia.

The counts and percentages of patients with each category of AESIs by maximum severity will be summarized by treatment.

4.6.3. Patient Narratives

Patient narratives will be provided for all participants who experience any of the following “notable” events:

- death,
- serious AE,
- pregnancy, or
- permanent discontinuation of study treatment due to AEs.

Patient narratives (patient level data and summary paragraph) will be provided for participants in the randomized population with at least 1 notable event.

4.6.4. Vital Signs

In the case that multiple records of an individual vital sign are collected at the same visit, they will be averaged prior to being used for data summaries and analyses. For example, blood pressure (BP) and pulse rate (PR) are triplicated at Visit 2, 5, 6, 16, 18, 24 and early discontinuation per protocol schedule of activities (Section 1.3 of the protocol). The measurements will be averaged first.

Descriptive summaries by treatment and by nominal visit will be provided for baseline and postbaseline values as well as change from baseline values.

Treatment differences in mean change will be analyzed using the MMRM model as described in Section 4.1 for the safety analysis set.

Counts and percentages of participants with treatment-emergent abnormal supine systolic blood pressure (BP), diastolic BP, and pulse will be presented by treatment for participants who have both baseline and at least 1 postbaseline result:

- Treatment-emergent high result: a change from a value less than or equal to the high limit at baseline to a value greater than the high limit at any time that meets the specified change criteria during the postbaseline period.
- Treatment-emergent low result is defined as a change from a value greater than or equal to the low limit at baseline to a value less than the low limit at any time that meets the specified change criteria during the postbaseline period.

To assess decreases, change from the minimum value during the baseline period to the minimum value during the postbaseline period will be used. To assess increases, changes from the maximum value during the baseline period to the maximum value during the postbaseline period will be used. Both planned and unplanned measurements will be included in the analysis. The criteria for identifying patients with treatment-emergent vital sign abnormalities are stated in

Parameter	Low	High
Systolic BP (mm Hg) (Supine or sitting – forearm at heart level)	≤90 and decrease from baseline ≥20	≥129 and increase from baseline ≥20
Diastolic BP (mm Hg) (Supine or sitting – forearm at heart level)	≤50 and decrease from baseline ≥10	≥90 and increase from baseline ≥10
Pulse (bpm) (Supine or sitting)	<50 and decrease from baseline ≥15	>100 and increase from baseline ≥15

4.6.5. Clinical Laboratory Evaluation

All laboratory data will be reported in SI units. Selected laboratory measures will also be reported using conventional units. Limits from the performing lab will be used to define low (L) and high (H). Descriptive summaries by treatment and by nominal visit will be provided for the baseline and postbaseline values as well as the change from baseline values.

Observed and change from baseline values for each visit may be displayed in plots for participants who have both a baseline and at least 1 postbaseline planned measurement. Baseline will be the last non-missing observation prior to taking first study drug. Unplanned measurements will be excluded from plots.

A shift table will be provided including unplanned measurements. The shift table will include the number and percentage of participants within each baseline category (low, normal, high, or missing) versus each postbaseline category (low, normal, high, or missing) by treatment. The proportion of participants shifted will be compared between treatments using Fisher’s exact test.

For qualitative laboratory analytes, the number and percentage of participants with normal and abnormal values will be summarized by treatment.

A listing of abnormal findings will be created for laboratory analyte measurements, including qualitative measures. The listing will include participant identification, treatment group, laboratory collection date, study day, analyte name, and analyte finding.

The MMRM model or ANCOVA (if MMRM model is not applicable) will be used for the analysis during the treatment period for the continuous measurements for selected lab tests.

4.6.6. Additional Safety Assessments

4.6.6.1. Hepatobiliary Disorders

The counts and percentages of participants with treatment-emergent potentially drug-related hepatobiliary disorders will be summarized by treatment using the PTs nested within Standardized MedDRA Queries (SMQs). Detailed search criteria can be found in Appendix 6 (Section 6.6).

4.6.6.1.1. Liver Enzymes

Analyses for laboratory analyte measurements are described in Section 4.6.5. This section describes additional analyses of liver enzymes.

Hepatic labs include alanine aminotransferase (ALT), aspartate aminotransferase (AST), total bilirubin (TBL), direct bilirubin (DBL), serum alkaline phosphatase (ALP), international normalized ratio (INR), and gamma-glutamyl transferase (GGT). When criteria are met for hepatic evaluations, investigators will conduct close monitoring of hepatic symptoms and liver tests, perform a comprehensive evaluation for alternative causes of abnormal liver tests, and complete follow-up hepatic safety electronic case report form (eCRFs).

The following will be analyzed for hepatic safety (Table EZDB.4.5):

Table EZDB.4.5. Summary Tables and Figures Related to Hepatic Safety

Analysis	Population or Analysis Set
Abnormal Postbaseline Categories – Hepatic Safety Parameters<br>ALT<br>• The number and percentage of participants with a measurement greater than or equal to 1×, 3×, 5×, 10×, and 20× the performing lab ULN during the treatment period for all participants with a postbaseline value.<br>AST<br>• The number and percentage of participants with a measurement greater than or equal to 1×, 3×, 5×, 10×, and 20× the performing lab ULN during the treatment period for all participants with a postbaseline value.<br>ALP<br>• The number and percentage of participants with a measurement greater than or equal to 2× and 3× the performing lab ULN during the treatment period will be summarized for all participants with a postbaseline.<br>TBL<br>• The number and percentage of participants with a measurement greater than or equal to 2×, 5×, and 8× the performing lab ULN during the treatment period will be summarized for all participants with a postbaseline value.<br>DBL<br>• The number and percentage of participants with a measurement greater than or equal to 2× and 5× the performing lab ULN during the treatment period will be summarized for all participants with a postbaseline value.	FAS

Analysis	Population or Analysis Set
GGT<br>• The number and percentage of participants with a measurement greater than or equal to 2× the performing lab ULN during the treatment period will be summarized for all participants with a postbaseline value.	
Hepatocellular Drug-Induced Liver Injury Screening Plot (TBL vs. ALT or AST)	FAS
Hepatocellular Drug-Induced Liver Injury Screening Table	FAS
Cholestatic Drug-Induced Liver Injury Screening Plot (TBL vs. ALP)	FAS
Cholestatic Drug-Induced Liver Injury Screening Table	FAS
Participant profiles will be created for participants meeting criteria for a comprehensive hepatic evaluation (as defined in the protocol).<br>Participant profiles will include demographics, disposition, information collected on the hepatic safety CRFs (where applicable) and a display of study drug exposure, adverse events, medications, blood pressure, heart rate, and the liver-related measurements over time.	FAS

Abbreviations: ALP = alkaline phosphatase; ALT = alanine aminotransferase; AST = aspartate aminotransferase; CRF = case report form; DBL = direct bilirubin; FAS = full analysis set; GGT = gamma-glutamyl transferase; TBL = total bilirubin; ULN = upper limit of normal.

Planned and unplanned measurements will be included. The measurements do not need to be taken at the same blood draw. Maximum baseline will be the maximum nonmissing observation in the baseline period. The maximum value will be the maximum nonmissing value from the postbaseline period. Planned and unplanned measurements will be included.

The primary purpose of the screening plots is to identify participants whose data warrant further review. For these plots, symbols will be used to indicate the randomized treatment.

For individual participants of interest, participant profiles will be reviewed. The review will include which treatment the participant was taking over time, the changes in hepatic labs over time, and the temporal association with potential causes. The review of participant profiles will also include the identification of any potential Hy's law case or potential cholestatic liver injury case that could have been missed by focusing only on the maximum values when determining 30-day time associations.

4.6.6.2. Hypersensitivity Events

Hypersensitivity reactions and related information reported in eCRF will be listed and summarized by treatment.

Summaries of all potential hypersensitivity reactions will be generated by PT with decreasing frequency by treatment. The AE database will be searched using predefined SMQs to identify

events consistent with hypersensitivity events. Detailed search criteria can be found in Appendix 6 (Section 6.6).

4.6.6.3. Injection Site Reactions

Injection site reactions, incidence, and related information reported in eCRFs will be summarized by treatment. Information to be summarized includes the timing of the reaction relative to study drug administration, and characteristics of the injection site reaction: erythema, induration, pain, pruritus, and edema.

Additionally, potential injection site reactions will be searched by predefined MedDRA high level terms (HLTs) of injection site reactions, administration site reactions, and infusion-related reactions. Detailed searching criteria for injection site reaction events can be found in Appendix 6 (Section 6.6). The PT will be used for summary by treatment within each HLT category.

4.6.6.4. Renal Safety

Two shift tables examining renal function will be created:

- A min-to-min shift table of estimated glomerular filtration rate (eGFR) estimated by the Chronic Kidney Disease Epidemiology Collaboration (CKD-EPI) equation with unit mL/min/1.73 m^2, using categories (<30, ≥30 to <45, ≥45 to <60, ≥60 to <90, and ≥90 mL/min/1.73 m^2), and
- Max-to-max shift table of urine albumin-to-creatinine ratio (UACR), using the categories UACR <30 g/kg, 30 g/kg ≤ UACR ≤300 g/kg, and UACR >300 g/kg (respectively, these represent normal, microalbuminuria, and macroalbuminuria).

The MMRM model will be used for the continuous measurements of the log-transformed UACR. Refer to Section 4.1 and Table EZDB.4.1 for analysis details of variables which needs log-transformation.

4.6.6.5. Major Adverse Cardiovascular Events (MACE)

In addition to the clinical outcome event of HF (Section 4.5.2), the following nonfatal cardiovascular AEs (NCAE) will also be adjudicated by CEC:

- myocardial infarction
- hospitalization for unstable angina
- coronary interventions, such as coronary artery bypass graft or percutaneous coronary intervention, and
- cerebrovascular events, including cerebrovascular accident (stroke) and transient ischemic attack.

The counts and percentages of participants with adjudicated NCAE may be summarized by treatment.

In addition, NCAE reported by investigator may also be summarized although an NCAE reported by investigator is not considered as AESI.

A listing of participants reporting NCAE events, either reported by investigators or identified by the aforementioned committee, will be provided. The listing will include treatment, participants' identification, including the site number, date of event, type of event as reported by the investigator, type of event as adjudicated by the physician committee, time from first dose of study drug to the event, and time from last dose to the event (if participant has discontinued study drug prior to the event).

4.7. Other Analyses

4.7.1. Subgroup Analyses

Subgroup analyses of the following primary endpoint and secondary endpoints will be made to assess consistency of the intervention effect:

- LARS
- log-transformed NT-proBNP
- LAEDVI
- LAESVI, and
- eGFR

Where the subgroups are defined as follows:

- age: < median vs ≥ median
- sex: female versus male
- race: white, Asian, others
- baseline NYHA class: I-II vs III-IV
- region: North America, Latin America, Asia, Europe and other countries
- atrial fibrillation or atrial flutter on the screening ECG: Yes vs. No
- baseline value of the endpoint: < median vs ≥ median

For each subgroup analysis, the following 2 models will be conducted:

- MMRM model as described in Section 4.1 on the subgroup only.
- Full MMRM model: the MMRM model as described in Section 4.1 adding interactions between subgroup and visit, between subgroup and treatment and between subgroup, treatment and visit as fixed effects.

If the number of participants is too small (less than 10%) within a subgroup, then the subgroup categories may be redefined prior to unblinding the study.

For safety lab variable hemoglobin, subgroup analysis will be done for each sex group (female vs. male) using MMRM model described in Section 4.1.

Additional subgroup analyses may also be performed.

4.8. Interim Analyses

There may be up to 4 interim analyses including primary database lock.

A planned interim analysis may be conducted when between 40% and 80% of the participants complete Week 26 at Visit 24 or discontinue the study. The interim will be for the purpose of internal planning and decision-making and may assess safety, PK, and/or efficacy measures. At the discretion of the sponsor, the prespecified interim analysis may not be conducted. If prespecified interim analysis happens, an AC will be formed to review the interim analyses in an unblinded manner. The details regarding the number of participants and type of analysis will be provided in the AC charter and in the unblinding plan. Information that may unblind the study during the analyses will not be reported to study sites or blinded study team members before the study has been unblinded. Study sites will receive information about interim results only if deemed necessary for the safety of the participants. The study will not be stopped based on the efficacy of LY3540378 versus placebo. Therefore, there will be no inflation of the type 1 error rate, and no need to employ an alpha spending function or multiplicity adjustment.

The primary database lock and primary data analysis for Study EZDB may occur when all participants have completed 26 weeks (Visit 24) of treatment. The final database lock and final data analysis will occur when all randomized participants have completed the study. Participants and investigators will remain blinded until the completion of the study. If there is no primary database lock, the primary analysis will be based on the final database lock.

Early access to the PK and PD data before the interim and primary database locks may be conducted to allow population PK/PD analysis and model development. If applicable, this early access will be detailed in the Unblinding Plan and the Population PK/PD Analysis Plan.

5. Sample Size Determination

The sample size calculation is based on the primary efficacy estimand and its endpoint, change from baseline at Week 26 in LARS.

For participants who joined study before amendment (b), they were randomized 1:1:1:1 to the following intervention groups:

- LY3540378 25 mg SC QW
- LY3540378 50 mg SC QW
- LY3540378 100 mg SC QW, and
- placebo.

For participants who joined study after amendment (b), will be randomized 1:2:2:2 to the above intervention groups.

Up to 456 participants will be randomly assigned to ensure at least 114 participants enrolled in each of 50 mg, 100 mg, and placebo groups. Assuming a 20% dropout rate, this will result in at least 91 completers in each of 50 mg, 100 mg, and placebo group. The number of completers in 25 mg will be approximately 64 to 91, depending on when the amendment (b) is globally implemented.

The evaluation of superiority to placebo will be conducted for LY3540378 doses of 50 and 100 mg and combination of 50 and 100 mg. No adjustment for multiplicity will be performed. Assuming a standard deviation of 8.5%, and a 2-sided, alpha level of 0.05, 91 completers for each treatment arms will provide 88% power to detect a treatment difference of 4% for the primary endpoint for LY3540378 50 mg group versus placebo and LY3540378 100 mg group versus placebo, respectively.

6. Supporting Documentation

6.1. Appendix 1: Demographic and Baseline Characteristics

A listing of participant demographics for all randomized participants will be provided. All demographic and baseline clinical characteristics will be summarized by treatment groups for all randomized participants.

Baseline demographic and clinical characteristics of special interest include but are not limited to:

- demographics: age (years), sex (female, male), race, ethnicity, height (cm), weight (kg), BMI (kg/m^2),
- baseline HF status: NYHA class, atrial fibrillation or flatter at screening
- disease history: history of atrial fibrillation, hypertension, diabetes, myocardial infarction, anemia
- baseline of selected ECHO parameters: LARS, LAEDVI, LAESVI, LA emptying fraction, LVGLS, E/A, E/e’, LVM, LVMI,
- Baseline of selected lab data: NT-proBNP,BNP, BNP, eGFR (creatine- CKD-EPI, mL/min/1.73m^2), eGFR groups (<30, ≥30 to <45, ≥45 to <60, ≥60 to <90, and ≥90 mL/min/1.73 m^2), serum creatine, cystatin-C, UACR, UACR groups of normal(<30) microalbuminuria (≥30 to <300) and macroalbuminuria (≥300), high sensitivity troponin (hs-cTnT)
- Baseline vital Signs: supine systolic and diastolic blood pressure in supine position
- Baseline heart rate
- Baseline treatments: diuretics, RAAS inhibitor, SGLT-2i, Beta blockers, ARNI, and MRA

6.2. Appendix 2: Historical Illnesses and Pre-existing Conditions

The count and percentages of participants with historical illnesses and preexisting conditions will be summarized by treatment groups using the MedDRA PTs nested within SOC. The SOC will be in alphabetical order. Conditions (that is, PTs) will be ordered by decreasing frequency within SOC. This will be summarized for all randomized participants.

6.3. Appendix 3: Treatment Compliance

Listing and summary of prematurely discontinuing study treatment (including discontinuation reason) and discontinuing study will be provided by treatment groups.

If data warrants, the counts and percentages of participants who have dose interruption or have dose de-escalation will be summarized for each treatment group.

Overall treatment compliance will be defined as taking at least 75% of the scheduled LY3540378 or placebo doses. Compliance will be calculated by taking the number of doses administered (regardless of the actual dose in mg administered) divided by the total number of doses expected to be administered, and then multiplied by 100. Overall treatment compliance will be summarized descriptively by treatment group using the full analysis set.

6.4. Appendix 4: Concomitant Medications

Concomitant medications will be summarized by treatment group. The percentages of participants who took concomitant medication will be summarized by treatment using PTs nested within Anatomical Therapeutic Chemical (ATC) Level 3 codes. The concomitant medications will be ordered by decreasing frequency within each ATC level.

6.5. Appendix 5: Important Protocol Deviations

Important protocol deviations are identified in the Trial Issues Management Plan. A listing and summary of important protocol deviations by treatment groups will be provided at the end of study (for all randomized participants).

6.6. Appendix 6: Searching Criteria for Additional Safety Assessments

Hepatic treatment-emergent adverse events

Treatment-emergent, potentially drug-related hepatic disorders will be summarized by treatment using the MedDRA PTs contained in any of the following SMQs:

- Broad and narrow terms in the Liver related investigations, signs and symptoms SMQ (20000008)
- Broad and narrow terms in the Cholestasis and jaundice of hepatic origin SMQ (20000009)
- Broad and narrow terms in the Hepatitis non-infections SMQ (20000010)
- Broad and narrow terms in the Hepatic failure, fibrosis and cirrhosis and other liver damage SMQ (20000013)
- Narrow terms in the Liver-related coagulation and bleeding disturbances SMQ (20000015)
- Narrow PTs in Gallbladder related disorders SMQ (20000124)
- Narrow PTs in Biliary tract disorders SMQ (20000125), and
- Narrow PTs in Gallstone related disorders SMQ (20000127).

Injection site reactions

Treatment emergent injection site reaction will be summarized by treatment using the MedDRA PT in any of the following MedDRA HLTs:

- Injection site reaction
- Administration site reaction, and
- Infusion site reactions.

Vaginal haemorrhage

Vaginal haemorrhage will be summarized by treatment using the MedDRA PT in any of the following MedDRA HLTs:

- Reproductive system haemorrhages
- Vulvovaginal disorders NEC

Breast Tumours

Breast Tumours will be summarized by treatment in any of the following MedDRA HLTs:

- Breast and nipple neoplasms benign
- Breast neoplasms unspecified malignancy
- Breast and nipple neoplasms malignant

Hypotension

Hypotension will be summarized by treatment using the MedDRA PT in MedDRA HLT Vascular hypotensive disorders.

Orthostatic Hypotension

Orthostatic Hypotension will be summarized by treatment the MedDRA PT in any of the following MedDRA HLTs:

- Vascular hypotensive disorders
- Autonomic nervous system disorders

Tissue Changes in the Female Reproductive Tracts

Orthostatic Hypotension will be summarized by treatment in any of the following MedDRA HLTs:

- Cervix neoplasms benign
- Ovarian neoplasms benign
- Reproductive neoplasms female benign NEC

Anaemia

Anaemia will be summarized by treatment using the MedDRA PT in MedDRA HLT Anaemias NEC.

6.7. Appendix 7: The Kansas City Cardiomyopathy Questionnaire Scoring Instructions

1. **Physical Limitation**

Code responses to each of Questions 1a-f as follows:

- Extremely limited = 1
- Quite a bit limited = 2
- Moderately limited = 3
- Slightly limited = 4
- Not at all limited = 5
- Limited for other reasons or did not do = <*missing value*>

If at least three of Questions 1a-f are not missing, then compute

Physical Limitation Score = 100*[(mean of Questions 1a-f actually answered) – 1]/4

Note: If there are n questions in a scale, and the subject must answer m to score the scale, but the subject answers only n-i, where n-i >= m, calculate the mean of those questions as

(sum of the responses to those n-i questions) / (n-i)

not

(sum of the responses to those n-i questions) / n

2. Symptom Stability

Code the response to Question 2 as follows:

- Much worse = 1
- Slightly worse = 2
- Not changed = 3
- Slightly better = 4
- Much better = 5
- I've had no symptoms over the last 2 weeks = 3

If Question 2 is not missing, then compute

Symptom Stability Score = 100*[(Question 2) – 1]/4

3. Symptom Frequency

Code responses to Questions 3, 5, 7 and 9 as follows:

<u>Question 3</u>

- Every morning = 1
- 3 or more times a week but not every day = 2
- 1-2 times a week = 3
- Less than once a week = 4
- Never over the past 2 weeks = 5

<u>Questions 5 and 7</u>

- All of the time = 1
- Several times a day = 2
- At least once a day = 3
- 3 or more times a week but not every day = 4
- 1-2 times a week = 5
- Less than once a week = 6
- Never over the past 2 weeks = 7

<u>Question 9</u>

- Every night = 1
- 3 or more times a week but not every day = 2
- 1-2 times a week = 3
- Less than once a week = 4

- Never over the past 2 weeks = 5

If at least two of Questions 3, 5, 7 and 9 are not missing, then compute:

S3 = [(Question 3) – 1]/4

S5 = [(Question 5) – 1]/6

S7 = [(Question 7) – 1]/6

S9 = [(Question 9) – 1]/4

Symptom Frequency Score = 100*(mean of S3, S5, S7 and S9)

4. Symptom Burden

Code responses to each of Questions 4, 6 and 8 as follows:

- Extremely bothersome = 1
- Quite a bit bothersome = 2
- Moderately bothersome = 3
- Slightly bothersome = 4
- Not at all bothersome = 5
- I’ve had no swelling/fatigue/shortness of breath = 5

If at least one of Questions 4, 6 and 8 is not missing, then compute

Symptom Burden Score = 100*[(mean of Questions 4, 6 and 8 actually answered) – 1]/4

5. Total Symptom Score

Total Symptom Score = mean of the following available summary scores:

- Symptom Frequency Score
- Symptom Burden Score

6. Self-Efficacy

Code responses to Questions 10 and 11 as follows:

Question 10

- Not at all sure = 1
- Not very sure = 2
- Somewhat sure = 3
- Mostly sure = 4
- Completely sure = 5

Question 11

- Do not understand at all = 1
- Do not understand very well = 2
- Somewhat understand = 3
- Mostly understand = 4
- Completely understand = 5

If at least one of Questions 10 and 11 is not missing, then compute

Self-Efficacy Score = 100*[(mean of Questions 10 and 11 actually answered) – 1]/4

7. Quality of Life

Code responses to Questions 12, 13 and 14 as follows:

Question 12

- It has extremely limited my enjoyment of life = 1
- It has limited my enjoyment of life quite a bit = 2
- It has moderately limited my enjoyment of life = 3
- It has slightly limited my enjoyment of life = 4
- It has not limited my enjoyment of life at all = 5

Question 13

- Not at all satisfied = 1
- Mostly dissatisfied = 2
- Somewhat satisfied = 3
- Mostly satisfied = 4
- Completely satisfied = 5

Question 14

- I felt that way all of the time = 1
- I felt that way most of the time = 2
- I occasionally felt that way = 3
- I rarely felt that way = 4
- I never felt that way = 5

If at least one of Questions 12, 13 and 14 is not missing, then compute

Quality of Life Score = 100*[(mean of Questions 12, 13 and 14 actually answered) – 1]/4

8. Social Limitation

Code responses to each of Questions 15a-d as follows:

- Severely limited = 1
- Limited quite a bit = 2
- Moderately limited = 3
- Slightly limited = 4
- Did not limit at all = 5

Does not apply or did not do for other reasons = <*missing value*>

If at least two of Questions 15a-d are not missing, then compute

Social Limitation Score = 100*[(mean of Questions 15a-d actually answered) – 1]/4

9. Overall Summary Score

Overall Summary Score = mean of the following available summary scores:

- Physical Limitation Score
- Total Symptom Score
- Quality of Life Score
- Social Limitation Score

10. Clinical Summary Score

Clinical Summary Score = mean of the following available summary scores:

- Physical Limitation Score
- Total Symptom Score

7. References

Fu H, Manner D. Bayesian adaptive dose-finding studies with delayed responses. *J Biopharm Stat*. 2010;20(5):1055-1070. https://doi.org/10.1080/10543400903315740

Inker LA, Eneanya ND, Coresh J, et al. New Creatinine- and Cystatin C-Based Equations to Estimate GFR without Race. *N Engl J Med*. 2021;385(19):1737-1749. https://doi.org/10.1056/NEJMoa2102953

Title Page

Confidential Information

The information contained in this document is confidential and is intended for the use of clinical investigators. It is the property of Eli Lilly and Company or its subsidiaries and should not be copied by or distributed to persons not involved in the clinical investigation of LY3540378, unless such persons are bound by a confidentiality agreement with Eli Lilly and Company or its subsidiaries.

Note to Regulatory Authorities: This document may contain protected personal data and/or commercially confidential information exempt from public disclosure. Eli Lilly and Company requests consultation regarding release/redaction prior to any public release. In the United States, this document is subject to Freedom of Information Act (FOIA) Exemption 4 and may not be reproduced or otherwise disseminated without the written approval of Eli Lilly and Company or its subsidiaries.

Protocol Title: A Phase 2, Randomized, Double-Blind, Placebo-Controlled Study to Investigate the Efficacy and Safety of LY3540378 in Adults with Worsening Chronic Heart Failure with Preserved Ejection Fraction (HFpEF)

Protocol Number: J3E-MC-EZDB

Amendment Number: b

Compound: LY3540378

Brief Title: Efficacy and Safety of LY3540378 in Adults with Worsening Chronic Heart Failure with Preserved Ejection Fraction

Study Phase: 2

Sponsor Name: Eli Lilly and Company

Legal Registered Address: Indianapolis, Indiana, USA46285

Regulatory Agency Identifier Numbers:

IND: 152593

EudraCT Number: 2022-000780-48

EU CT Number: 2023-505902-40-00

Approval Date: Protocol Amendment (b) Electronically Signed and Approved by Lilly on date provided below.

Document ID: VV-CLIN-119494

Final

Approved on 09_Apr 2024 GMT
Downloaded by Flora Sam on 09 Apr 2024

Medical monitor Name and Contact Information will be provided separately.

Final

Approved on 09_Apr 2024 GMT
Downloaded by Flora Sam on 09 Apr 2024

Protocol Amendment Summary of Changes Table

DOCUMENT HISTORY	
**Document**	**Date**
Amendment a	*01-Jun-2023*
Original Protocol	*07-Jun-2022*

Amendment [b]

The amendment is considered to be substantial because it is likely to have a significant impact on the

- safety or the rights of the study participants, and
- the quality or safety of any investigational medicinal product used in the study.

Overall Rationale for the Amendment:

The overall rationale for this amendment is to

- update the language for the hepatic monitoring criteria
- clarify sample size, and
- better assess the outcomes of the 50 mg and 100 mg study arms.

Section # and Name	Description of Change	Brief Rationale
Throughout	Revised the number of participants from "approximately 432" to "up to 456"	To account for the participant drop off rate that is higher than initially planned
	Revised the randomization ratio to intervention groups from "1:1:1:1" to "1:2:2:2"	To better assess the outcomes of the 50 mg and 100 mg study arms
Synopsis	Study Population subsection:<br>Updated criteria	Updated to align with clarification to inclusion criteria 1, 2, and 3 in Section 5.1.
Synopsis<br>3. Objectives, Endpoints, and Estimands<br>8.1.2. Other Efficacy Assessments	Revised the equation to be used for calculating eGFR from "creatinine and cystatin C" to "CKD-EPI Creatinine-Cystatin equation (2021)"	Clarification
1.3. Schedule of Activities (SoA)	Screening:<br>Added statement that sample collections for the local and central laboratories and the ECG test should all be done on the same day. Other screening procedures, for example, ECHO may be conducted on another day.<br>Telehealth visits:	

Final

Approved on 09 Apr 2024 GMT
Downloaded by Flora Sam on 09 Apr 2024

Section # and Name	Description of Change	Brief Rationale
	Added “or required by local regulations”	
	Visits 1 through 16 table<br>Laboratory Tests and Sample Collections row: Added statement on reinforcing the importance of collecting BNP, NT-proBNP, and eGFR sample for both central and local laboratory testing on the same day	
	Visits 17 through 24, ED, and Follow-Up table<br>Added important note to reiterate that from Visit 17 to Visit 24, site visits are no longer every week, but every 2 weeks. Also, added language that sites should remind participants of the injection duration and on-site visit<br>“Randomization and Dosing” section:<br>• Administer study intervention ~~on a~~ weekly ~~basis (self-administration or site)~~<br>• Updated the notes to administer study intervention	
2.2. Background	Clinical Study EZDA preliminary results subsection:<br>Deleted “an ongoing”	Study EZDA has been completed
5.1. Inclusion Criteria	Criterion 1:<br>Added indication and deleted the word “participants”	Clarification
	Criterion 2 has been deleted	Clarification and requested by investigators
	Criterion 3B:<br>Added “ at that index event”	Clarification
	Criterion 3:<br>Revised the IV requirement of the bolus doses of IV diuretics at that index event from “≥2 bolus doses” to “≥1 bolus dose”	Clarification and requested by investigators
	Criterion 6B:<br>Revised definition of treatment with IV diuretics from “≥2 IV bolus doses” to “≥1 bolus dose”	To align with the revisions made to criterion 3
	Criterion 8:<br>Added unit for measurement of eGFR: mL/min/1.73 m^2	For editorial consistency

Final

Approved on 09 Apr 2024 GMT
Downloaded by Flora Sam on 09 Apr 2024

Section # and Name	Description of Change	Brief Rationale
	Criterion 9:<br>• Revised the criterion to “A” and “B”.<br>• Deleted the note indicating “Do not need to be at a stable dose”	Clarification and requested by investigators
5.2. Exclusion Criteria	Criterion 26: Specified that a participant is considered eligible if subsequently the hemoglobin is ≥10 g/dL before V2 (randomization)	Clarification
	Criterion 38:<br>Added “a documented ALT or AST >5x ULN” to the classification for evidence of hepatic insufficiency	To comply with updated guidance from internal liver safety monitoring committee to enhance monitoring
	New criterion added: 48<br>De novo (first occurrence or diagnosis) HFpEF is not permitted	Clarification and requested by investigators
5.5. Criteria for Temporarily Delaying Enrollment, Randomization, or Administration of Study Intervention of a Participant	Deleted “Randomization, or Administration of Study Intervention” from the section heading	To clarify the criteria applicable for the study
6.3. Measures to Minimize Bias: Randomization and Blinding	Updated section with the revised randomization ratio	To better assess the outcomes of the 50 mg and 100 mg study arms.
6.8. Concomitant Therapy	Added language from the 2022 AHA/ACC/HFSA Guideline, where SGLT-2i is designated a Class 2A indication in decreasing HF hospitalizations and cardiovascular mortality in HFpEF participants	Clarification
7.1.1. Hepatic Criteria for Study Intervention Interruption or Discontinuation	Replaced the liver chemistry stopping criteria: Updated the section heading and added a statement to refer to Section 8.3.5 for the details	To comply with updated guidance from internal liver safety monitoring committee to enhance monitoring
8.3.5. Hepatic Safety Monitoring, Evaluation, and Criteria for Study Intervention Interruption or Discontinuation	Added tables for actions to be taken based on abnormal hepatic laboratory or clinical changes for participants with normal or near-normal baseline and participants with elevated baseline	
8.3.5.1. Close Hepatic Monitoring<br>8.3.5.2. Comprehensive Hepatic Evaluation<br>8.3.5.3. Study Intervention Interruption or Discontinuation	Updated the sections for actions to be taken based on abnormal hepatic laboratory or clinical changes for participants with normal or near-normal baseline and participants with elevated baseline	

Final

Approved on 09 Apr 2024 GMT
Downloaded by Flora Sam on 09 Apr 2024

Section # and Name	Description of Change	Brief Rationale
9.5. Sample Size Determination	Updated section with the revised randomization ratio	To better assess the outcomes of the 50 mg and 100 mg study arms.
10.6.1. Hepatic Evaluation Testing	Updated hepatic evaluation testing table	To clarify the hepatic monitoring guidance
10.7. Appendix 7: Provisions for Changes in Study Conduct During Exceptional Circumstances	Added a flow diagram illustrating screening and randomization	For ease of interpretation
10.9. Appendix 9: Protocol Amendment History (Newly added section)	Inserted summary of amendment (a) rationale and changes	To update the amendment history
Throughout the protocol	Minor formatting and editorial changes	Minor, therefore, not detailed

Final

Approved on 09 Apr 2024 GMT
Downloaded by Flora Sam on 09 Apr 2024

Table of Contents

1. Protocol Summary 10
1.1. Synopsis 10
1.2. Schema 14
1.3. Schedule of Activities (SoA) 15

2. Introduction 38
2.1. Study Rationale 38
2.2. Background 38
2.3. Benefit/Risk Assessment 40
2.3.1. Risk Assessment 40
2.3.2. Overall Benefit Risk Conclusion 41

3. Objectives, Endpoints, and Estimands 42

4. Study Design 45
4.1. Overall Design 45
4.2. Scientific Rationale for Study Design 46
4.3. Justification for Dose 46
4.4. End of Study Definition 47

5. Study Population 48
5.1. Inclusion Criteria 48
5.2. Exclusion Criteria 50
5.3. Lifestyle Considerations 52
5.4. Screen Failures 52
5.5. Criteria for Temporarily Delaying Enrollment of a Participant 53

6. Study Interventions and Concomitant Therapy 54
6.1. Study Interventions Administered 54
6.2. Preparation, Handling, Storage, and Accountability 55
6.3. Measures to Minimize Bias: Randomization and Blinding 55
6.4. Study Intervention Compliance 56
6.5. Dose Modification 57
6.6. Continued Access to Study Intervention after the End of the Study 57
6.7. Treatment of Overdose 57
6.8. Concomitant Therapy 58

7. Discontinuation of Study Intervention and Participant Discontinuation/Withdrawal 59
7.1. Discontinuation of Study Intervention 59
7.1.1. Hepatic Criteria for Study Intervention Interruption or Discontinuation 59
7.1.2. QTc Stopping Criteria 59
7.1.3. Hypersensitivity Reactions 59
7.1.4. Temporary Discontinuation 59
7.2. Participant Discontinuation/Withdrawal from the Study 60
7.3. Lost to Follow Up 61

Final

Approved on 09 Apr 2024 GMT
Downloaded by Flora Sam on 09 Apr 2024

8. Study Assessments and Procedures 62
8.1. Efficacy Assessments 62
8.1.1. Primary Efficacy Assessment 62
8.1.2. Other Efficacy Assessments 62
8.2. Safety Assessments 66
8.2.1. Physical Examinations 66
8.2.2. Vital Signs 66
8.2.3. Electrocardiograms 66
8.2.4. Clinical Safety Laboratory Tests 67
8.2.5. Pregnancy Testing 67
8.3. Adverse Events, Serious Adverse Events, and Product Complaints 68
8.3.1. Timing and Mechanism for Collecting Events 69
8.3.2. Pregnancy 70
8.3.3. Injection-Site Reactions 71
8.3.4. Hypersensitivity Reactions 71
8.3.5. Hepatic Safety Monitoring, Evaluation, and Criteria for Study Intervention Interruption or Discontinuation 72
8.3.6. Major Adverse Cardiovascular Events (MACE) 76
8.3.7. Safety Topics of Special Interest 77
8.4. Pharmacokinetics 77
8.4.1. Bioanalysis 78
8.5. Pharmacodynamics 78
8.6. Genetics 78
8.7. Biomarkers 78
8.8. Immunogenicity Assessments 79
8.9. Medical Resource Utilization and Health Economics 79

9. Statistical Considerations 80
9.1. Statistical Hypotheses 80
9.1.1. Multiplicity Adjustment 80
9.2. Analyses Sets 80
9.3. Statistical Analyses 80
9.3.1. General Considerations 80
9.3.2. Primary Endpoint(s)/Estimand(s) Analysis 82
9.3.3. Secondary Endpoints Analysis 82
9.3.4. Tertiary Endpoints Analysis 82
9.3.5. Safety Analyses 82
9.3.6. Pharmacokinetic and Pharmacodynamic Analyses 83
9.3.7. Immunogenicity Assessments 83
9.3.8. Subgroup Analyses 83
9.4. Interim Analysis 84
9.5. Sample Size Determination 84

10. Supporting Documentation and Operational Considerations 86
10.1. Appendix 1: Regulatory, Ethical, and Study Oversight Considerations 86
10.1.1. Regulatory and Ethical Considerations 86

Final

Approved on 09 Apr 2024 GMT
Downloaded by Flora Sam on 09 Apr 2024

10.1.2. Financial Disclosure 86
10.1.3. Informed Consent Process 87
10.1.4. Data Protection 87
10.1.5. Committees Structure 88
10.1.6. Dissemination of Clinical Study Data 88
10.1.7. Data Quality Assurance 88
10.1.8. Source Documents 90
10.1.9. Study and Site Start and Closure 90
10.1.10. Publication Policy 91
10.1.11. Investigator Information 91
10.1.12. Sample Retention 91
10.2. Appendix 2: Clinical Laboratory Tests 92
10.2.1. Laboratory Samples Obtained at the Time of a Systemic Hypersensitivity Event 96
10.3. Appendix 3: Adverse Events and Serious Adverse Events: Definitions and Procedures for Recording, Evaluating, Follow-up, and Reporting 97
10.3.1. Definition of AE 97
10.3.2. Definition of SAE 98
10.3.3. Definition of Product Complaints 99
10.3.4. Recording and Follow-Up of AE and/or SAE and Product Complaints 99
10.3.5. Reporting of SAEs 101
10.3.6. Regulatory Reporting Requirements 101
10.4. Appendix 4: Contraceptive and Barrier Guidance 103
10.4.1. Definitions 103
10.4.2. Contraception Guidance 104
10.5. Appendix 5: Genetics 105
10.6. Appendix 6: Liver Safety: Suggested Actions and Follow-up Assessments 106
10.6.1. Hepatic Evaluation Testing 106
10.7. Appendix 8: Abbreviations and Definitions 112
10.9. Appendix 9: Protocol Amendment History 117

11. References 121

Final

Approved on 09 Apr 2024 GMT
Downloaded by Flora Sam on 09 Apr 2024

1. Protocol Summary

1.1. Synopsis

Protocol Title: A Phase 2, Randomized, Double-Blind, Placebo-Controlled Study to Investigate the Efficacy and Safety of LY3540378 in Adults with Worsening Chronic Heart Failure with Preserved Ejection Fraction (HFpEF)

Brief Title: Efficacy and Safety of LY3540378 in Adults with Worsening Chronic Heart Failure with Preserved Ejection Fraction

Regulatory Agency Identifier Numbers:

IND: 152593
EudraCT Number: 2022-000780-48
EU CT Number: 2023-505902-40-00

Rationale:

Heart failure with preserved ejection fraction (HFpEF) is a heterogenous clinical syndrome complicated by a high prevalence of co-existing comorbidities with no known single pathophysiological process. Until the publication of the EMPEROR-Preserved trial (empagliflozin in HFpEF) in October 2021, no clinical trial had shown benefit of an experimental therapy in HFpEF. Empagliflozin has been approved for treatment for a wide range of patients with heart failure (HF), and sacubitril/valsartan was granted an expanded label for HF in adult patients with chronic HF particularly those with LVEF below normal.

Study J3E-MC-EZDB (EZDB) will investigate the effects of 26-week treatment with LY3540378 on left atrial reservoir strain (LARS) in participants with worsening chronic HFpEF. This is the first Phase 2 study, and data from this study will inform dose decisions for the clinical development of LY3540378 for HFpEF.

Final

Approved on 09_Apr 2024 GMT
Downloaded by Flora Sam on 09 Apr 2024

Objectives, Endpoints, and Estimands:

Objectives	Endpoints
Primary	
To demonstrate that LY3540378 administered SC QW is superior to placebo for improving atrial myopathy in participants with worsening chronic HFpEF	Change from baseline to Week 26 in LARS
Secondary	
To compare the effect of LY3540378 administered SC QW on participants with worsening chronic HFpEF	Change from baseline to Weeks 12 and 26 in<br>• Log-transformed NT-proBNP<br>• LAEDVI<br>• LAESVI<br>• eGFR (CKD-EPI Creatinine-Cystatin equation [2021])<br>• serum creatinine, and<br>• cystatin-C
To assess safety and tolerability of LY3540378 administered SC QW	• AE overall<br>• safety topics of special interest

Abbreviations: AE = adverse event; CKD-EPI = Chronic Kidney Disease-Epidemiology Collaboration; ECG = electrocardiogram; eGFR = estimated glomerular filtration rate; HFpEF = heart failure with preserved ejection fraction; LAEDVI = left atrial end-diastolic volume index; LAESVI = left atrial end-systolic volume index; LARS = left atrial reservoir strain; NT-proBNP = N-terminal pro-B-type natriuretic peptide; SC = subcutaneous; QW = weekly.

Final

Overall Design:

Study EZDB is a Phase 2, multicenter, randomized, double-blind, placebo-controlled study that will investigate the effects of treatment with LY3540378 compared with placebo on participants with worsening chronic HFpEF.

Brief Summary:

Study EZDB will investigate the hypothesis that LY3540378 administered subcutaneously (SC) weekly is superior to placebo for improvement of atrial myopathy in participants with worsening chronic HFpEF at Week 26.

Participants with worsening chronic HFpEF will be treated with LY3540378 or placebo for 26 weeks. Visits are scheduled every 1 to 2 weeks.

The maximum total duration of study participation for each participant, including screening and safety follow-up periods, is approximately 32 weeks, across the following study periods:

- Screening: up to 14 days
- Double-Blind Treatment: 26 weeks, and
- Safety Follow-Up: 4 weeks.

Approved on 09 Apr 2024 GMT
Downloaded by Flora Sam on 09 Apr 2024

Study Population:

In general, an individual may take part in the study if they

- Have a known clinical diagnosis of HFpEF or "diastolic heart failure" and are at least 18 years of age or the legal age of consent in the jurisdiction in which the study is taking place at the time of signing the informed consent.
- Have experienced an index event, defined as a recent hospitalization for HF requiring ≥1 bolus dose of IV diuretics or an out-of-hospital encounter (for example, Emergency Room, clinic visit, infusion clinic) for HF requiring ≥1 bolus dose of IV diuretics at that index event. A single continuous infusion is also allowed.
- Males and females will be eligible for this study.
- Are reliable and willing to make themselves available for the duration of the study and are willing to follow study procedures as required.

Opportunities to study LY3540378 in WHF with HFpEF

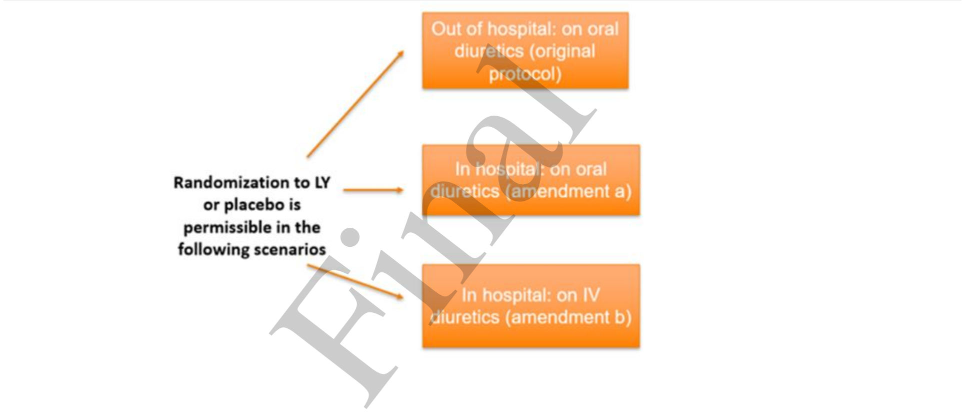

Final

Abbreviations: HFpEF = heart failure with preserved ejection fraction; IV = intravenous; WHF = worsening heart failure.

In general, an individual may not take part in the study if they

- Have any of the following cardiovascular conditions
  - LVEF ≤45% in the past 12 months
  - acute coronary syndrome or percutaneous coronary intervention, coronary artery bypass graft, cardiac mechanical support implantation, within 3 months prior to V2 (randomization), or any other cardiac surgery planned during the study
  - LVAD or cardiac transplantation or have cardiac transplantation planned during the study
  - hypertrophic cardiomyopathy (obstructive or nonobstructive), restrictive cardiomyopathy, active myocarditis, constrictive pericarditis, cardiac sarcoidosis, known amyloid cardiomyopathy, or inherited cardiomyopathy
  - uncorrected cyanotic cardiac disease affecting LV function
  - severe uncorrected valvular disease

Approved on 09 Apr 2024 GMT
Downloaded by Flora Sam on 09 Apr 2024

- Are hospitalized for worsening HF event or received treatment for an urgent HF visit outside of being hospitalized with worsening heart failure (WHF), after V1 (screening) and before V2 (randomization)

Number of Participants:

Up to 456 participants will be randomly assigned to study intervention.

Intervention Groups and Duration:

Participants will be randomized 1:2:2:2 to the following intervention groups:

- LY3540378 25 mg SC QW
- LY3540378 50 mg SC QW
- LY3540378 100 mg SC QW
- Placebo

Intervention administration is by subcutaneous injection, and dosing will occur every week.

Ethical Considerations of Benefit/Risk:

Considering the measures taken to minimize risk for the participants in this study, the potential risks identified in association with LY3540378 are justified by the anticipated benefits that may be afforded to participants with worsening chronic HFpEF.

Data Monitoring Committee: No

Final

Approved on 09 Apr 2024 GMT
Downloaded by Flora Sam on 09 Apr 2024

1.2. Schema

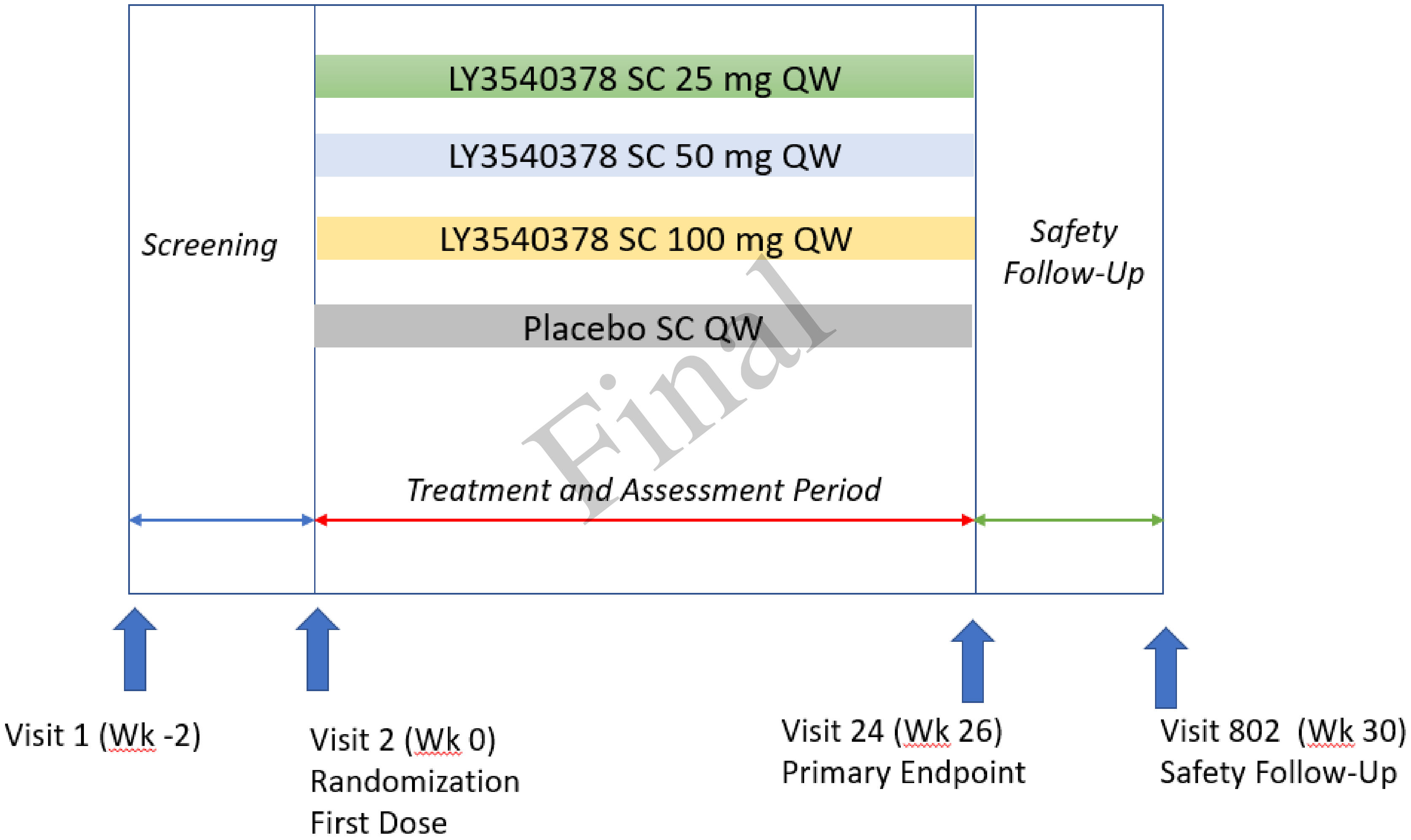

Final
Approved on 09 Apr 2024 GMT
Downloaded by Flora Sam on 09 Apr 2024

1.3. Schedule of Activities (SoA)

Screening

Screening procedures may be conducted over more than 1 day, as long as all procedures are completed within the screening period. However, it is important to note that sample collections for the local and central laboratories and the ECG test should all be done on the same day. Other screening procedures, for example, ECHO may be conducted on another day.

Telehealth visits

Telehealth visits may be by telephone (or other technology), or on site if requested by the participant or required by local regulations.

Unscheduled visits

Unscheduled visits (UV) may occur as needed. The SoA reflects some of the procedures that may occur during these visits. Additional procedures may be performed per investigator discretion.

Final

Approved on 09 Apr 2024 GMT
Downloaded by Flora Sam on 09 Apr 2024

Visits 1 through 16

	SP I – Screening	SP II – Treatment															Comments
Visit number	1	2	3	4	5	6	7	8	9	10	11	12	13	14	15	16	
Weeks from randomization	≤2	0	1	2	3	[a]	4	5	6	7	[b]	8	9	10	11	12	[a]V6 occurs 24-96 hr after the V5 dose<br>[b]V11 occurs 24-96 hr after the V10 dose
Visit interval tolerance (days)	-	-	±2	±2	±2	-	±2	±2	±2	±2	-	±2	±2	±2	±2	±2	
Office (O)/telehealth (T)	O	O	O	O	O	O	T	T	T	O	O	O	T	O	T	O	
Informed consent	X																The informed consent form must be signed before any protocol-specific tests or procedures are performed.<br>See Section 10.1.3 for additional details.
Inclusion and exclusion criteria, review and confirm	X	X															Inclusion or exclusion criteria should be confirmed prior to drug assignment and administration of first dose of study intervention.

Final

Approved on 09_Apr 2024 GMT
Downloaded by Flora Sam on 09 Apr 2024

	SP I – Screening	SP II – Treatment															Comments
Visit number	1	2	3	4	5	6	7	8	9	10	11	12	13	14	15	16	
Weeks from randomization	≤2	0	1	2	3	[a]	4	5	6	7	[b]	8	9	10	11	12	[a]V6 occurs 24-96 hr after the V5 dose<br>[b]V11 occurs 24-96 hr after the V10 dose
Visit interval tolerance (days)	-	-	±2	±2	±2	-	±2	±2	±2	±2	-	±2	±2	±2	±2	±2	
Office (O)/telehealth (T)	O	O	O	O	O	O	T	T	T	O	O	O	T	O	T	O	
Demographics	X																Includes ethnicity (where permissible), sex, and race.
Preexisting conditions and medical history, including relevant surgical history	X																All conditions ongoing and relevant past surgical and medical history should be collected.
Prespecified medical history (indication and history of interest)	X																Includes HF history, hospitalizations for HF, CVD, MI, atrial fibrillation or atrial flutter, stroke.
Prior treatments for indication	X																
Substance use (alcohol, caffeine,	X																

Final

Approved on 09_Apr 2024 GMT
Downloaded by Flora Sam on 09 Apr 2024

	SP I – Screening	SP II – Treatment															Comments
Visit number	1	2	3	4	5	6	7	8	9	10	11	12	13	14	15	16	
Weeks from randomization	≤2	0	1	2	3	[a]	4	5	6	7	[b]	8	9	10	11	12	[a]V6 occurs 24-96 hr after the V5 dose<br>[b]V11 occurs 24-96 hr after the V10 dose
Visit interval tolerance (days)	-	-	±2	±2	±2	-	±2	±2	±2	±2	-	±2	±2	±2	±2	±2	
Office (O)/telehealth (T)	O	O	O	O	O	O	T	T	T	O	O	O	T	O	T	O	
tobacco use)																	
Concomitant medications	X	X	X	X	X	X	X	X	X	X	X	X	X	X	X	X	
AEs	X	X	X	X	X	X	X	X	X	X	X	X	X	X	X	X	AE collection begins when the ICF is signed (Section 8.3.1). For safety topics of special interest, additional data could be collected (Section 8.3.7).
Physical Evaluation																	
Height	X																
Weight	X	X	X	X	X	X				X	X	X		X		X	
Vital signs	X	X	X	X	X	X				X	X	X		X		X	Includes BP (supine and standing), pulse

Final

Approved on 09_Apr 2024 GMT
Downloaded by Flora Sam on 09 Apr 2024

	SP I – Screening	SP II – Treatment															Comments
Visit number	1	2	3	4	5	6	7	8	9	10	11	12	13	14	15	16	
Weeks from randomization	≤2	0	1	2	3	[a]	4	5	6	7	[b]	8	9	10	11	12	[a]V6 occurs 24-96 hr after the V5 dose<br>[b]V11 occurs 24-96 hr after the V10 dose
Visit interval tolerance (days)	-	-	±2	±2	±2	-	±2	±2	±2	±2	-	±2	±2	±2	±2	±2	
Office (O)/telehealth (T)	O	O	O	O	O	O	T	T	T	O	O	O	T	O	T	O	
																	rate, respiratory rate, and temperature. Vital signs should be taken before ECG tracing and collection of blood samples and measured after participant has been supine at least 5 min. Additional vital signs may be measured as necessary at investigator discretion.<br>BP and PR to be taken in triplicate at V2, V5, V6, and V16.<br>See Section 8.2.2.

Final

Approved on 09_Apr 2024 GMT
Downloaded by Flora Sam on 09 Apr 2024

	SP I – Screening	SP II – Treatment															Comments
Visit number	1	2	3	4	5	6	7	8	9	10	11	12	13	14	15	16	
Weeks from randomization	≤2	0	1	2	3	[a]	4	5	6	7	[b]	8	9	10	11	12	[a]V6 occurs 24-96 hr after the V5 dose<br>[b]V11 occurs 24-96 hr after the V10 dose
Visit interval tolerance (days)	-	-	±2	±2	±2	-	±2	±2	±2	±2	-	±2	±2	±2	±2	±2	
Office (O)/telehealth (T)	O	O	O	O	O	O	T	T	T	O	O	O	T	O	T	O	
Physical examination	X	X	X	X	X	X				X	X	X		X		X	Includes signs and symptoms of HF and NYHA classification. See Section 8.2.1 for additional information.
12-lead ECG	X	X			X	X										X	Collect ECG prior to collection of any blood samples.<br>ECG at V1 is single.<br>ECG at V2 is triplicate, predose.<br>ECG at V5 is triplicate, predose.<br>ECG at V6 is triplicate, performed 24 to 96 hr after the V5

Final

Approved on 09_Apr 2024 GMT
Downloaded by Flora Sam on 09 Apr 2024

	SP I – Screening	SP II – Treatment															Comments
Visit number	1	2	3	4	5	6	7	8	9	10	11	12	13	14	15	16	
Weeks from randomization	≤2	0	1	2	3	[a]	4	5	6	7	[b]	8	9	10	11	12	[a]V6 occurs 24-96 hr after the V5 dose<br>[b]V11 occurs 24-96 hr after the V10 dose
Visit interval tolerance (days)	-	-	±2	±2	±2	-	±2	±2	±2	±2	-	±2	±2	±2	±2	±2	
Office (O)/telehealth (T)	O	O	O	O	O	O	T	T	T	O	O	O	T	O	T	O	
																	dose.<br>ECG at V16 is triplicate, predose.<br>ECGs may be repeated at the investigator's discretion at any visit.
Echocardiogram	X															X	
Patient-Reported Outcomes (Electronic)																	Complete prior to any clinical-administered assessments.
Most bothersome symptom	X	X		X								X		X		X	
Dyspnea NRS	X	X		X								X		X		X	
Edema NRS	X	X		X								X		X		X	

Final

Approved on 09_Apr 2024 GMT
Downloaded by Flora Sam on 09 Apr 2024

	SP I – Screening	SP II – Treatment															Comments
Visit number	1	2	3	4	5	6	7	8	9	10	11	12	13	14	15	16	
Weeks from randomization	≤2	0	1	2	3	[a]	4	5	6	7	[b]	8	9	10	11	12	[a]V6 occurs 24-96 hr after the V5 dose<br>[b]V11 occurs 24-96 hr after the V10 dose
Visit interval tolerance (days)	-	-	±2	±2	±2	-	±2	±2	±2	±2	-	±2	±2	±2	±2	±2	
Office (O)/telehealth (T)	O	O	O	O	O	O	T	T	T	O	O	O	T	O	T	O	
Fatigue NRS	X	X		X								X		X		X	
Patient Global Impression of Status – Heart Failure Overall Health (PGIS-HF Overall Health)	X	X		X								X		X		X	
Patient Global Impression of Change – Heart Failure Overall Health (PGIC-HF Overall Health)				X								X		X		X	
Patient Global Impression of Severity – Heart Failure Symptom Severity (PGIS-HF Symptom Severity)	X	X		X								X		X		X	

Final

Approved on 09_Apr 2024 GMT
Downloaded by Flora Sam on 09 Apr 2024

	SP I – Screening	SP II – Treatment															Comments
Visit number	1	2	3	4	5	6	7	8	9	10	11	12	13	14	15	16	
Weeks from randomization	≤2	0	1	2	3	[a]	4	5	6	7	[b]	8	9	10	11	12	[a]V6 occurs 24-96 hr after the V5 dose<br>[b]V11 occurs 24-96 hr after the V10 dose
Visit interval tolerance (days)	-	-	±2	±2	±2	-	±2	±2	±2	±2	-	±2	±2	±2	±2	±2	
Office (O)/telehealth (T)	O	O	O	O	O	O	T	T	T	O	O	O	T	O	T	O	
Patient Global Impression of Change – Heart Failure Symptom Severity (PGIC-HF Symptom Severity)				X								X		X		X	
Kansas City Cardiomyopathy Questionnaire (KCCQ)		X														X	
Laboratory Tests and Sample Collections																	
Hematology	X	X	X		X					X				X		X	
Clinical chemistry	X	X	X		X					X				X		X	
Lipid panel		X														X	
Urinalysis		X	X													X	
Urine chemistry		X	X		X					X				X		X	

Final

Approved on 09 Apr 2024 GMT
Downloaded by Flora Sam on 09 Apr 2024

	SP I – Screening	SP II – Treatment															Comments
Visit number	1	2	3	4	5	6	7	8	9	10	11	12	13	14	15	16	
Weeks from randomization	≤2	0	1	2	3	[a]	4	5	6	7	[b]	8	9	10	11	12	[a]V6 occurs 24-96 hr after the V5 dose<br>[b]V11 occurs 24-96 hr after the V10 dose
Visit interval tolerance (days)	-	-	±2	±2	±2	-	±2	±2	±2	±2	-	±2	±2	±2	±2	±2	
Office (O)/telehealth (T)	O	O	O	O	O	O	T	T	T	O	O	O	T	O	T	O	
Follicle-stimulating hormone (FSH)	X																Perform if needed to confirm postmenopausal status. Definition in Section 10.4.1.
N-terminal pro-B-type natriuretic peptide (NT-proBNP)	X	X	X		X					X				X		X	At screening (V1), it is important that the central and local laboratory tests be collected on the same day. Central and local laboratory tests must be drawn ≤24 hours. At screening, result will be generated locally for inclusion and by Lilly-designated
Brain natriuretic peptide (BNP)	X	X	X		X					X				X		X	

Final

Approved on 09_Apr 2024 GMT
Downloaded by Flora Sam on 09 Apr 2024

	SP I – Screening	SP II – Treatment															Comments
Visit number	1	2	3	4	5	6	7	8	9	10	11	12	13	14	15	16	
Weeks from randomization	≤2	0	1	2	3	[a]	4	5	6	7	[b]	8	9	10	11	12	[a]V6 occurs 24-96 hr after the V5 dose<br>[b]V11 occurs 24-96 hr after the V10 dose
Visit interval tolerance (days)	-	-	±2	±2	±2	-	±2	±2	±2	±2	-	±2	±2	±2	±2	±2	
Office (O)/telehealth (T)	O	O	O	O	O	O	T	T	T	O	O	O	T	O	T	O	
																	laboratory for statistical data evaluation.<br>For all other visits, results will be generated by Lilly-designated laboratory.
Cystatin-C	X	X	X		X					X				X		X	
Troponin, high sensitivity (hs-cTnT)		X	X		X					X				X		X	
C-reactive protein, high sensitivity (hsCRP)		X														X	
Urinary albumin to creatinine ratio (UACR)		X	X		X					X				X		X	
Estimated glomerular filtration rate	X	X	X		X					X				X		X	At screening (V1), it is important that the

Final

Approved on 09_Apr 2024 GMT
Downloaded by Flora Sam on 09 Apr 2024

	SP I – Screening	SP II – Treatment															Comments
Visit number	1	2	3	4	5	6	7	8	9	10	11	12	13	14	15	16	
Weeks from randomization	≤2	0	1	2	3	a	4	5	6	7	b	8	9	10	11	12	[a]V6 occurs 24-96 hr after the V5 dose [b]V11 occurs 24-96 hr after the V10 dose
Visit interval tolerance (days)	-	-	±2	±2	±2	-	±2	±2	±2	±2	-	±2	±2	±2	±2	±2	
Office (O)/telehealth (T)	O	O	O	O	O	O	T	T	T	O	O	O	T	O	T	O	
(eGFR)																	central and local laboratory tests be collected on the same day. Central and local laboratory tests must be drawn ≤24 hours. Calculated using Chronic Kidney Disease Epidemiology Collaboration (CKD-EPI) method. At screening, result will be generated locally using creatinine for inclusion and by Lilly-designated laboratory for

Final

Approved on 09_Apr 2024 GMT
Downloaded by Flora Sam on 09 Apr 2024

	SP I – Screening	SP II – Treatment															Comments
Visit number	1	2	3	4	5	6	7	8	9	10	11	12	13	14	15	16	
Weeks from randomization	≤2	0	1	2	3	[a]	4	5	6	7	[b]	8	9	10	11	12	[a]V6 occurs 24-96 hr after the V5 dose<br>[b]V11 occurs 24-96 hr after the V10 dose
Visit interval tolerance (days)	-	-	±2	±2	±2	-	±2	±2	±2	±2	-	±2	±2	±2	±2	±2	
Office (O)/telehealth (T)	O	O	O	O	O	O	T	T	T	O	O	O	T	O	T	O	
																	statistical data evaluation.<br>For all other visits, results will be generated by Lilly-designated laboratory.
Hemoglobin A1c (HbA1c)		X															
Human leukocyte antigen (HLA)		X															
Dehydroepiandrosterone (DHEA)		X															
Androstenedione		X															
Pharmacokinetic (PK) samples		X	X		X	X				X	X			X		X	Collect vital signs prior to PK sample.<br>See Pharmacokinetic Sampling Schedule below the SoA for

Final

Approved on 09_Apr 2024 GMT
Downloaded by Flora Sam on 09 Apr 2024

	SP I – Screening	SP II – Treatment															Comments
Visit number	1	2	3	4	5	6	7	8	9	10	11	12	13	14	15	16	
Weeks from randomization	≤2	0	1	2	3	[a]	4	5	6	7	[b]	8	9	10	11	12	[a]V6 occurs 24-96 hr after the V5 dose<br>[b]V11 occurs 24-96 hr after the V10 dose
Visit interval tolerance (days)	-	-	±2	±2	±2	-	±2	±2	±2	±2	-	±2	±2	±2	±2	±2	
Office (O)/telehealth (T)	O	O	O	O	O	O	T	T	T	O	O	O	T	O	T	O	
																	instructions.
Immunogenicity/ ADA samples		X			X											X	Collect samples before dosing. If an immediate or nonimmediate systemic drug hypersensitivity reaction occurs, collect additional unscheduled samples as detailed in Section 8.3.4.
Stored Samples																	
Genetics sample		X															Sample is optional and can be obtained at or after the specified visit.
Exploratory biomarker sample		X	X		X					X				X		X	Collect biomarkers at V2

Final

Approved on 09_Apr 2024 GMT
Downloaded by Flora Sam on 09 Apr 2024

	SP I – Screening	SP II – Treatment															Comments
Visit number	1	2	3	4	5	6	7	8	9	10	11	12	13	14	15	16	
Weeks from randomization	≤2	0	1	2	3	[a]	4	5	6	7	[b]	8	9	10	11	12	[a]V6 occurs 24-96 hr after the V5 dose<br>[b]V11 occurs 24-96 hr after the V10 dose
Visit interval tolerance (days)	-	-	±2	±2	±2	-	±2	±2	±2	±2	-	±2	±2	±2	±2	±2	
Office (O)/telehealth (T)	O	O	O	O	O	O	T	T	T	O	O	O	T	O	T	O	
																	prior to procedures.
Exploratory biomarker sample (urine)		X														X	
Participant Diary																	
Diary dispensed					X												
Diary review										X							
Randomization and Dosing																	
Register visit with IWRS	X	X	X	X	X	X				X	X	X	X	X	X	X	
Randomization via IWRS		X															
Dispense study intervention via IWRS		X	X	X	X					X		X		X		X	
Administer study intervention on site		X	X	X	X					X		X		X		X	V7, V8, V9, V13 and V15: participants may self-administer

Final

Approved on 09_Apr 2024 GMT
Downloaded by Flora Sam on 09 Apr 2024

	SP I – Screening	SP II – Treatment															Comments
Visit number	1	2	3	4	5	6	7	8	9	10	11	12	13	14	15	16	
Weeks from randomization	≤2	0	1	2	3	[a]	4	5	6	7	[b]	8	9	10	11	12	[a]V6 occurs 24-96 hr after the V5 dose<br>[b]V11 occurs 24-96 hr after the V10 dose
Visit interval tolerance (days)	-	-	±2	±2	±2	-	±2	±2	±2	±2	-	±2	±2	±2	±2	±2	
Office (O)/telehealth (T)	O	O	O	O	O	O	T	T	T	O	O	O	T	O	T	O	
																	injection at home or have injection done at site.
Train participants and/or caregiver in study intervention administration		X	X	X	X					X		X					
Participant returns unused study intervention														X		X	

Final

Approved on 09 Apr 2024 GMT
Downloaded by Flora Sam on 09 Apr 2024

Visits 17 through 24, ED, and Follow-Up

Visit number	17	18	19	20	21	22	23	24	ED	UV	801	802	
Weeks from randomization	14	16	18	20	22	24	25	26	—		28	30	**Important Note**: From Visit 17 to Visit 24, site visits are no longer every week, but every 2 weeks. Sites should remind participants to continue their injection on a weekly basis but attend the onsite visit every 2 weeks.
Visit interval tolerance (days)	±2	±2	±2	±2	±2	±2	±2	±2	—		±3	±3	
Office (O)/Telehealth (T)	T	O	T	O	T	O	T	O	O	O	T	O	
Concomitant medications	X	X	X	X	X	X	X	X	X	X	X	X	
AEs	X	X	X	X	X	X	X	X	X	X	X	X	AE collection begins when the ICF is signed (Section 8.3.1). For safety topics of interest, additional data could be collected (Section 8.3.7).
Physical Evaluation													
Weight		X		X		X		X	X	X		X	
Vital signs		X		X		X		X	X	X		X	Includes BP (supine and standing), pulse rate, respiratory rate, and temperature. Vital signs should be taken before ECG tracing and collection of blood samples and measured after participant has been supine at least 5 min. Additional vital signs may be measured as necessary at investigator discretion.<br>BP and PR to be taken in triplicate at V18 (predose), V24, and ED.<br>See Section 8.2.2.

Final

Approved on 09_Apr 2024 GMT
Downloaded by Flora Sam on 09 Apr 2024

Visit number	17	18	19	20	21	22	23	24	ED	UV	801	802	
Weeks from randomization	14	16	18	20	22	24	25	26	—		28	30	**Important Note**: From Visit 17 to Visit 24, site visits are no longer every week, but every 2 weeks. Sites should remind participants to continue their injection on a weekly basis but attend the onsite visit every 2 weeks.
Visit interval tolerance (days)	±2	±2	±2	±2	±2	±2	±2	±2	—		±3	±3	
Office (O)/Telehealth (T)	T	O	T	O	T	O	T	O	O	O	T	O	
Physical examination		X		X		X		X	X	X		X	Includes signs and symptoms of HF, and NYHA classification.<br>See Section 8.2.1 for additional information.
12-lead ECG		X						X	X	X			Collect ECG prior to collection of any blood samples. Collect ECG at UV if medically indicated at PI discretion.<br>ECG at V18 (predose), V24, and ED is triplicate.<br>ECGs may be repeated at the investigator's discretion at any visit.
Echocardiogram								X	X				
Patient-Reported Outcomes (electronic)													Complete prior to any clinical-administered assessments.
Most bothersome symptom		X		X		X		X	X				
Dyspnea NRS		X		X		X		X	X				
Edema NRS		X		X		X		X	X				
Fatigue NRS		X		X		X		X	X				
Patient Global Impression of Status – Heart Failure Overall Health (PGIS-HF Overall Health)		X		X		X		X	X				

Final

Approved on 09 Apr 2024 GMT
Downloaded by Flora Sam on 09 Apr 2024

Visit number	17	18	19	20	21	22	23	24	ED	UV	801	802	
Weeks from randomization	14	16	18	20	22	24	25	26	—		28	30	**Important Note**: From Visit 17 to Visit 24, site visits are no longer every week, but every 2 weeks. Sites should remind participants to continue their injection on a weekly basis but attend the onsite visit every 2 weeks.
Visit interval tolerance (days)	±2	±2	±2	±2	±2	±2	±2	±2	—		±3	±3	
Office (O)/Telehealth (T)	T	O	T	O	T	O	T	O	O	O	T	O	
Patient Global Impression of Change – Heart Failure Overall Health (PGIC-HF Overall Health)		X		X		X		X	X				
Patient Global Impression of Severity – Heart Failure Symptom Severity (PGIS-HF Symptom Severity)		X		X		X		X	X				
Patient Global Impression of Change – Heart Failure Symptom Severity (PGIC-HF Symptom Severity)		X		X		X		X	X				
Kansas City Cardiomyopathy Questionnaire (KCCQ)								X	X				
Laboratory Tests and Sample Collections													
Hematology		X		X		X		X	X	X			
Clinical chemistry		X		X		X		X	X	X		X	
Lipid panel								X	X	X			
Urinalysis								X	X	X			
Urine chemistry		X		X		X		X	X	X		X	

Final

Approved on 09 Apr 2024 GMT
Downloaded by Flora Sam on 09 Apr 2024

Visit number	17	18	19	20	21	22	23	24	ED	UV	801	802	
Weeks from randomization	14	16	18	20	22	24	25	26	—		28	30	**Important Note**: From Visit 17 to Visit 24, site visits are no longer every week, but every 2 weeks. Sites should remind participants to continue their injection on a weekly basis but attend the onsite visit every 2 weeks.
Visit interval tolerance (days)	±2	±2	±2	±2	±2	±2	±2	±2	—		±3	±3	
Office (O)/Telehealth (T)	T	O	T	O	T	O	T	O	O	O	T	O	
N-terminal pro-B-type natriuretic peptide		X		X		X		X	X	X		X	
(NT-proBNP)		X		X		X		X	X	X		X	
Brain natriuretic peptide (BNP)		X		X		X		X	X	X		X	
Cystatin-C		X		X		X		X	X	X		X	
Troponin, high sensitivity		X		X		X		X	X	X		X	
(hs-cTnT)		X		X		X		X	X	X		X	
C-reactive protein, high sensitivity (hsCRP)								X	X	X			
Urinary albumin to creatinine ratio (UACR)		X		X		X		X	X	X		X	
Dehydroepiandrosterone (DHEA)								X	X			X	
Androstenedione								X	X			X	
Estimated glomerular filtration rate (eGFR)		X		X		X		X	X	X		X	Calculated using Chronic Kidney Disease Epidemiology Collaboration (CKD-EPI) method.
Pharmacokinetic (PK) samples		X		X		X		X	X	X		X	See Pharmacokinetic Sampling Schedule below the SOA for instructions.
Immunogenicity/ADA samples								X	X			X	In the event of systemic drug hypersensitivity reactions (immediate or nonimmediate),

Final

Approved on 09_Apr 2024 GMT
Downloaded by Flora Sam on 09 Apr 2024

Visit number	17	18	19	20	21	22	23	24	ED	UV	801	802	
Weeks from randomization	14	16	18	20	22	24	25	26	—		28	30	**Important Note**: From Visit 17 to Visit 24, site visits are no longer every week, but every 2 weeks. Sites should remind participants to continue their injection on a weekly basis but attend the onsite visit every 2 weeks.
Visit interval tolerance (days)	±2	±2	±2	±2	±2	±2	±2	±2	—		±3	±3	
Office (O)/Telehealth (T)	T	O	T	O	T	O	T	O	O	O	T	O	
													additional unscheduled samples should be collected as detailed in Section 8.3.4. Immunogenicity samples and PK samples for immunogenicity must be taken prior to drug administration.
Stored Samples													
Exploratory biomarker sample		X		X		X		X	X	X		X	Collect stored biomarkers at UV only if participant experienced a worsening HF event.
Exploratory biomarker sample (Urine)								X	X			X	
Participant Diary													
Diary review		X		X		X		X	X				
Diary return								X	X				
Randomization and Dosing													
Process visit using IWRS	X	X	X	X	X	X	X	X	X		X	X	
Dispense study intervention via IWRS		X		X		X							
Participant returns unused study intervention		X		X		X		X	X				
Administer study intervention weekly	X	X	X	X	X	X	X						Between V17 and V24: participants may self-administer injection on a weekly basis or

Final

Approved on 09 Apr 2024 GMT
Downloaded by Flora Sam on 09 Apr 2024

Visit number	17	18	19	20	21	22	23	24	ED	UV	801	802	
Weeks from randomization	14	16	18	20	22	24	25	26	—		28	30	**Important Note**: From Visit 17 to Visit 24, site visits are no longer every week, but every 2 weeks. Sites should remind participants to continue their injection on a weekly basis but attend the onsite visit every 2 weeks.
Visit interval tolerance (days)	±2	±2	±2	±2	±2	±2	±2	±2	—		±3	±3	
Office (O)/Telehealth (T)	T	O	T	O	T	O	T	O	O	O	T	O	
													may come back to site for their weekly injection. If participant come on site for their weekly injection, site needs to remember that the participant must attend visits and respective procedures as defined in the SoA.

Abbreviations: ADA = anti-drug antibody; AE = adverse events; BP = blood pressure; CVD = cardiovascular disease; ECG = electrocardiogram; ED = early discontinuation; eGFR = estimated glomerular filtration rate; HF = heart failure; ICF = informed consent form; IWRS = interactive web-response system; NRS = Numeric Rating Scale; NYHA = New York Heart Association; MI = myocardial infarction; O = office visit; PI = principal investigator; PK = pharmacokinetics; PR = pulse rate; SoA = schedule of activities; SP = study period; T = telehealth visit; V = visit.

Final

Approved on 09 Apr 2024 GMT
Downloaded by Flora Sam on 09 Apr 2024

Pharmacokinetic Sampling Schedule:

Visit Number	Week Relative to Randomization	Collection Time Point Relative to Weekly Dose
2	0	Predose[a, b] and 2 to 4 hr post V2 dose
3	1	Predose
5	3	Predose[a, b]
6	3+	24 to 96 hr post V5 dose[a]
10	7	Predose
11	7+	24 to 96 hr post V10 dose
14	10	Anytime during visit
16	12	Predose[a, b]
18	16	Anytime during visit[a]
20	20	Anytime during visit
22	24	Anytime during visit
24	26	Anytime during visit[a, b]
ED	NA	Anytime during visit[a, b]
UV	NA	Anytime during visit
802	30	Anytime during visit[b]

Abbreviations: NA = not applicable; ED = early discontinuation; UV = unscheduled visit.

[a] On visits where triplicate ECGs are also measured, PK samples should be taken immediately (within 30 min) after ECG measurement.

[b] Immunogenicity (ADA) samples collected with PK at these visits.

Final

Approved on 09 Apr 2024 GMT
Downloaded by Flora Sam on 09 Apr 2024

2. Introduction

LY3540378 is a recombinant fusion protein, which includes an albumin-binding variable domain of a heavy chain only antibody fused via a flexible linker to the N-terminus of a single-chain relaxin. Short-acting recombinant forms of relaxin have been shown to acutely improve dyspnea symptoms during HF events in clinical trials. Long-acting, LY3540378 is being developed for the treatment of patients with worsening chronic HFpEF.

2.1. Study Rationale

HFpEF is a heterogenous clinical syndrome complicated by a high prevalence of co-existing comorbidities with no known single pathophysiological process (van Heerebeek and Paulus 2016). Until the publication of the EMPEROR-Preserved trial (empagliflozin in HFpEF) in October 2021 (Anker et al. 2021), no clinical trial had shown benefit of an experimental therapy in HFpEF. Empagliflozin has been approved for treatment for a wide range of patients with HF and sacubitril/valsartan was granted an expanded label for HF in adult patients with chronic HF, particularly those with LVEF below normal.

Study J3E-MC-EZDB (EZDB) will investigate the effects of 26-week treatment with LY3540378 on LARS in participants with worsening chronic HFpEF. This is the first Phase 2 study, and data from this study will inform dose decisions for the clinical development of LY3540378 for HFpEF.

Final

2.2. Background

In the US, 6.2 million adults have HF (Benjamin et al. 2019), and the number of HF patients is expected to increase 46% by 2030 (Savarese and Lund 2017). Among all patients with HF, at least 50% present clinically with HFpEF (Owan et al. 2006) but its prevalence is increasing. It is estimated that by 2030, the prevalence of HF will increase an additional 46% and US HF costs are expected to be at least $70 billion per year with total cost of caring for HF patients to reach $160 billion (Heidenreich et al. 2013, 2022b). HFpEF patients, if anything, are expected to outnumber HF with reduced ejection fraction.

The prevalence of HFpEF increases with age and is estimated to increase even more in those >70 years (Zile and Brutsaert 2002). Additionally, it is also more common in women. However, in the last 2 decades, the age incidence of HFpEF has declined but this may be regional. For example, in the ASIAN-HF registry, Asian patients with HFpEF were younger and more often men compared with predominantly Western patients (Tromp et al. 2018). Recent data in the USA also showed that African Americans (~65.6 years) presented with incident HFpEF at a younger average age than White patients (~76.7 years) (Lekavich et al. 2021). HFpEF is highly associated with comorbidities, more so than HF with reduced ejection fraction, including hypertension, obesity, diabetes mellitus, atrial fibrillation, CKD, and obstructive sleep apnea, and these comorbidities likely play a pathophysiological role in HFpEF (Solomon et al. 2007; Tromp et al. 2018, Gentile et al. 2021).

Approved on 09 Apr 2024 GMT
Downloaded by Flora Sam on 09 Apr 2024

Management of HFpEF has largely been limited to symptom management and treatment of comorbidities, including blood pressure control, reduction in congestion, preservation of renal function, and control of arrhythmias (Yancy et al. 2013). However, in the most recent 2022-updated ACC/AHA guidelines, SGLT-2i were designated a Class 2A indication as SGLT-2i are beneficial in decreasing HF hospitalizations and cardiovascular mortality in HFpEF patients (Heidenreich et al. 2022a). Conversely, sacubitril/valsartan, an angiotensin receptor-neprilysin inhibitor, was recently designated a Class 2B indication, based on the limited level of evidence, and may be considered for selected patients with LVEF on the lower end of this spectrum (Heidenreich et al. 2022a).

The potential advantages of relaxin in HFpEF could be related to the short-term hemodynamic effects, such as mild blood pressure reduction and long-term benefits such as anti-inflammatory and anti-oxidative actions. The pleiotropic effects are related to the signaling cascade and its downstream mediators (Dschietzig et al. 2009).

Human relaxin-2 (relaxin) is an endogenous hormone that plays a role in the hemodynamic and renovascular adaptive changes during pregnancy (Conrad 2011). Through the RXFP1 receptor, relaxin activates adenylate cyclase, protein kinase A, protein kinase C, phosphatidylinositol 3-kinase, and extracellular signaling-regulated kinase and also interacts with nitric oxide signaling (Bathgate et al. 2013). Serelaxin, a recombinant human relaxin, was shown to acutely improve dyspnea symptoms and reduce worsening HF events through Day 5 in acute HF clinical trials after short-term IV infusion for 48 hours (Dschietzig et al. 2009; Conrad 2011). Since short-term continuous infusion of short acting serelaxin demonstrated several beneficial hemodynamic changes in HF patients (Dschietzig et al. 2009; Ponikowski et al. 2014; Voors et al. 2014), chronic treatment with a long-acting relaxin may also potentially improve outcomes and reduce rehospitalizations and mortality in HF.

Final

LY3540378 is a recombinant fusion protein composed of an albumin-binding variable domain of a heavy chain only antibody fused via a flexible linker to the N-terminus of a single-chain relaxin. LY3540378 maintains similar selectivity between the receptors RXFP1 and RXFP2 as human relaxin-2. LY3540378 is anticipated to exhibit a human terminal half-life of approximately 9 days that supports once-weekly administration. In 3 preclinical models (in Sprague-Dawley rats and cynomolgus monkeys), LY3540378 has similar PD effects as human relaxin-2.

A detailed description of the chemistry, pharmacology, efficacy, and safety of LY3540378 is provided in the IB.

Clinical Study EZDA preliminary results

Study J3E-MC-EZDA (EZDA) is a 4-part, Phase 1, multicenter, participant and investigator blind, SAD, and MAD study in healthy participants to evaluate the safety, tolerability, and pharmacokinetics of single and once-weekly doses of LY3540378. Overall, LY3540378 was well tolerated. Related to treatment, only mild AEs were observed, with the most commonly reported AEs being administration site reactions, such as erythema, bruise, and injection-site reactions.

Approved on 09 Apr 2024 GMT
Downloaded by Flora Sam on 09 Apr 2024

2.3. Benefit/Risk Assessment

More detailed information about the known and expected benefits and risks and reasonably expected AEs of LY3540378 may be found in the IB.

2.3.1. Risk Assessment

Study intervention

Study EZDA clinical data

Preliminary results from Study EZDA in healthy participants indicate that LY3540378 is generally well tolerated (see Section 2.2)

Nonclinical data

Based on the nonclinical data, LY3540378 is not considered to be a high-risk compound.

Potential risks

Based on the current clinical data, nonclinical safety pharmacology and toxicology studies, potential risks for clinical study participants receiving LY3540378 are

- hypotension-related events, and
- systemic allergic or hypersensitivity reactions, or ISRs

Based only on preclinical studies in rats, potential risks also include

- reversible cervix hyperplasia, and
- breast tumor.

Mitigations for potential risks

Hypersensitivity reactions will be managed per Section 8.3.4.

Injection-site reaction assessments will occur throughout the study.

As embryo or fetal studies have not been conducted prior to this study, women of childbearing potential will be excluded.

Orthostatic hypotension will be monitored closely throughout the study, and blood pressure-lowering drugs and diuretics dosing may be lowered as needed at the discretion of the study investigator.

Participants with significant previous abnormal pap smear or mammography will be excluded from the study.

Participants with history of cancer (within the last 3 years) or first-degree family history of breast cancer will be excluded from the study.

Study procedures

Echocardiogram (Echo) will be performed at 3 time points (see the SoA, Section 1.3). There are no specific risks as it is a non-invasive procedure to assess the overall function of the heart.

Final

Approved on 09 Apr 2024 GMT
Downloaded by Flora Sam on 09 Apr 2024

Benefit assessment

The efficacy of LY3540378 for treating worsening chronic HFpEF has not been established. Participants may benefit by receiving personal health information from the physical examinations, frequent engagement with healthcare providers, and from other routine safety assessments performed in this study.

2.3.2. Overall Benefit Risk Conclusion

Considering the measures taken to minimize risk for the participants in this study, the potential risks identified in association with LY3540378 are justified by the anticipated benefits that may be afforded to participants with worsening chronic HFpEF.

Final

Approved on 09 Apr 2024 GMT
Downloaded by Flora Sam on 09 Apr 2024

3. Objectives, Endpoints, and Estimands

Objectives	Endpoints
Primary	
To demonstrate that LY3540378 administered SC QW is superior to placebo for improving atrial myopathy in participants with worsening chronic HFpEF	Change from baseline to Week 26 in LARS
Secondary	
To compare the effect of LY3540378 administered SC QW on participants with worsening chronic HFpEF	Change from baseline to Weeks 12 and 26 in<br>• Log-transformed NT-proBNP<br>• LAEDVI<br>• LAESVI<br>• eGFR (CKD-EPI Creatinine-Cystatin equation [2021])<br>• serum creatinine, and<br>• cystatin-C
To assess safety and tolerability of LY3540378 administered SC QW	• AE overall<br>• safety topics of special interest
Tertiary	
To compare the effect of LY3540378 administered SC QW on participants with worsening chronic HFpEF	• Change from baseline to Weeks 12 and 26 in<br>○ LA emptying fraction<br>○ LVGLS<br>○ E/A<br>○ E/e'<br>○ LVM (LVMI)<br>○ high sensitivity troponin, (hs-cTnT)<br>○ NYHA class, and<br>○ BNP<br>• Change from baseline to the average of Week 24 and Week 26 in log-transformed NT-proBNP<br>• Blood pressure and pulse rate

Final

Approved on 09_Apr 2024 GMT
Downloaded by Flora Sam on 09 Apr 2024

Clinical outcome events of HF	Incidence of<br>• All deaths (CV and non-CV)<br>• HF event:<br>○ hospitalized (HF hospitalization) and<br>○ non-hospitalized HF events (urgent outpatient visits, unscheduled office, or emergency visit for HF)
Change in outpatient hemodynamic CV medications	Change in the dosing of<br>• diuretics (loop and thiazide)<br>• RAAS inhibitor<br>• SGLT-2i<br>• Beta blockers<br>• ARNI, and<br>• MRA
To assess the effect of LY3540378 on patient-reported outcomes	Change from baseline through Week 26 of<br>• Severity of Most Bothersome Symptom (either dyspnea, edema, or fatigue)<br>• Dyspnea NRS<br>• Edema NRS<br>• Fatigue NRS<br>• PGIS-HF Overall Health<br>• PGIC-HF Overall Health<br>• PGIS-HF Symptom Severity<br>• PGIC-HF Symptom Severity, and<br>• KCCQ<br>○ Total Symptom Score<br>○ Clinical Summary Score, and<br>○ Overall Summary Score
To assess presence of anti-LY3540378 antibodies	ADAs against LY3540378 including<br>• treatment-emergent ADAs, and<br>• neutralizing antibodies
To assess LY3540378 PK and the relationship between LY3540378 dose or exposure and clinical endpoints and potential participant factors that may influence these relationships	PK parameters of LY3540378 (C_{max}, AUC).<br>Dose or exposure-response analyses for key efficacy and safety endpoints

Final

Approved on 09 Apr 2024 GMT
Downloaded by Flora Sam on 09 Apr 2024

Abbreviations: ADA = anti-drug antibody; AE = adverse event; ARNI = angiotensin receptor neprilysin inhibitor; BNP = brain natriuretic peptide; CKD-EPI = Chronic Kidney Disease Epidemiology Collaboration; CV = cardiovascular; ECG = electrocardiogram; eGFR = estimated glomerular filtration rate; HF = heart failure; HFpEF = heart failure with preserved ejection fraction; KCCQ = Kansas City Cardiomyopathy Questionnaire; LA = left atrium; LAEDVI = left atrial end-diastolic volume index; LAESVI = left atrial end-systolic volume index; LARS = left atrial reservoir strain; LVGLS = left ventricular global longitudinal strain; LVM = left ventricular mass; LVMI = left ventricular mass index; MRA = mineralocorticoid receptor antagonists; NRS = Numeric Rating Scale; NT-proBNP = N-terminal pro-B-type natriuretic peptide; NYHA = New York Heart Association; PGIC-HF = Patient Global Impression of Change – Heart Failure; PGIS-HF = Patient Global Impression of Status – Heart Failure; PRO = patient-reported outcome; RAAS = Renin-angiotensin-aldosterone system; SC = subcutaneous; SGLT-2i = sodium-glucose cotransporter-2 inhibitor; QW = weekly.

Primary estimand/coprimary estimand

The primary clinical question of interest is

> What is the treatment difference in LARS change from baseline after 26 weeks of treatment in study participants who would have completed the treatment period?

Efficacy estimand attributes

This table describes the efficacy estimand attributes.

Efficacy Estimand Attribute	Description
Population	Participants who meet the inclusion criteria.<br>Further details can be found in Sections 5 and 9.
Endpoint	Change from baseline in LARS at Week 26.
Treatment condition	The randomized treatment with allowance for dose modification based on hypotension (Section 6.5) and TD for safety (Section 7.1.4).
Population-level summary	Difference in mean absolute changes in LARS at Week 26 between LY3540378 and placebo.

Abbreviations: LARS = left atrial reservoir strain; TD = temporary discontinuation.

Intercurrent events

The intercurrent event, "permanent discontinuation of intervention," is handled by the hypothetical strategy. The potential outcome of interest is the response in the efficacy measurement if participants adhere to the randomized treatment.

Rationale for the efficacy estimand

This Phase 2 study aims to study the efficacy of LY3540378 under the ideal condition that all participants adhere to the randomized treatment.

The same estimand for the primary objective will be used for the secondary clinical response endpoints.

Unless specified otherwise, safety and tolerability assessments will be guided by an estimand comparing safety of LY3540378 doses with placebo irrespective of adherence to study intervention, including data collected during the treatment period and safety follow-up from all randomized participants who are exposed to at least 1 dose of study drug, regardless of adherence of study drug.

Final

Approved on 09 Apr 2024 GMT
Downloaded by Flora Sam on 09 Apr 2024

4. Study Design

4.1. Overall Design

Study EZDB is a Phase 2, multicenter, randomized, double-blind, placebo-controlled study that will investigate the effects of treatment with LY3540378 compared with placebo on participants with worsening chronic HFpEF.

Participants will be randomized 1:2:2:2 to the following intervention groups:

- LY3540378 25 mg SC QW
- LY3540378 50 mg SC QW
- LY3540378 100 mg SC QW, and
- placebo.

Intervention administration is by subcutaneous injection, and dosing will occur every week.

The maximum total duration of study participation for each participant, including screening and safety follow-up periods, is approximately 32 weeks, across the following study periods:

- Screening: up to 14 days
- Double-Blind Treatment: 26 weeks, and
- Safety Follow-Up: 4 weeks.

Final

Screening

Interested participants will sign the appropriate informed consent document(s) prior to completion of any procedures. The investigator will review symptoms, risk factors, medical history, concomitant medications, and other inclusion and exclusion criteria prior to any diagnostic procedures. If the participant is eligible after this review, then the site will perform the diagnostic procedures to confirm eligibility.

Double-blind treatment and assessment period

This is the general flow during the treatment and assessment period:

- complete baseline procedures and sample collection
- participants are randomly assigned to an intervention group, and
- participants complete all visit procedures including efficacy assessments, safety monitoring, study intervention dosing, and post-dosing sample collection.

Safety follow-up visits 801 and 802

Study personnel and participants complete all visit procedures described in the SoA. The investigator will follow up on the participant's transition from study intervention.

An independent adjudication committee will adjudicate all-cause mortality and HF events. See Section 10.1.5 for details related to adjudicated events and committee structure.

Approved on 09 Apr 2024 GMT
Downloaded by Flora Sam on 09 Apr 2024

4.2. Scientific Rationale for Study Design

Primary endpoint rationale

HFpEF patients have an atrial myopathy, and this can be measured non-invasively by LARS. LARS is associated with adverse clinical outcomes and poor prognosis in HFpEF. An increased PCWP with exercise and lower peak oxygen consumption on cardiopulmonary exercise testing is seen in worsening chronic HFpEF and associated with a *reduced* LARS (Patel et al. 2021). Thus, a *stabilization* and/or an *increase* in LARS with a therapy targeting LA myopathy is expected to improve outcomes in HFpEF. Additionally, LARS is also strongly related to LV strain, which is abnormal in HFpEF and LV strain is also associated with adverse outcome (Shah et al. 2015). Thus, LARS provides an integrated measures of LV and LA function and hemodynamics.

Overall design description

The 26 weeks' duration of the treatment period is a reasonable time frame to observe efficacy for the treatment of HFpEF, which will inform the dose level in future clinical studies.

The follow-up visits after the last dose are designed to capture any additional safety signals.

Placebo is chosen as the control treatment to assess whether any observed effects are treatment related or simply reflect the study conditions. The double-blind design minimizes bias on safety assessments and allows a more robust comparison among LY3540378 doses and placebo.

In this study, collection of demographic information includes race and ethnicity. The scientific rationale is based on the need to assess variable response in safety and/or efficacy based on race or ethnicity. This question can be answered only if all the relevant data are collected.

Final

4.3. Justification for Dose

LY3540378 Phase 2 doses of 25, 50, and 100 mg administered SC QW were selected in consideration of the following factors:

- Safety and tolerability data from Phase 1 development in healthy participants.
- Margin of safety for the highest dose of SC 100 mg QW in this study has anticipated exposure multiples between 3 and 25 to the no-observed-adverse-effect level in rats and monkeys in the 6-month toxicology studies.
- Following QW dosing, LY3540378 SC 25 mg QW and 120 mg QW showed robust increase in effective renal plasma flow of at least 30% at Week 5 in healthy participants.
- PK exposure-response analysis of all available SC effective renal plasma flow data following single and multiple doses of LY3540378 combined.
- This dose range was chosen to improve the chances of achieving efficacy in HFpEF patients since limited information is available to guide translation of effective renal plasma flow pharmacology from healthy participants to clinical improvements in HFpEF patients.
- The safety-efficacy data produced from the selected dose range will inform dose selection for future development of LY3540378 in the HFpEF patient population.

Approved on 09_Apr 2024 GMT
Downloaded by Flora Sam on 09 Apr 2024

4.4. End of Study Definition

The end of the study is defined as the date of the last visit of the last participant in the study globally.

A participant is considered to have completed the study if the participant has completed all periods of the study including the last visit shown in the SoA.

Final

Approved on 09 Apr 2024 GMT
Downloaded by Flora Sam on 09 Apr 2024

5. Study Population

Prospective approval of protocol deviations to recruitment and enrollment criteria, also known as protocol waivers or exemptions, is not permitted.

5.1. Inclusion Criteria

Participants are eligible to be included in the study only if all the following criteria apply:

Age

1. Have a known clinical diagnosis of HFpEF or "diastolic heart failure" and must be at least 18 years of age or the legal age of consent in the jurisdiction in which the study is taking place at the time of signing the informed consent.

Type of participant and disease characteristics

2. Deleted
3. Have experienced an index event, defined as

   A. a recent hospitalization for HF requiring ≥1 bolus dose of IV diuretics,

   OR

   B. an out-of-hospital encounter (for example, Emergency Room, clinic visit, infusion clinic) for HF requiring ≥1 bolus dose of IV diuretics at that index event. A single continuous infusion is also allowed.
4. Documented LVEF of ≥50% within 12 months prior to V1 (screening); as measured by echocardiography, radionuclide ventriculography, invasive angiography, MRI, or CT. Evidence of documentation of LVEF ≥50% may also include patient medical records, discharge notes or a referral letter from the patient's physician or referring physician that details the patient's medical history.
5. Have NYHA Class II-IV symptomatology as assessed at V1 (screening).
6. Had evidence of clinical HF syndrome consisting of

   A. Hospitalization for WHF with intravascular volume overload (the index event), as determined by the investigator, based on appropriate supportive documentation at randomization, and defined by ≥2 of the following:

   - dyspnea
   - jugular venous distention
   - pitting edema in lower extremities (>1+)
   - ascites
   - pulmonary congestion on chest X-ray
   - pulmonary rales AND patient received treatment with IV diuretics.

   OR

   B. Treatment for an urgent visit outside of being hospitalized with WHF and intravascular volume overload (the index visit) requiring treatment with IV diuretics

Final

Approved on 09 Apr 2024 GMT
Downloaded by Flora Sam on 09 Apr 2024

(defined as ≥1 IV bolus dose) such as in the outpatient setting/emergency room/observation unit/infusion clinic with a clinical response within the past 2 weeks prior to randomization. Urgent visit is defined as an unplanned visit for HF defined by ≥2 of the following:

- dyspnea
- jugular venous distention
- pitting edema in lower extremities (>1+)
- ascites
- pulmonary rales on lung examination.

7. NT-proBNP (>300 [sinus rhythm] or 600 pg/mL [atrial fibrillation or atrial flutter] OR BNP (>100 [sinus rhythm] or 200 pg/mL [atrial fibrillation or atrial flutter]) at screening (Visit 1, determined by local laboratory)

   Note: The presence or absence of atrial fibrillation or atrial flutter to determine the appropriate cut-off for a given BNP or NT-proBNP sample should be evaluated using the ECG performed at V1 (screening) prior to the collection of the BNP or NT-proBNP sample.

8. eGFR of >20 mL/min/1.73 m^2 at V1 (screening; determined by local laboratory), derived from serum creatinine values, age, and sex based on the CKD-EPI equation (Inker et al. 2021).

9. If the participant is screened (V1):

A. after the index event in the outpatient setting, the diuretic doses must have transitioned to oral loop diuretic **before** randomization (V2). In other words, chronic oral diuretic should have been prescribed and/or administered, OR

B. during the index event (such as while hospitalized or at an urgent clinic visit or emergency room), the diuretic can be administered either as an IV or oral formulation prior to randomization (V2).

Final

Sex and contraceptive/barrier requirements

10. Males and females will be eligible for this study.

    a. Women not of childbearing potential may participate in this trial. (WOCBP are excluded from the trial.)
       See Appendix 10.4 for definitions.
    b. Males who agree to use highly effective or effective methods of contraception may participate in this trial.
       See Appendix 10.4 for definitions.

Contraceptive use by participants should be consistent with local regulations regarding the methods of contraception for those participating in clinical studies. For the contraception requirements of this protocol, see Appendix 10.4.

Informed consent

Approved on 09 Apr 2024 GMT
Downloaded by Flora Sam on 09 Apr 2024

11. Are capable of giving signed informed consent as described in Section 10.1.3, which includes compliance with the requirements and restrictions listed in the ICF and in this protocol.

Other inclusions

12. Are reliable and willing to make themselves available for the duration of the study and are willing to follow study procedures, such as
    - self-inject intervention, and
    - store and take provided study interventions as directed.

    Note: Persons with physical limitations or unable to perform the injections must have the assistance of an individual trained to inject the intervention or come onsite for weekly injection.

5.2. Exclusion Criteria

Participants are excluded from the study if any of the following criteria apply:

Cardiovascular conditions

13. Prior documentation of LVEF ≤45% in the past 12 months.
14. Have had acute coronary syndrome or percutaneous coronary intervention, coronary artery bypass graft, cardiac mechanical support implantation, and PVA (pulmonary vein isolation ablation) within 3 months prior to V2 (randomization), or any other cardiac surgery planned during the study.
15. Have had LVAD or cardiac transplantation or have cardiac transplantation planned during the study.
16. Have hypertrophic cardiomyopathy (obstructive or nonobstructive), restrictive cardiomyopathy, active myocarditis, constrictive pericarditis, cardiac sarcoidosis, known amyloid cardiomyopathy, or inherited cardiomyopathy.
17. Deleted
18. Have a history of an uncorrected cyanotic cardiac disease affecting LV function.
19. Have a severe uncorrected valvular disease.
20. The index admission or index visit for worsening chronic HF was not triggered primarily by intravascular volume overload but triggered by
    - pulmonary embolism
    - cerebrovascular accident
    - acute myocardial infarction (Type 1 MI)
    - significant arrhythmia, for example, sustained ventricular tachycardia, or bradycardia with sustained ventricular arrhythmia <45 bpm (however, any atrial fibrillation or flutter is permitted)
    - serious or systemic infection
    - severe anemia, or
    - exacerbation of COPD.
21. In the opinion of the PI, have any medical complication arising during HF index admission that prolongs the hospitalization.

Final

Approved on 09 Apr 2024 GMT
Downloaded by Flora Sam on 09 Apr 2024

22. Time from hospitalization discharge to randomization is >2 weeks for participants who are not randomized during hospitalization OR duration of the HF hospitalization lasting more than 4 weeks.
23. Are hospitalized for worsening HF event or received treatment for an urgent HF visit outside of being hospitalized with WHF, **after** V1 (screening) and **before** V2 (randomization) (see Section 5.4 for rescreening).

Other medical conditions

24. Have a chronic pulmonary/lung condition (COPD, pulmonary arterial hypertension, etc.) as defined by chronic oxygen dependence. Night-time oxygen is not exclusionary.
25. Uncorrected thyroid disease.
26. During the index event, hemoglobin <10 g/dL (or local lab at screening). However, the participant is considered eligible if subsequently the hemoglobin is ≥10 g/dL before V2 (randomization).
27. Patients requiring dialysis at screening.
28. Have, within 3 years prior to screening, a history of an active or untreated malignancy or are in remission from a clinically significant malignancy.
    Exceptions:
    - basal or squamous cell skin cancer.
29. Male and female participants with a personal or family history (first-degree relatives) of breast cancer.
30. Women with a history of a mammography with clinically significant abnormal findings.
31. Women with a history of clinically significant abnormal pap smear.
32. Patients have any history of bleeding or coagulation disorder causing a bleeding diathesis, other bleeding diathesis, or significant, nontraumatic bleeding episodes, such as from a gastrointestinal source.
33. Symptomatic hypotension or a systolic BP <100 mmHg at the Screening Visit and on the day of V2 (randomization).
34. SBP ≥180 mmHg at V2 (randomization).
35. Have a history of or current significant psychiatric disorders considered clinically significant in the opinion of the investigator.
36. Chronic alcohol or drug abuse or any condition that, in the investigator's opinion, makes them an unreliable trial participant or unlikely to complete the trial.
37. Any other clinical condition that would jeopardize the participant's safety while participating in this trial or may prevent the participant from adhering to the trial protocol.

Diagnostic assessments

38. During the index event, evidence of hepatic insufficiency classified as:
    - a documented Child-PUGH B or C,
    - a documented ALT or AST >5x ULN, or
    - evidence of active/chronic liver disease at the PI discretion.

Final

Approved on 09 Apr 2024 GMT
Downloaded by Flora Sam on 09 Apr 2024

39. Have a suboptimal ECHO image quality assessed by a central imaging laboratory at screening.

Prior/concomitant therapy

40. Have received IV inotropic therapy within 30 days before the Screening Visit.

Exception: renally dosed (≤3 µg/kg/min) IV dopamine is permitted. Although not inotropes, IV carperitide and IV tolvaptan are permitted.

41. Are currently treated with hormone replacement therapy at the time of screening, including but not limited to testosterone, estrogen, and DHEA.

Prior or concurrent clinical study experience

42. Are currently enrolled in any other clinical study involving an intervention or any other type of medical research, judged not to be scientifically or medically compatible with this study by the investigator.
43. Have participated, within the last 30 days, in a clinical trial involving an intervention. If the previous intervention has a long half-life, 3 months or 5 half-lives, whichever is longer, should have passed.

Other exclusions

44. Are unwilling to have a cardiac echocardiogram.
45. Are Lilly employees or are employees of any third party involved in the study who require exclusion of their employees.
46. Are investigator site personnel directly affiliated with this study and/or their immediate families. Immediate family is defined as a spouse, parent, child, or sibling, whether biological or legally adopted.
47. Are unsuitable for inclusion in the study in the opinion of the investigator.
48. Index event is the first (de novo) occurrence or diagnosis of HFpEF.

Final

5.3. Lifestyle Considerations

Participants must not donate blood or sperm for the duration of the study and for 135 days following the study.

5.4. Screen Failures

A screen failure occurs when a participant who consents to participate in the clinical study is not subsequently enrolled in the study. A minimal set of screen failure information is required to ensure transparent reporting of screen failure participants to meet the Consolidated Standards of Reporting Trials publishing requirements and to respond to queries from regulatory authorities. Minimal information includes demography, screen failure details, eligibility criteria, and any SAE.

Individuals who do not meet the criteria for participation in this study (screen failure) may be rescreened at the investigator’s discretion after **another, subsequent** worsening HF index event, provided that there are at least 30 days between **subsequent** worsening HF index events.

Approved on 09_Apr 2024 GMT
Downloaded by Flora Sam on 09 Apr 2024

If, in the opinion of the investigator, an ineligible laboratory test result is the result of an error or extenuating circumstance, then that parameter can be retested once (within 24 hours of the initial, local laboratory test) without the participant having to be rescreened.

Note that participants who are rescreened will need to have a repeat echocardiogram collected after the subsequent worsening HF index event.

Rescreened participants should be assigned a new participant number for every screening or rescreening event.

5.5. Criteria for Temporarily Delaying Enrollment of a Participant

This section is not applicable.

Final

Approved on 09 Apr 2024 GMT
Downloaded by Flora Sam on 09 Apr 2024

6. Study Interventions and Concomitant Therapy

Study intervention is defined as any medicinal product(s) or medical device(s) intended to be administered to or used by a study participant according to the study protocol.

6.1. Study Interventions Administered

Intervention Name	LY3540378			Placebo
Dosage Level(s)	25 mg	50 mg	100 mg	N/A
Route of Administration	Subcutaneous injection			
Authorized as defined by EU CTR No 536/2014[a]	Not authorized as defined by EU CTR			

Abbreviations: EU CTR = European Union clinical trial regulation.

a “Authorized investigational medicinal product” means a medicinal product authorized in accordance with Regulation (EC) No 726/2004 or in any Member State concerned in accordance with Directive 2001/83/EC, irrespective of changes to the labeling of the medicinal product, which is used as an investigational medicinal product; “Authorized auxiliary medicinal product” means a medicinal product authorized in accordance with Regulation (EC) No 726/2004, or in any Member State concerned in accordance with Directive 2001/83/EC, irrespective of changes to the labeling of the medicinal product, which is used as an auxiliary medicinal product.

Final

Packaging and labeling

Study interventions will be supplied by the sponsor or its designee in accordance with current Good Manufacturing Practice. Study interventions will be labeled as appropriate for country requirements.

LY3540378 frequency of administration and guidance for missed doses

LY3540378 should be administered once weekly at approximately the same time and day each week. All injections will need to be completed within 10 minutes. The actual date, time, and injection-site location of all dose administrations will be recorded in the diary by the participant. If a dose is missed, it should be administered as soon as possible if at least 3 days (72 hours) remain until the next scheduled dose. If less than 3 days remain before the next scheduled dose, skip the missed dose and administer the next dose on the regularly scheduled day. In each case, participants can then resume their regular once-weekly dosing schedule. The day of weekly administration can be changed, if necessary, only if the last dose has been administered at least 3 days earlier.

Anatomical location of injections

Subcutaneous injections of LY3540378 and placebo will be administered rotating between left and right abdominal regions, and upper and lower quadrants. Injections may be administered in the thigh, arm, or buttock, at the discretion of PI. Refer to the Instructions for Use for complete instructions on dose administration.

Approved on 09 Apr 2024 GMT
Downloaded by Flora Sam on 09 Apr 2024

6.2. Preparation, Handling, Storage, and Accountability

The investigator or designee must confirm appropriate storage conditions have been maintained during transit for all study intervention received and any discrepancies are reported and resolved before use of the study intervention.

Only participants enrolled in the study may receive study intervention. Only authorized study personnel may supply, prepare, or administer study intervention. The participant or caregiver may also administer the study intervention. All study interventions must be stored in a secure, environmentally controlled, and monitored (manual or automated) area in accordance with the labeled storage conditions with access limited to the investigator and authorized study personnel.

The investigator or authorized study personnel are responsible for study intervention accountability, reconciliation, and record maintenance, that is, receipt, reconciliation, and final disposition records.

Further guidance and information for the final disposition of unused study interventions are provided in the Pharmacy Manual.

Participant responsibilities

In-use storage conditions are expected to be followed according to the Instructions for Use provided by the sponsor. Study participants will be trained on the proper storage and handling of the study intervention.

Final

6.3. Measures to Minimize Bias: Randomization and Blinding

Randomization

All participants will be centrally assigned to randomized study intervention using an IWRS. Before the study is initiated, the log-in information and directions for the IWRS will be provided to each site.

For participants who joined study before amendment (b), they were randomized 1:1:1:1 to the following intervention groups:

- LY3540378 25 mg SC QW
- LY3540378 50 mg SC QW
- LY3540378 100 mg SC QW, and
- placebo.

For participants who joined study after amendment (b), will be randomized 1:2:2:2 to the above intervention groups.

Placebo participants will be randomly assigned so that each dose cohort will have a matching placebo cohort that receives the same dose volume to maintain the study blind.

Study intervention will be dispensed at the study visits summarized in SoA.

Returned study intervention should not be re-dispensed to the participants.

Approved on 09_Apr 2024 GMT
Downloaded by Flora Sam on 09 Apr 2024

Stratification

For between-group comparability, participant randomization will be stratified by

- evidence of atrial fibrillation or atrial flutter on the screening ECG (Yes, No)
- region (North America, Latin America, Europe and other countries, Asia).

Randomization into 1 stratum may be discontinued at the discretion of the sponsor.

To avoid over-representation of participants with atrial fibrillation in the trial, randomization of participants with atrial fibrillation on their screening ECG may be stopped at the discretion of the sponsor when this randomized cohort comprises approximately 40% of the total planned randomized population. The exact proportion of participants randomized with atrial fibrillation and/or atrial flutter will be determined by the sponsor following a review of the blinded screening characteristics of this group compared to those without atrial fibrillation and/or atrial flutter.

Blinding

This is a double-blind study in which participants, care providers, investigators, outcomes assessors, etc. are blinded to study intervention. The IWRS will be programmed with blind-breaking instructions. In case of an emergency, the investigator has the sole responsibility for determining if unblinding of a participants' intervention assignment is warranted. Participant safety must always be the first consideration in making such a determination. If the investigator decides that unblinding is warranted, the investigator should make every effort to contact the sponsor prior to unblinding a participant's intervention assignment unless this could delay emergency treatment for the participant. If a participant's intervention assignment is unblinded, the sponsor must be notified immediately within 24 hours of this occurrence. The date and reason that the blind was broken must be recorded.

Final

If an investigator, site personnel performing assessments, or participant is unblinded, the participant must be discontinued from the study. In cases where there are ethical reasons to have the participant remain in the study, the investigator must obtain specific approval from a sponsor for the participant to continue in the study.

6.4. Study Intervention Compliance

Study intervention will be administered under medical supervision by the investigator or designee. The dose of study intervention and study participant identification will be confirmed prior to the time of dosing. The date and time of each dose administered will be recorded in the source documents and will be provided to the sponsor as requested.

When participants self-administer study intervention(s) at home, compliance with study intervention will be assessed at visits indicated in the SoA (Section 1.3). Compliance will be assessed by counting returned vials and documented in the source documents.

A record of the number of LY3540378 vials dispensed to and taken by each participant must be maintained and reconciled with study intervention and compliance records. Intervention start and stop dates, including dates for intervention delays and/or dose reductions, will also be recorded in the CRF.

Approved on 09 Apr 2024 GMT
Downloaded by Flora Sam on 09 Apr 2024

6.5. Dose Modification

This section applies to participants receiving 100 mg (or matching placebo) only.

Holding and potential dose modification procedures apply if the participant experiences the following TD criteria:

- symptomatic hypotension or
- SBP <90 mmHg

When any of the above TD criteria are met, hold study intervention, and document in the CRF. The participant continues attending visits and performing all other procedures and assessments as described in the SoA (Section 1.3).

At each visit, the investigator will review TD criteria. Participants who continue to meet TD criteria will continue to have their dose held.

TD criteria met for	Then	If participant no longer meets TD criteria **on next visit**, then restart dose at
First occurrence	Investigator adjusts concomitant medications AND holds study drug.	100 mg
Second occurrence[a]	Investigator holds study drug.	100 mg
Third occurrence[a]	Investigator holds study drug and contacts the sponsor.	50 or 100 mg. And up titrate from 50 to 100 mg or lower back from 100 to 50 mg as needed, per investigator judgment

Abbreviation: TD = temporary discontinuation.

a Occurrence is defined as each visit the participant presents meeting TD criteria, whether as consecutive visits or intermittent visits, and is cumulative through the study.

Note: Participants in the 100-mg group who are receiving placebo continue to receive placebo when dosing is resumed.

6.6. Continued Access to Study Intervention after the End of the Study

LY3540378 will not be made available to participants after conclusion of the study.

6.7. Treatment of Overdose

For this study, any dose of study intervention greater than the dose assigned through randomization will be considered an overdose.

Sponsor does not recommend specific treatment for an overdose.

Approved on 09 Apr 2024 GMT
Downloaded by Flora Sam on 09 Apr 2024

Final

In the event of an overdose, the investigator should

- contact the medical monitor immediately
- evaluate the participant to determine, in consultation with the medical monitor, whether study intervention should be interrupted or whether the dose should be reduced
- consider holding diuretics and/or blood pressure-lowering medications if clinically appropriate
- closely monitor the participant for any AE or SAE and laboratory abnormalities, and
- obtain a plasma sample for PK analysis within 3 days from the date of the last dose of study intervention if requested by the medical monitor (determined on a case-by-case basis).

6.8. Concomitant Therapy

Any medication or vaccine, including over-the-counter or prescription medicines, vitamins, and/or herbal supplements, that the participant is receiving at the time of enrollment or receives during the study must be recorded along with

- reason for use
- dates of administration including start and end dates, and
- dosage information including dose and frequency for concomitant therapy of special interest.

Final

Standard of care for heart failure

Both American College of Cardiology/American Heart Association and European Society of Cardiology guidelines recommend symptom management with diuretic agents in patients with excess volume, as well as aggressive risk factor management for comorbidities for the treatment of HFpEF (van der Meer et al. 2019). Optimization of volume status and proactive adjustment of diuretic doses will help control symptoms and volume overload. SGLT-2i is now a Class 2A indication in participants with HFpEF, as SGLT-2is have been shown to decrease HF hospitalizations and cardiovascular mortality (Heidenreich et al. 2022a).

Participants should remain on stable doses of medications to treat comorbidities such as hypertension. Dose reduction or discontinuation of such background therapies should be avoided unless all other measures fail to improve the participant's condition. However, if the participant's condition warrants a change in any of these medications, it will be allowed at the discretion of the investigator.

The sponsor should be contacted if there are any questions regarding concomitant or prior therapy.

Approved on 09 Apr 2024 GMT
Downloaded by Flora Sam on 09 Apr 2024

7. Discontinuation of Study Intervention and Participant Discontinuation/Withdrawal

Discontinuation of specific sites or of the study as a whole are handled as part of Appendix 1.

7.1. Discontinuation of Study Intervention

When necessary, a participant may be permanently discontinued from study intervention. If so, the participant will remain in the study and follow procedures for remaining study visits.

A participant should be permanently discontinued from study intervention if

- the participant becomes pregnant during the study, or
- in the opinion of the investigator, the participant should permanently discontinue the study intervention for safety reasons.

7.1.1. Hepatic Criteria for Study Intervention Interruption or Discontinuation

See Section 8.3.5 for hepatic criteria for study intervention interruption or discontinuation.

7.1.2. QTc Stopping Criteria

If a clinically significant finding is identified (including, but not limited to changes from baseline in QT interval corrected using Fridericia's formula [QTcF]) after enrollment, the investigator or qualified designee will determine if the participant can continue in the study and if any change in participant management is needed. This review of the ECG printed at the time of collection must be documented. Any new clinically relevant finding should be reported as an AE.

Final

7.1.3. Hypersensitivity Reactions

If the investigator determines that a systemic hypersensitivity reaction has occurred related to study intervention administration, the participant may be permanently discontinued from the study intervention, and the sponsor's designated medical monitor should be notified. If the investigator is uncertain about whether a systemic hypersensitivity reaction has occurred and whether discontinuation of study intervention is warranted, the investigator may consult the sponsor.

7.1.4. Temporary Discontinuation

In certain situations, after randomization, the investigator may need to temporarily discontinue (interrupt) study intervention, for example, due to an AE, ISR, or a clinically significant laboratory value. If study intervention interruption is due to an AE, the event is to be documented and followed according to the procedures in Section 8.3 of this protocol. Every effort should be made by the investigator to maintain patients on study intervention and to restart study intervention after any temporary interruption, as soon as it is safe to do so. The data related to temporary interruption of study intervention will be documented in source documents and entered in the eCRF.

Criteria for temporary discontinuation

Approved on 09 Apr 2024 GMT
Downloaded by Flora Sam on 09 Apr 2024

The investigator may temporarily interrupt study treatment, due to an AE, clinically significant laboratory value, hospital visits, travel, or shortage of study treatment supply.

Investigator should inform the sponsor when study intervention has been temporarily discontinued after 3 consecutive weeks for symptomatic hypotension. This information should be documented by the investigator.

For participants receiving 100 mg, certain TD criteria (symptomatic hypotension or SBP <90 mmHg) could result in dose modification. See Section 6.5.

Guidance when temporary discontinuation of study intervention occurs

Every effort should be made by the investigator to maintain participants in the study and to restart study intervention promptly, as soon as it is safe to do so.

Participants will continue their study visits and follow-up according to the SoA.

Participants should resume the dose prescribed before the temporary dosing interruption at the discretion of the investigator.

Recording temporary discontinuation of study intervention

The dates of study intervention interruption and restart must be documented in source documents and entered on the CRF.

Participant noncompliance should not be recorded as interruption of study intervention on the CRF.

Final

7.2. Participant Discontinuation/Withdrawal from the Study

Discontinuation is expected to be uncommon.

A participant may withdraw from the study:

- at any time at the participant's own request
- at the request of the participant's designee, for example, parents or legal guardian
- at the discretion of the investigator for safety, behavioral, compliance, or administrative reasons
- if enrolled in any other clinical study involving an investigational product, or enrolled in any other type of medical research judged not to be scientifically or medically compatible with this study.

At the time of discontinuing from the study, if possible, the participant will complete procedures for an early discontinuation visit and safety follow-up, as shown in the SoA. If the participant has not already discontinued the study intervention, the participant will be permanently discontinued from the study intervention at the time of the decision to discontinue the study.

If the participant withdraws consent for disclosure of future information, the sponsor may retain and continue to use any data collected before such a withdrawal of consent. If a participant withdraws from the study, the participant may request destruction of any samples taken and not tested, and the investigator must document this in the site study records.

Approved on 09 Apr 2024 GMT
Downloaded by Flora Sam on 09 Apr 2024

7.3. Lost to Follow Up

A participant will be considered lost to follow-up if he or she repeatedly fails to return for scheduled visits and is unable to be contacted by the study site. Site personnel or designee are expected to make diligent attempts to contact participants who fail to return for a scheduled visit or were otherwise unable to be followed up by the site.

Final

Approved on 09 Apr 2024 GMT
Downloaded by Flora Sam on 09 Apr 2024

8. Study Assessments and Procedures

Study procedures and their timing are summarized in the SoA.

Immediate safety concerns should be discussed with the sponsor immediately upon occurrence or awareness to determine if the participant should continue or discontinue study intervention.

Adherence to the study design requirements, including those specified in the SoA, is essential and required for study conduct.

All screening evaluations must be completed and reviewed to confirm that potential participants meet all eligibility criteria. The investigator will maintain a screening log to record details of all participants screened and to confirm eligibility or record reasons for screening failure, as applicable.

8.1. Efficacy Assessments

8.1.1. Primary Efficacy Assessment

The primary efficacy measurement in this study is the change in LARS from baseline to Week 26. LARS is a non-invasive assessment of elevated left filling pressures and correlates with PCWP. LA reservoir strain quantifies the deformation of the left atrium during atrial filling, whereby less deformation (lower strain) has been associated with adverse LA remodeling and increased fibrosis.

The LA plays an integral role in the pathophysiology and prognosis of HFpEF. Beyond LA size, LA mechanical dysfunction has gained a considerable amount of attention recently. LARS measured by two-dimensional (2D) speckle-tracking echocardiography, is a key LA parameter quantifying LA compliance and function. LARS is also a sensitive parameter to assess LV filling pressure (Tan et al. 2021) and correlates with PCWP (Wakami et al. 2009). Morris et al. (2018) shows incremental diagnostic value of LARS over LA volume in detecting LV diastolic dysfunction. Lower LARS is associated with higher risk of HF hospitalization and CV death (Santos et al. 2016), after adjusting for LA volume.

8.1.2. Other Efficacy Assessments

Secondary efficacy assessments for this study are

- Log-transformed NT-proBNP change from baseline to Week 12 and 26 (see Section 8.1.2.1)
- LAEDVI change from baseline to Week 12 and 26 (see Section 8.1.2.2)
- LAESVI change from baseline to Week 12 and 26 (see Section 8.1.2.2)
- eGFR, calculated by CKD-EPI Creatinine-Cystatin equation (2021), change from baseline to Week 12 and 26 (see Section 8.1.2.1)
- serum creatinine change from baseline to Week 12 and 26 (see Section 8.1.2.1), and
- Cystatin-C change from baseline to Week 12 and 26 (see Section 8.1.2.1).

Final

Approved on 09 Apr 2024 GMT
Downloaded by Flora Sam on 09 Apr 2024

8.1.2.1. Pharmacodynamic Markers

At times specified in the SoA, blood samples will be collected to measure changes in levels of these markers to assess worsening chronic HFpEF:

- NT-proBNP
- Creatinine, to calculate eGFR
- Cystatin-C, to calculate eGFR, and
- BNP

8.1.2.2. Echocardiogram

Two-dimensional ECHO with pulsed and tissue Doppler sampling will be performed locally by personnel qualified by experience and training as specified in the SoA (Section 1.3).

Each ECHO will be transmitted electronically to a designated central imaging laboratory for interpretation and analysis. The ECHO done at screening must be confirmed by the central ECHO laboratory prior to enrollment. If an ECHO demonstrates imaging quality issues, a limited repeat ECHO will be repeated at the discretion of the central ECHO laboratory.

The table below presents the ECHO measurements at each time point.

Study EZDB Echocardiogram Parameters

Echocardiogram Parameter	
2D echo	• LV volumes<br>○ LVEDVI<br>○ LVESVI<br>• LVEF<br>• LA volumes<br>○ LAEDVI<br>○ LAESVI<br>• LA emptying fraction<br>• LVM (LVMI)
Doppler	• E/A<br>• RVSP<br>• TR
Tissue Doppler	• E/e
Color Doppler (Qualitative Measurements only)	• Mitral regurgitant severity<br>• Aortic regurgitant severity<br>• Tricuspid regurgitant severity
Speckle tracking	• LARS

Abbreviations: LA = left atrium; LAEDVI = left atrial end-diastolic volume index; LAESVI = left atrial end-systolic volume index; LARS = left atrial reservoir strain; LV = left ventricle; LVGLS = left ventricular global longitudinal strain; LVEDVI = left ventricular end-diastolic volume index; LVEF = left ventricular ejection fraction; LVESVI = Left ventricular end-systolic volume index; LVM = left ventricular mass; LVMI = left ventricular mass index; RVSP = right ventricular systolic pressure; TR = tricuspid regurgitation.

Final

Approved on 09 Apr 2024 GMT
Downloaded by Flora Sam on 09 Apr 2024

8.1.2.3. Patient-Reported Outcomes

The self-administered questionnaires will be translated into the native language of the region and administered at the site during the designated visits in the SoA.

The questionnaires should be completed before the participant has discussed their medical condition or progress in the study with the investigator or study personnel. Preferred administration order of these questionnaires is:

1. Most Bothersome Symptom
2. Dyspnea NRS
3. Edema NRS
4. Fatigue NRS
5. PGIS-HF Overall Health
6. PGIC-HF Overall Health
7. PGIS-HF Symptom Severity
8. PGIC-HF Symptom Severity, and
9. KCCQ

8.1.2.3.1. Most Bothersome Symptom

The Most Bothersome Symptom question is a single-item PRO designed to capture information on the symptom that is perceived by a patient as being the HF-related symptom that bothers them the most. Patients can choose dyspnea, edema, or fatigue.

8.1.2.3.2. Dyspnea Numeric Rating Scale

The Dyspnea NRS is a single-item PRO designed to capture information on self-reported severity of the worst episode(s) of difficulty breathing or shortness of breath (dyspnea) in the past 2 weeks.

8.1.2.3.3. Edema Numeric Rating Scale

The Edema NRS is a single-item PRO designed to capture information on self-reported severity of worst swelling (edema) in the ankles, feet, or legs in the past 2 weeks.

8.1.2.3.4. Fatigue Numeric Rating Scale

The Fatigue NRS is a single-item PRO designed to capture information on self-reported severity of worst tiredness or lack of energy (fatigue) in the past 2 weeks.

8.1.2.3.5. Patient Global Impression Scales

In addition to the instruments above, study participants will complete 2 PGIS-HF and 2 PGIC-HF scales to obtain a global assessment of patient's overall health status and HF symptom severity. The PGIS-HF and PGIC-HF scales will also serve as anchors in anchor-based methods for defining a range of reasonable meaningful within-patient change on the Dyspnea NRS, Edema NRS, and Fatigue NRS.

Final

Approved on 09 Apr 2024 GMT
Downloaded by Flora Sam on 09 Apr 2024

PGIS- HF Overall Health

The PGIS-HF Overall Health was specifically developed for this study. This is a participant-rated assessment of their overall health "in the past 2 weeks" and is rated on a 5-point scale ranging from "Excellent" to "Poor."

PGIS- HF Symptom Severity

The PGIS-HF Symptom Severity was specifically developed for this study. This is a participant-rated assessment of the overall severity of their HF symptoms "in the past 2 weeks" and is rated on a 5-point scale ranging from "No symptoms" to "Very severe."

PGIC- HF Overall Health

The PGIC-HF Overall Health was specifically developed for this study. This is a participant-rated assessment of the overall change in their health since they started taking the study medication and is rated on a 5-point scale ranging from "Much better" to "Much worse."

PGIC- HF Symptom Severity

The PGIC-HF Symptom Severity was specifically developed for this study. This is a participant-rated assessment of the overall change in their HF symptom severity since they started taking the study medication and is rated on a 5-point scale ranging from "Much better" to "Much worse."

8.1.2.3.6. Kansas City Cardiomyopathy Questionnaire

The KCCQ is a 23-item, participant self-administered questionnaire that assesses impacts of HF "over the past 2 weeks" on the following 7 domains (Green et al. 2000; Joseph et al. 2013):

- Physical Limitation (6 items)
- Symptom Stability (1 item)
- Symptom Frequency (4 items)
- Symptom Burden (3 items)
- Self-Efficacy (2 items)
- Quality of Life (3 items), and
- Social Limitation (4 items).

Each of the 23 individual items are answered on Likert scales of varying lengths (5-point, 6-point, or 7-point scales). Domain scores are obtained by averaging the associated individual items and transforming the score to a 0 to 100 range. Higher scores indicate better health status. Summary scores are obtained by combining select domain scores:

- Total Symptom Score: mean of the Symptom Frequency and Symptom Burden scores
- Clinical Summary Score: mean of the Physical Limitation and Total Symptom scores, and
- Overall Summary Score: mean of the Physical Limitation, Total Symptom, Quality of Life, and Social Limitation scores.

The Total Symptom Score, Clinical Summary Score, and Overall Summary Score will be used for the tertiary endpoints.

Final

Approved on 09 Apr 2024 GMT
Downloaded by Flora Sam on 09 Apr 2024

8.2. Safety Assessments

Planned time points for all safety assessments are provided in the SoA.

8.2.1. Physical Examinations

A complete physical examination will include, at a minimum, assessments of the cardiovascular, respiratory, gastrointestinal, and neurological systems. Height and weight will also be measured and recorded, as specified in SoA.

Additional assessments should include clinical signs and symptoms related to HF, and ISRs.

8.2.2. Vital Signs

BP (supine and standing), pulse rate, respiratory rate, and temperature will be measured when specified in the SoA and as clinically indicated. Additional vital signs may be measured during study visits if warranted, as determined by the investigator.

Vital signs should be measured after participant has been supine at least 5 minutes, before obtaining an ECG tracing, collection of blood samples for laboratory testing, and prior to study drug administration when feasible. For orthostatic measurements, participants should be supine for at least 5 minutes and stand for at least 3 minutes. Unscheduled orthostatic vital signs should be assessed, if possible, during any AE of dizziness or posture-induced symptoms. BP may be remeasured within 30 minutes at investigator discretion.

Final

8.2.3. Electrocardiograms

For each participant, a 12-lead digital ECG will be collected as replicates as indicated in the SoA. ECGs must be recorded before collecting any blood samples. Participants must be supine for approximately 5 to 10 minutes before ECG collection and remain supine but awake during ECG collection. Consecutive replicate ECGs will be obtained at approximately 1-minute intervals. ECGs may be obtained at additional times, when deemed clinically necessary.

At time points that require triplicate ECG, 3 individual ECG tracings should be obtained as closely as possible in succession, but no more than 2 minutes apart.

If triplicate 12-lead ECGs and PK sampling are scheduled for the same visit, PK sample should be collected within 30 minutes after triplicate ECG.

ECGs will be interpreted by a qualified physician (the investigator or qualified designee) at the site as soon after the time of ECG collection as possible, and ideally while the participant is still present, to determine whether the participant meets entry criteria at the relevant visit(s) and for immediate participant management, should any clinically relevant findings be identified.

If a clinically significant quantitative or qualitative change from baseline is identified after enrollment, the investigator will assess the participant for symptoms (for example, palpitations, near syncope, syncope) to determine whether the participant can continue in the study. The investigator or qualified designee is responsible for determining if any change in participant management is needed and must document their review of the ECG printed at the time of evaluation from at least 1 of the replicate ECGs from each time point.

Approved on 09_Apr 2024 GMT
Downloaded by Flora Sam on 09 Apr 2024

Digital ECGs will be electronically transmitted to a Lilly-designated central ECG laboratory. The central ECG laboratory will perform a basic quality control check (for example, demographics and study details) then store the ECGs in a database. At a future time, the stored ECG data may be overread at the central ECG laboratory for further evaluation of machine-read measurements or to meet regulatory requirements.

The machine-read ECG intervals and heart rate may be used for data analysis and report writing purposes unless a cardiologist overread of the ECGs is conducted prior to completion of the final study report, in which case the overread data would be used.

8.2.4. Clinical Safety Laboratory Tests

See Section 10.2 for the list of clinical laboratory tests to be performed and the SoA for the timing and frequency.

The investigator must review the laboratory results, document this review, and report any clinically relevant changes occurring during the study as an AE. The laboratory results must be retained with source documents unless a Source Document Agreement or comparable document cites an electronic location that accommodates the expected retention duration. Clinically significant abnormal laboratory findings are those which are not associated with the underlying disease, unless judged by the investigator to be more severe than expected for the participant's condition.

All laboratory tests with values considered clinically significantly abnormal during participation in the study should be repeated until the values return to normal or baseline or are no longer considered clinically significant by the investigator or medical monitor.

- If such values do not return to normal/baseline within a period of time judged reasonable by the investigator, the etiology should be identified and the sponsor notified.
- All protocol-required laboratory assessments, as defined in Section 10.2, must be conducted in accordance with the SoA and standard collection requirements.

If laboratory values from non-protocol specified laboratory assessments performed at an investigator-designated local laboratory require a change in participant management or are considered clinically significant by the investigator (for example, SAE or AE or dose modification), then report the information as an AE.

8.2.5. Pregnancy Testing

Pregnancy testing will not be performed as only women not of childbearing potential may participate in this study.

Final

Approved on 09 Apr 2024 GMT
Downloaded by Flora Sam on 09 Apr 2024

8.3. Adverse Events, Serious Adverse Events, and Product Complaints

The definitions of the following events can be found in Section 10.3:

- AEs
- SAEs
- PCs

These events will be reported by the participant or, when appropriate, by a caregiver, surrogate, or the participant's legally authorized representative.

The investigator and any qualified designees are responsible for detecting, documenting, and recording events that meet these definitions and remain responsible for following up events that are serious, considered related to the study intervention or study procedures, or that caused the participant to discontinue the study intervention or study (see Section 7).

Care will be taken not to introduce bias when detecting events. Open-ended and non-leading verbal questioning of the participant is the preferred method to inquire about event occurrences.

After the initial report, the investigator is required to proactively follow each participant at subsequent visits or contacts. All SAEs and safety topic of special interest as defined in Section 8.3.7 will be followed until resolution, stabilization, the event is otherwise explained, or the participant is lost to follow-up, as defined in Section 7.3. For PCs, the investigator is responsible for ensuring that follow-up includes any supplemental investigations as indicated to elucidate the nature and/or causality. Further information on follow-up procedures is provided in Section 10.3.

Final

Approved on 09 Apr 2024 GMT
Downloaded by Flora Sam on 09 Apr 2024

8.3.1. Timing and Mechanism for Collecting Events

This table describes the timing, deadlines, and mechanism for collecting events.

Event	Collection Start	Collection Stop	Timing for Reporting to Sponsor or Designee	Mechanism for Reporting	Back-up Method of Reporting
**Adverse Event**					
AE	Signing of the ICF	Participation in study has ended	As soon as possible upon site awareness	AE CRF	N/A
**Serious Adverse Event**					
SAE and SAE updates – prior to start of study intervention **and** deemed reasonably possibly related to study procedures	Signing of the ICF	Start of intervention	Within 24 hr of awareness	SAE CRF	SAE paper form
SAE and SAE updates – after start of study intervention	Start of intervention	Participation in study has ended	Within 24 hr of awareness	SAE CRF	SAE paper form
SAE[a] – after participant's study participation has ended **and** the investigator becomes aware	After participant's study participation has ended	N/A	Promptly	SAE paper form	N/A
**Pregnancy**					
Pregnancy in female participants and female partners of male participants	After the start of study intervention	135 days after the last dose	Within 24 hr (see Section 8.3.2)	Pregnancy CRF	Pregnancy paper form

Final

Approved on 09 Apr 2024 GMT
Downloaded by Flora Sam on 09 Apr 2024

Event	Collection Start	Collection Stop	Timing for Reporting to Sponsor or Designee	Mechanism for Reporting	Back-up Method of Reporting
**Product Complaints**					
PC associated with an SAE or might have led to an SAE	Start of study intervention	End of study intervention	Within 24 hr of awareness	PC form	N/A
PC not associated with an SAE	Start of study intervention	End of study intervention	Within 1 business day of awareness	PC form	N/A
Updated PC information	—	—	As soon as possible upon site awareness	Originally completed PC form with all changes signed and dated by the investigator	N/A
PC (if investigator becomes aware)	Participation in study has ended	N/A	Promptly	PC form	

Abbreviations: AE = adverse event; CRF = case report form; ICF = informed consent form; N/A = not applicable; PC = product complaint; SAE = serious adverse event.

[a] SAEs should not be reported unless the investigator deems them to be possibly related to study treatment or study participation.

8.3.2. Pregnancy

Collection of pregnancy information

Male participants with partners who become pregnant

The investigator will attempt to collect pregnancy information on any male participant's female partner who becomes pregnant while the male participant is in this study. This applies only to male participants who receive study intervention.

After learning of a pregnancy in the female partner of a study participant, the investigator

- will obtain a consent to release information from the pregnant female partner directly, and
- within 24 hours after obtaining this consent will record pregnancy information on the appropriate form and submit it to the sponsor.

The female partner will also be followed to determine the outcome of the pregnancy. Information on the status of the mother and child will be forwarded to the sponsor. Generally, the follow-up will be no longer than 6 to 8 weeks following the estimated delivery date. Any

Final

Approved on 09 Apr 2024 GMT
Downloaded by Flora Sam on 09 Apr 2024

termination of the pregnancy will be reported regardless of gestational age, fetal status (presence or absence of anomalies) or indication for the procedure.

Female participants who become pregnant

The investigator will collect pregnancy information on any female participant who becomes pregnant while participating in this study. The initial information will be recorded on the appropriate form and submitted to the sponsor within 24 hours of learning of a participant's pregnancy.

The participant will be followed to determine the outcome of the pregnancy. The investigator will collect follow-up information on the participant and the neonate and the information will be forwarded to the sponsor. Generally, follow-up will not be required for longer than 6 to 8 weeks beyond the estimated delivery date. Any termination of pregnancy will be reported, regardless of gestational age, fetal status (presence or absence of anomalies) or indication for the procedure.

While pregnancy itself is not considered to be an AE or SAE, any pregnancy complication or elective termination of a pregnancy for medical reasons will be reported as an AE or SAE.

A spontaneous abortion (occurring at <20 weeks gestational age) or still birth (occurring at ≥20 weeks gestational age) is always considered to be an SAE and will be reported as such.

Any poststudy pregnancy related SAE considered reasonably related to the study intervention by the investigator will be reported to the sponsor as described in protocol Section 8.3.1. While the investigator is not obligated to actively seek this information in former study participants, they may learn of an SAE through spontaneous reporting.

Any female participant who becomes pregnant while participating in the study will discontinue study intervention. If the participant is discontinued from the study, follow the standard discontinuation process and continue directly to the follow-up phase. The follow up on the pregnancy outcome should continue independent of intervention or study discontinuation.

8.3.3. Injection-Site Reactions

Symptoms of a local ISR will be assessed through spontaneous ISR reporting.

If an ISR is reported by a participant or site staff, the ISR CRF will be used to capture additional information about this reaction (for example, injection-site pain, degree and area of erythema, induration, pruritis, and edema).

At the time of ISR reporting, collect an immunogenicity and PK sample.

8.3.4. Hypersensitivity Reactions

Many drugs, including oral agents and biologic agents, carry the risk of systemic hypersensitivity reactions. If such a reaction occurs, additional data should be provided to the sponsor in the designated AE CRFs.

Sites should have appropriately trained medical staff and appropriate medical equipment available when study participants are receiving study intervention. It is recommended that participants who experience a systemic hypersensitivity reaction be treated per national and international guidelines.

Final

Approved on 09 Apr 2024 GMT
Downloaded by Flora Sam on 09 Apr 2024

In the case of a suspected systemic hypersensitivity event, additional blood samples should be collected as described in Section 10.2.1. Laboratory results are provided to the sponsor via the central laboratory.

8.3.5. Hepatic Safety Monitoring, Evaluation, and Criteria for Study Intervention Interruption or Discontinuation

The following tables summarize actions to take based on abnormal hepatic laboratory or clinical changes.

Participants with normal or near-normal baseline (ALT and/or AST or ALP <1.5x ULN)

If this laboratory value is observed…	Then…		
	Initiate or continue close hepatic monitoring	Initiate comprehensive evaluation	Interrupt or discontinue study intervention
ALT or AST ≥3x ULN	X		
ALP ≥2x ULN	X		
TBL ≥2x ULN[a]	X		
ALT or AST ≥5x ULN	X	X	
ALP ≥2.5x ULN	X	X	
ALT or AST ≥3x ULN with hepatic signs or symptoms[b]	X	X	X
ALT or AST ≥5x ULN for more than 2 weeks	X	X	X
ALT or AST ≥8x ULN	X	X	X
ALT or AST ≥3x ULN and TBL ≥2x ULN[a] or INR ≥1.5	X	X	X
ALP ≥3x ULN	X	X	X
ALP ≥2.5x ULN and TBL ≥2x ULN[a]	X	X	X
ALP ≥2.5x ULN with hepatic signs or symptoms[b]	X	X	X

Abbreviations: ALT = alanine transaminase; ALP = alkaline phosphatase; AST = aspartate aminotransferase; INR = international normalized ratio; TBL = total bilirubin; ULN = upper limit of normal.

[a] In participants with Gilbert's syndrome, the threshold for TBL may be higher.

[b] Examples of hepatic signs or symptoms: severe fatigue, nausea, vomiting, right upper quadrant abdominal pain, fever, rash, and/or eosinophilia >5%.

Final

Approved on 09 Apr 2024 GMT
Downloaded by Flora Sam on 09 Apr 2024

Participants with elevated baseline (ALT, AST, or ALP ≥1.5x ULN)

If this laboratory value is observed…	Then…		
	Initiate or continue close hepatic monitoring	Initiate comprehensive evaluation	Interrupt or discontinue study intervention
ALT or AST ≥2x baseline	X		
ALP ≥2x baseline	X		
TBL ≥2x ULN[a]	X		
ALT or AST ≥3x baseline or ≥250 U/L (whichever occurs first)	X	X	
ALP ≥2.5x baseline	X	X	
ALT or AST ≥2x baseline or ≥250 U/L (whichever occurs first) with hepatic signs or symptoms[b]	X	X	X
ALT or AST ≥3x baseline or ≥250 U/L (whichever occurs first) for more than 2 weeks	X	X	X
ALT or AST ≥4x baseline or ≥400 U/L (whichever occurs first)	X	X	X
ALT or AST ≥2x baseline or ≥250 U/L (whichever occurs first) and TBL ≥2x ULN[a] or INR ≥1.5	X	X	X
ALP ≥3x baseline	X	X	X
ALP ≥2.5x baseline and TBL ≥2x ULN[a]	X	X	X
ALP ≥2.5x baseline with hepatic signs or symptoms[b]	X	X	X

Final

Abbreviations: ALT = alanine transaminase; ALP = alkaline phosphatase; AST = aspartate aminotransferase; TBL = total bilirubin; ULN = upper limit of normal.

[a] In participants with Gilbert's syndrome, the threshold for TBL may be higher.

[b] Examples of hepatic signs or symptoms: severe fatigue, nausea, vomiting, right upper quadrant abdominal pain, fever, rash, and/or eosinophilia >5%.

8.3.5.1. Close Hepatic Monitoring

If a participant develops any 1 of these changes, initiate close hepatic monitoring:

Participants with normal or near-normal baseline liver enzymes (ALT, AST, or ALP <1.5x ULN)	Participants with elevated baseline liver enzymes (ALT, AST or ALP ≥1.5x ULN)
ALT or AST ≥3x ULN **or**	ALT or AST ≥2x baseline **or**
ALP ≥2x ULN **or**	ALP ≥2x baseline **or**
TBL ≥2x ULN[a]	TBL ≥2x ULN [a]

Abbreviations: ALT = alanine transaminase; ALP = alkaline phosphatase; AST = aspartate aminotransferase; TBL = total bilirubin; ULN = upper limit of normal.

[a] In participants with Gilbert's syndrome, the threshold for TBL may be higher.

Approved on 09 Apr 2024 GMT
Downloaded by Flora Sam on 09 Apr 2024

Close hepatic monitoring should include these actions:

- Laboratory tests (Section 10.6), including ALT, AST, ALP, TBL, direct bilirubin, gamma-glutamyl transferase, creatine kinase, and complete blood count with differential, should be checked within 48 to 72 hours of the detection of elevated liver tests to confirm the abnormality and to determine if it is increasing or decreasing.
- If the abnormality persists, clinical and laboratory monitoring should continue at a frequency of 2 to 3 times weekly until levels normalize or return to approximate baseline values.
- In addition to laboratory tests, basic evaluation for possible causes of abnormal liver tests should be initiated by the investigator in consultation with the Lilly-designated medical monitor. At a minimum, this evaluation should include physical examination and a thorough medical history, including
  - current symptoms
  - recent illnesses, for example, systemic infection, hypotension, or seizures
  - recent travel
  - concomitant medications, including over-the-counter
  - herbal and dietary supplements
  - history of alcohol drinking, and
  - other substance abuse.

Final

8.3.5.2. Comprehensive Hepatic Evaluation

If a participant develops any 1 of the following laboratory or clinical changes, initiate a comprehensive hepatic evaluation:

Participants with normal or near-normal baseline liver enzymes (ALT, AST, or ALP <1.5x ULN)	Participants with elevated baseline liver enzymes (ALT, AST, or ALP ≥1.5x ULN)
ALT or AST ≥5x ULN **or**	ALT or AST ≥3x baseline or ≥250 U/L (whichever occurs first) **or**
ALP ≥2.5x ULN **or**	ALP ≥2.5x baseline **or**
ALT or AST ≥3x ULN with hepatic signs or symptoms[a] **or**	ALT or AST ≥2x baseline or ≥250 U/L (whichever occurs first) with hepatic signs or symptoms[a] **or**
ALT or AST ≥5x ULN for more than 2 weeks **or**	ALT or AST ≥3x baseline or ≥250 U/L (whichever occurs first) for more than 2 weeks **or**
ALT or AST ≥8x ULN **or**	ALT or AST ≥4x baseline or ≥400 U/L (whichever occurs first) **or**
ALT or AST ≥3x ULN and TBL ≥2x ULN[b] or INR ≥1.5	ALT or AST ≥2x baseline or ≥250 U/L (whichever occurs first) and TBL ≥2x ULN[b] or INR ≥1.5

Abbreviations: ALT = alanine transaminase; ALP = alkaline phosphatase; AST = aspartate aminotransferase; INR = international normalized ratio; TBL = total bilirubin; ULN = upper limit of normal.

[a] Examples of hepatic signs or symptoms: severe fatigue, nausea, vomiting, right upper quadrant abdominal pain, fever, rash, and/or eosinophilia >5%.

[b] In participants with Gilbert's syndrome, the threshold for TBL may be higher.

Approved on 09 Apr 2024 GMT
Downloaded by Flora Sam on 09 Apr 2024

Comprehensive hepatic evaluation should include these actions:

- At a minimum, comprehensive hepatic evaluation should include
  - physical examination and a thorough medical history, as outlined in Sections 1.3 and 8.2.1, and
  - tests for
    - prothrombin time - international normalized ratio
    - viral hepatitis A, B, C, and E
    - autoimmune hepatitis, and
    - an abdominal imaging study (for example, ultrasound or CT scan).
- Based on the participant's history and initial results, further testing should be considered in consultation with the Lilly-designated medical monitor, including tests for
  - hepatitis D virus
  - cytomegalovirus
  - Epstein-Barr virus
  - acetaminophen levels
  - acetaminophen protein adducts
  - urine toxicology screen
  - Wilson's disease
  - blood alcohol levels
  - urinary ethyl glucuronide, and
  - blood phosphatidylethanol.
- Based on the circumstances and the investigator's assessment of the participant's clinical condition, the investigator should consider referring the participant for a hepatologist or gastroenterologist consultation, and additional tests, including magnetic resonance cholangiopancreatography, endoscopic retrograde cholangiopancreatography, cardiac echocardiogram, or a liver biopsy.
- Clinical and laboratory monitoring should continue at a frequency of 1 to 3 times weekly until levels normalize or return to approximate baseline values.

All the medical information and tests results related to the hepatic monitoring and comprehensive hepatic evaluation should be collected and recorded in a hepatic safety CRF.

8.3.5.3. Study Intervention Interruption or Discontinuation

If a participant develops any 1 of the following laboratory or clinical changes, interrupt the study intervention and continue close monitoring and comprehensive hepatic evaluation as described in Sections 8.3.5.1 and 8.3.5.2.

Final

Approved on 09 Apr 2024 GMT
Downloaded by Flora Sam on 09 Apr 2024

**Participants with normal or near-normal baseline liver enzymes (ALT and/or AST and/or ALP <1.5x ULN)**	**Participants with elevated baseline liver enzymes (ALT and/or AST and/or ALP ≥1.5x ULN)**
ALT or AST ≥3x ULN [a] with hepatic signs or symptoms[a] **or**	ALT or AST ≥2x baseline or ≥250 U/L (whichever occurs first) with hepatic signs or symptoms[a] **or**
ALT or AST ≥5x ULN [a] for more than 2 weeks **or**	ALT or AST ≥3x baseline or ≥250 U/L (whichever occurs first) for more than 2 weeks **or**
ALT or AST ≥8x ULN [a] **or**	ALT or AST ≥4x baseline or ≥400 U/L (whichever occurs first) **or**
ALT or AST ≥3x ULN [a] and TBL ≥2x ULN or INR ≥1.5 **or**	ALT or AST ≥2x baseline or ≥250 U/L (whichever occurs first) and TBL ≥2x ULN **or**
ALP ≥3x ULN [a] **or**	ALP ≥3x baseline **or**
ALP ≥2.5x ULN [a] and TBL ≥2x ULN[b] **or**	ALP ≥2.5x baseline and TBL ≥2x ULN[b] **or**
ALP ≥2.5x ULN [a] with hepatic signs or symptoms[a]	ALP ≥2.5x baseline with hepatic signs or symptoms[a]

Abbreviations: ALT = alanine transaminase; ALP = alkaline phosphatase; AST = aspartate aminotransferase; INR = international normalized ratio; TBL = total bilirubin; ULN = upper limit of normal.

[a] Examples of hepatic signs or symptoms: severe fatigue, nausea, vomiting, right upper quadrant abdominal pain, fever, rash, and/or eosinophilia >5%.

[b] In participants with Gilbert's syndrome, the threshold for TBL may be higher.

Final

Interruption or discontinuation of study intervention should include these actions:

- While the participant is not receiving the study intervention, clinical and laboratory monitoring should continue at a frequency of 1 to 3 times weekly until liver tests normalize or return to approximate baseline values.
- If the hepatic event continues past the anticipated end of the study (that is, data lock), the investigator should consult with the Lilly-designated medical monitor to determine the need for further data collection beyond the end date of the study (that is, data lock date).
- All the medical information and tests results related to the close hepatic monitoring and comprehensive hepatic evaluation should be collected and recorded in a hepatic safety CRF.

Resumption of the study intervention after interruption for a hepatic reason can be considered only in consultation with the Lilly-designated medical monitor and only if the liver test results returned to near baseline and if a self-limited, non-study drug etiology is identified. Otherwise, the study intervention should be permanently discontinued.

8.3.6. Major Adverse Cardiovascular Events (MACE)

Death and nonfatal cardiovascular AEs will be adjudicated by a committee of physicians external to Lilly with cardiology expertise. This committee will be blinded to treatment assignment.

The nonfatal cardiovascular AEs to be adjudicated include

- myocardial infarction
- hospitalization for unstable angina

Approved on 09 Apr 2024 GMT
Downloaded by Flora Sam on 09 Apr 2024

- hospitalization for HF
- coronary interventions, such as coronary artery bypass graft or percutaneous coronary intervention
- cerebrovascular events, including cerebrovascular accident (stroke) and transient ischemic attack, and
- urgent HF visits

8.3.7. Safety Topics of Special Interest

Each occurrence of the below events will be recorded as a separate AE in the CRF. For each event assessment of severity, duration (actual date, time of onset, and end times), and investigator's opinion of relatedness to study intervention and protocol procedure will be captured.

8.3.7.1. Vaginal Bleed

If a participant reports vaginal bleeding while being treated with study intervention, the investigator should discontinue study treatment and refer the participant for a gynecological assessment. At the discretion of the local physician, this follow-up examination may include a Pap smear, endometrial biopsy, or transvaginal ultrasound as the initial test for evaluating the endometrium. The gynecological report should be sent to sponsor medical monitor.

8.3.7.2. Breast Screening

Compliance with routine breast screening is recommended per local guidelines. Abnormal finding during breast screening per local guidelines will be recorded as AE.

8.3.7.3. Orthostatic Hypotension

Symptomatic hypotension-related events (for example, syncope, dizziness, falls, clinically significant drop in BP, etc.)

Orthostatic hypotension: A sustained reduction in systolic blood pressure of at least 20 mmHg within 3 minutes of standing after being supine for 5 minutes.

8.4. Pharmacokinetics

Venous blood samples will be collected from all study participants for measurement of plasma concentrations of LY3540378 as specified in the SoA.

A maximum of 3 samples may be collected at additional time points during the study if warranted and agreed upon between the investigator and the sponsor. The timing of sampling may be altered during the course of the study based on newly available data, for example, to obtain data closer to the time of peak plasma concentrations, to ensure appropriate safety monitoring.

Instructions for the collection and handling of biological samples will be provided by the sponsor. The actual date and time (24-hour clock time) of each LY3540378 sample and dose administered must be recorded.

Final

Approved on 09 Apr 2024 GMT
Downloaded by Flora Sam on 09 Apr 2024

Samples will be used to evaluate the PK of LY3540378. Samples collected for analyses of LY3540378 plasma concentrations may also be used to evaluate safety or efficacy aspects related to concerns arising during or after the study.

Genetic analyses will not be performed on these plasma samples.

Drug concentration information that would unblind the study will not be reported to investigative sites or blinded personnel.

8.4.1. Bioanalysis

Samples will be analyzed at a laboratory approved by the sponsor and stored at a facility designated by the sponsor. Concentrations of LY3540378 will be assayed using a validated liquid chromatography mass spectrometry method. Analyses of samples collected from placebo-treated subjects are not planned.

Bioanalytical samples collected to measure study intervention concentrations will be retained for a maximum of 1 year following the last participant visit for the study. During this time, samples remaining after the bioanalyses may be used for exploratory analyses such as metabolism work, protein binding, and/or bioanalytical method cross-validation.

8.5. Pharmacodynamics

Pharmacodynamic parameters are described in Section 8.1.2.1.

8.6. Genetics

A blood sample for DNA isolation may be collected from participants.

See Section 10.5 for Information regarding genetic research and Section 10.1.12 for details about sample retention and custody.

Additionally, HLA Class II testing may be conducted in order to define the immunophenotypic characteristics that might be associated with incidence of injection site reactions observed following LY3540378 administration.

8.7. Biomarkers

Serum, plasma, and urine samples will be used for exploratory biomarker research, where local regulations allow.

See Clinical Laboratory Tests in Section 10.2, and the SoA for sample collection information.

Samples will be used for research on the drug target, disease process, variable response to LY3540378, pathways associated with HF, mechanisms of action of LY3540378 or research methods, or in validating diagnostic tools or assay(s) related to HF. Samples may be used for research to develop methods, assays, prognostics, and/or companion diagnostics related to the intervention target, disease state, pathways associated with disease, and/or the mechanism of action of the study intervention.

Sample retention is described in Section 10.1.12.

Final

Approved on 09 Apr 2024 GMT
Downloaded by Flora Sam on 09 Apr 2024

8.8. Immunogenicity Assessments

At the visits and times specified in the SoA (Section 1.3), venous blood samples will be collected to determine antibody production against LY3540378. Antibodies may be further characterized for their ability to neutralize the activity of LY3540378. To interpret the results of immunogenicity, a venous blood sample will be collected at the same time points to determine the serum concentrations of LY3540378. All samples for immunogenicity should be taken predose when applicable and possible. Upon assay validation, ADAs may be characterized; treatment-emergent ADAs are defined in Section 9.3.7.

If the immunogenicity sample at the last scheduled assessment or discontinuation visit indicates TE-ADA, additional samples may be taken until the signal returns to baseline ,that is, no longer indicates TE-ADA or for up to 1 year after last dose.

Immunogenicity will be assessed by a validated assay designed to detect ADAs in the presence of LY3540378 at a laboratory approved by the sponsor. The purpose of retention, the maximum duration of retention, and facility for long-term storage of samples is described in Section 10.1.12. Samples may also be used for development and control of an immunogenicity assay.

8.9. Medical Resource Utilization and Health Economics

Health economics or medical resource utilization and health economics parameters are not evaluated in this study

Final

Approved on 09_Apr 2024 GMT
Downloaded by Flora Sam on 09 Apr 2024

9. Statistical Considerations

9.1. Statistical Hypotheses

The study hypothesis for the primary objective is that LY3540378 administered SC QW is superior to placebo for change from baseline in LARS at Week 26 in participants with worsening chronic HFpEF.

9.1.1. Multiplicity Adjustment

Treatment comparisons will be performed for the primary objective at the full significance level of 0.05. No multiplicity adjustments will be made for the analysis of primary, secondary, and tertiary objectives.

9.2. Analyses Sets

This table defines the analysis population and datasets for the purposes of analysis.

Participant Analysis Set	Description
Screened	All participants who signed informed consent.
Randomized	All participants who are randomly assigned to a treatment arm.
Efficacy Analysis Set (EAS)	Data obtained during the treatment period from all randomly assigned participants who are exposed to at least 1 dose of intervention. Excludes data after permanent discontinuation of intervention. Participants will be included in the treatment group to which they were randomly assigned.
Full Analysis Set (FAS)	Data obtained during the treatment period from all randomly assigned participants who are exposed to at least 1 dose of intervention, regardless of adherence to intervention. Participants will be included in the treatment group to which they were randomly assigned.
Safety Analysis Set (SS)	Data obtained during the treatment period plus safety follow-up from all randomly assigned participants who are exposed to at least 1 dose of intervention, regardless of adherence to intervention. Participants will be included in the treatment group to which they were randomly assigned.

Final

9.3. Statistical Analyses

9.3.1. General Considerations

Statistical analysis of this study will be the responsibility of Lilly or its designee.

Any change to the data analysis methods described in the protocol will require an amendment ONLY if it changes a principal feature of the protocol. Any other change to the data analysis methods described in the protocol, and the justification for making the change, will be

Approved on 09 Apr 2024 GMT
Downloaded by Flora Sam on 09 Apr 2024

described in the CSR. Additional exploratory analyses of the data will be conducted as deemed appropriate.

Baseline is defined as the last non-missing measurement recorded on or before the randomization visit, prior to first dose of intervention, unless otherwise specified.

Handling of missing, unused, and spurious data are addressed prospectively in the overall statistical methods described in the protocol and in the SAP, where appropriate. Adjustments to the planned analyses are described in the final CSR.

Primary estimand of interest and efficacy assessment

The primary estimand is a precise definition of the treatment effect to be estimated. The primary estimand of interest is comparing efficacy of LY3540378 doses to placebo and is named the "efficacy estimand" (Section 3). The efficacy estimand represents the efficacy prior to discontinuation of intervention. The primary efficacy assessment guided by the efficacy estimand will be conducted using the EAS (Section 9.2).

Safety assessments

Unless specified otherwise, safety assessments will be guided by an estimand comparing safety of LY3540378 doses with placebo irrespective of adherence to intervention. Thus, safety analyses will be conducted using the SS (Section 9.2).

Exploratory analyses

The "treatment policy" estimand, which represents the efficacy irrespective of adherence to study intervention, may also be used to compare the efficacy of LY3540378 doses with placebo for primary and secondary endpoints in the exploratory analyses. The analysis guided by the "treatment policy" estimand will use the FAS. Details will be provided in the SAP.

Additional exploratory analyses of the data will be conducted as deemed appropriate.

Analysis models for the comparison among treatment groups

The analysis model for comparisons among treatment groups relative to continuous measurements assessed over time, in addition to the baseline and end of treatment measurements, will be a MMRM with terms:

- treatment
- visit
- treatment-by-visit interaction, and
- baseline measurement

Additional covariates such as stratification factors may be added and will be detailed in SAP.

Logistic regression may be used to examine the treatment difference in binary efficacy outcomes. Fisher's exact test or Pearson's chi-square test may be used to examine the treatment difference in categorical outcomes.

Other statistical methods may be used, as appropriate, and details will be documented in the SAP.

Final

Approved on 09 Apr 2024 GMT
Downloaded by Flora Sam on 09 Apr 2024

9.3.2. Primary Endpoint(s)/Estimand(s) Analysis

The primary estimand analysis is described in Section 3.

The primary efficacy comparison will be based on the contrast between LY3540378 doses of 25, 50, 100 mg; combination of 50 and 100 mg; and placebo for the absolute change of LARS from baseline at screening (V1) to Week 26.

The primary analyses model will be MMRM as described in Section 9.3.1. Treatment comparisons will be performed at the full significance level of 0.05. Additional covariates may be added, and this analysis will be detailed in the SAP.

9.3.3. Secondary Endpoints Analysis

The secondary estimand analysis is described in Section 3.

The efficacy analyses for the secondary endpoints will use the EAS and MMRM analysis described in Section 9.3.1. The clinical measures for secondary endpoints may be log transformed before statistical analyses, if deemed necessary. This analysis will be detailed in SAP.

9.3.4. Tertiary Endpoints Analysis

The analysis will be detailed in SAP.

9.3.5. Safety Analyses

Safety assessments will be guided by an estimand comparing safety of LY3540378 doses with placebo irrespective of adherence to intervention. Thus, safety analyses will be conducted using the SS.

AEs will be coded from the actual term using the MedDRA and reported with preferred terms and system organ class. Selected notable AEs of interest may be reported using high-level terms or Standardized MedDRA Queries, such as orthostatic hypotension (as defined in Section 8.3.7.3).

Summary statistics will be provided for incidence of

- TEAEs
- SAEs
- study discontinuation due to AEs
- intervention discontinuation due to AEs
- deaths, and
- other cardiovascular endpoints.

Counts and proportions of participants experiencing AEs will be reported for each treatment group, and Fisher's exact test will be used to compare the treatment groups.

9.3.5.1. Central Laboratory Measures, Vital Signs, and Electrocardiograms

Central laboratory measures, vital signs, and selected ECG parameters will be summarized for each scheduled visit by

- actual measures at baseline and postbaseline

Final

Approved on 09 Apr 2024 GMT
Downloaded by Flora Sam on 09 Apr 2024

- change from baseline to postbaseline, or
- percent change from baseline to postbaseline.

The analysis details will be provided in the SAP.

9.3.6. Pharmacokinetic and Pharmacodynamic Analyses

A population PK approach using nonlinear mixed-effects modeling will be used to analyze LY3540378 concentration data.

The relationships between LY3540378 dose and/or concentration and selected efficacy (for example, LARS, NT-proBNP, LAEDVI, LAESVI, eGFR, serum creatinine and cystatin-C) and safety (for example, BP) endpoints may be characterized, where applicable. Additionally, the impact of intrinsic and extrinsic factors, such as age, weight, gender, and renal function on PK and/or PD parameters, may be examined as needed.

If ADA titers are detected from immunogenicity testing, then the impact of immunogenicity titers on LY3540378 PK or any relevant efficacy parameters may also be examined. Additional analyses may be conducted if they are deemed appropriate. Details on PK and PK/PD analyses will be provided in the PK/PD analysis plan.

9.3.7. Immunogenicity Assessments

If data from validated immunogenicity assays are available, TE-ADAs may be assessed.

TE-ADAs are defined as

- a titer 2-fold (1 dilution) greater than the minimum required dilution if no ADAs were detected at baseline (treatment-induced ADA), or a 4-fold (2 dilutions) increase in titer compared with baseline if ADAs were detected at baseline (treatment-boosted ADA).

The frequency and percentage of participants with preexisting ADA and who are TE-ADA positive (TE-ADA+) to LY3540378 may be tabulated.

The distribution of titers and frequency of neutralizing antibodies (if assessed) for the TE-ADA+ participants may also be tabulated.

The relationship between the presence of antibodies and PK parameters, efficacy response or safety to LY3540378 may also be assessed. Additional details may be provided in the SAP.

9.3.8. Subgroup Analyses

Subgroup analyses of important factors, such as stratification factor, gender, NYHA class, and other factors to be specified in the SAP, are planned for the key outcomes.

The models used for these analyses will vary depending on the subgroups and the outcome. Other exploratory subgroup analyses may be performed as deemed appropriate.

Details of the modeling will be provided in the SAP.

Final

Approved on 09 Apr 2024 GMT
Downloaded by Flora Sam on 09 Apr 2024

9.4. Interim Analysis

There may be up to 4 interim analyses including primary database lock.

A planned interim analysis may be conducted when between 40% and 80% of the participants complete Week 26 at Visit 24 or discontinue the study. The interim will be for the purpose of internal planning and decision-making and may assess safety, PK, and/or efficacy measures. At the discretion of the sponsor, the prespecified interim analysis may not be conducted. Additional details related to statistical methods will be described in the SAP. If this happens, an AC will be formed to review the interim analyses in an unblinded manner. The details regarding the number of participants and type of analysis will be provided in the AC charter and in the unblinding plan. Information that may unblind the study during the analyses will not be reported to study sites or blinded study team members before the study has been unblinded. Study sites will receive information about interim results only if deemed necessary for the safety of the participants. The study will not be stopped based on the efficacy of LY3540378 versus placebo. Therefore, there will be no inflation of the type 1 error rate, and no need to employ an alpha spending function or multiplicity adjustment.

The primary database lock and primary data analysis for study EZDB may occur when all participants have completed 26 weeks (Visit 24) of treatment. The final database lock and final data analysis will occur when all randomized participants have completed the study. Participants and investigators will remain blinded until the completion of the study. If there is no primary database lock, the primary analysis will be based on the final database lock.

Early access to the PK and PD data before the interim and primary database locks may be conducted to allow population PK/PD analysis and model development. If applicable, this early access will be detailed in the Unblinding Plan and the Population PK/PD Analysis Plan.

Final

9.5. Sample Size Determination

The sample size calculation is based on the primary efficacy estimand and its endpoint, change from baseline at Week 26 in LARS.

For participants who joined study before amendment (b), they were randomized 1:1:1:1 to the following intervention groups:

- LY3540378 25 mg SC QW
- LY3540378 50 mg SC QW
- LY3540378 100 mg SC QW, and
- placebo.

For participants who joined study after amendment (b), will be randomized 1:2:2:2 to the above intervention groups.

Up to 456 participants will be randomly assigned to ensure at least 114 participants enrolled in each of 50 mg, 100 mg, and placebo groups. Assuming a 20% dropout rate, this will result in at least 91 completers in each of 50 mg, 100 mg, and placebo group. The number of completers in 25 mg will be approximately 64 to 91, depending on when the amendment (b) is globally implemented.

Approved on 09 Apr 2024 GMT
Downloaded by Flora Sam on 09 Apr 2024

The evaluation of superiority to placebo will be conducted for LY3540378 doses of 50 and 100 mg and combination of 50 and 100 mg. No adjustment for multiplicity will be performed. Assuming a standard deviation of 8.5%, and a 2-sided, alpha level of 0.05, 91 completers for each treatment arms will provide 88% power to detect a treatment difference of 4% for the primary endpoint for LY3540378 50 mg group versus placebo and LY3540378 100 mg group versus placebo, respectively.

Final

Approved on 09 Apr 2024 GMT
Downloaded by Flora Sam on 09 Apr 2024

10. Supporting Documentation and Operational Considerations

10.1. Appendix 1: Regulatory, Ethical, and Study Oversight Considerations

10.1.1. Regulatory and Ethical Considerations

- This study will be conducted in accordance with the protocol and with the following:
  - Consensus ethical principles derived from international guidelines including the Declaration of Helsinki and Council for International Organizations of Medical Sciences International Ethical Guidelines
  - Applicable ICH GCP Guidelines
  - Applicable laws and regulations
- The protocol, protocol amendments, ICF, IB, and other relevant documents (for example, advertisements) must be submitted to an IRB/IEC by the investigator and reviewed and approved by the IRB/IEC before the study is initiated.
- Any amendments to the protocol will require IRB/IEC approval before implementation of changes made to the study design, except for changes necessary to eliminate an immediate hazard to study participants.
- Protocols and any substantial amendments to the protocol will require health authority approval prior to initiation except for changes necessary to eliminate an immediate hazard to study participants.
- The investigator will be responsible for the following:
  - Providing written summaries of the status of the study to the IRB/IEC annually or more frequently in accordance with the requirements, policies, and procedures established by the IRB/IEC
  - Notifying the IRB/IEC of SAEs or other significant safety findings as required by IRB/IEC procedures
  - Providing oversight of study conduct for participants under their responsibility and adherence to requirements of 21 Code of Federal Regulations, ICH guidelines, the IRB/IEC, European regulation 536/2014 for clinical studies (if applicable), and all other applicable local regulations
  - Reporting to the sponsor or designee significant issues related to participant safety, participant rights, or data integrity
- Investigator sites are compensated for participation in the study as detailed in the Clinical Trial Agreement.

10.1.2. Financial Disclosure

Investigators and sub-investigators will provide the sponsor with sufficient, accurate financial information as requested to allow the sponsor to submit complete and accurate financial

Final

Approved on 09_Apr 2024 GMT
Downloaded by Flora Sam on 09 Apr 2024

certification or disclosure statements to the appropriate regulatory authorities. Investigators are responsible for providing information on financial interests during the course of the study and for 1 year after completion of the study.

10.1.3. Informed Consent Process

- The investigator or the investigator's representative will explain the nature of the study, including the risks and benefits, to the participant and answer all questions regarding the study.
- Participants must be informed that their participation is voluntary. Participants will be required to sign a statement of informed consent that meets the requirements of 21 Code of Federal Regulations 50, local regulations, ICH guidelines, privacy and data protection requirements, where applicable, and the IRB/IEC or study center.
- The medical record must include a statement that written informed consent was obtained before the participant was entered in the study and the date the written consent was obtained. The authorized person obtaining the informed consent must also sign the ICF.
- Participants must be reconsented to the most current version of the ICF(s) during their participation in the study.
- A copy of the ICF(s) must be provided to the participant and is kept on file.

Participants who are rescreened are required to sign a new ICF.

10.1.4. Data Protection

Participants will be assigned a unique identifier by the sponsor to protect the participant's personal data. Any participant information, such as records, datasets, or tissue samples that are transferred to the sponsor will contain the identifier only. Participant names or any information which would make the participant identifiable will not be transferred.

The participant must be informed that the participant's personal study-related data will be used by the sponsor in accordance with local data protection law. The level of disclosure must also be explained to the participant who will be required to give consent for their data to be used as described in the informed consent. This is done by the site personnel through the informed consent process.

The participant must be informed through the informed consent by the site personnel that their medical records may be examined by Clinical Quality Assurance auditors or other authorized personnel appointed by the sponsor, by appropriate IRB/IEC members, and by inspectors from regulatory authorities.

The sponsor has processes in place to ensure information security, data integrity, and data protection. These processes address management of data transfer, and prevention and management of unauthorized access, disclosure, dissemination, alteration or loss of information or personal data. These processes include appropriate contingency plan(s) for appropriate and timely response in the event of a data security breach.

Final

Approved on 09 Apr 2024 GMT
Downloaded by Flora Sam on 09 Apr 2024

The transfer of personal data is subject to appropriate safeguards through contractual agreements and processes. The sponsor's processes are compliant with local privacy laws and relevant legislations including the General Data Protection Regulation (GDPR).

10.1.5. Committees Structure

External clinical endpoint committee

An independent clinical endpoint committee, external to Lilly, will be formed to adjudicate MACE, all-cause mortality (CV and non-CV death) and HF events (hospitalization and urgent visit). This committee will be blinded to treatment assignment.

10.1.6. Dissemination of Clinical Study Data

Reports

The sponsor will disclose a summary of study information, including tabular study results, on publicly available websites where required by local law or regulation.

The summary of results will be posted within the time frame specified by local law or regulation. If the study remains ongoing in some countries and a statistical analysis of an incomplete dataset would result in analyses lacking scientific rigor (for example, underpowered) or compromise the integrity of the overall analyses (for example, trial not yet unblinded), the summary of results will be submitted within 1 year after the end of the study globally or as soon as available, whichever is earlier.

Data

The sponsor provides access to all individual participant data collected during the trial, after anonymization, with the exception of PK or genetic data.

Data are available to request 6 months after the indication studied has been approved in the US and EU and after primary publication acceptance, whichever is later. No expiration date of data requests is currently set once data are made available.

Access is provided after a proposal has been approved by an independent review committee identified for this purpose and after receipt of a signed data sharing agreement.

Data and documents, including the study protocol, SAP, CSR, and blank or annotated CRFs, will be provided in a secure data sharing environment for up to 2 years per proposal.

For details on submitting a request, see the instructions provided at www.vivli.org.

10.1.7. Data Quality Assurance

- All participant data relating to the study will be recorded on printed or electronic CRFs unless transmitted to the sponsor or designee electronically (for example, laboratory data). The investigator is responsible for verifying that data entries are accurate and correct by physically or electronically signing the CRF.
- The investigator must maintain accurate documentation (source data) that supports the information entered in the CRF.

Final

Approved on 09 Apr 2024 GMT
Downloaded by Flora Sam on 09 Apr 2024

- The investigator must permit study-related monitoring, audits, IRB/IEC review, and regulatory agency inspections and provide direct access to source data documents.
- Quality tolerance limits will be pre-defined to identify systematic issues that can impact participant safety and/or reliability of study results. These pre-defined parameters will be monitored during the study and important excursions from the quality tolerance limits and remedial actions taken will be summarized in the CSR.
- Monitoring details describing strategy (for example, risk-based initiatives in operations and quality such as risk management and mitigation strategies and analytical risk-based monitoring), methods, responsibilities and requirements, including handling of noncompliance issues and monitoring techniques are provided in the Monitoring Plan.
- The sponsor or designee is responsible for the data management of this study including quality checking of the data.
- The sponsor assumes accountability for actions delegated to other individuals (for example, contract research organizations).
- Study monitors will perform ongoing source data verification to confirm that data transcribed into the CRF by authorized site personnel are accurate, complete, and verifiable from source documents; that the safety and rights of participants are being protected; and that the study is being conducted in accordance with the currently approved protocol and any other study agreements, ICH GCP, and all applicable regulatory requirements.
- Records and documents, including signed ICFs, pertaining to the conduct of this study must be retained by the investigator for the time period outlined in the Clinical Trial Agreement unless local regulations or institutional policies require a longer retention period. No records may be destroyed during the retention period without the written approval of the sponsor. No records may be transferred to another location or party without written notification to the sponsor.
- In addition, sponsor or its representatives will periodically check a sample of the participant data recorded against source documents at the study site. The study may be audited by sponsor or its representatives, and/or regulatory agencies at any time. Investigators will be given notice before an audit occurs.

Final

Data Capture System

The investigator is responsible for ensuring the accuracy, completeness, legibility, and timeliness of the data reported to the sponsor.

An EDC system will be used in this study for the collection of CRF data. The investigator maintains a separate source for the data entered by the investigator or designee into the sponsor-provided EDC system. The investigator is responsible for the identification of any data to be considered source and for the confirmation that data reported are accurate and complete by signing the CRF.

Additionally, study intervention administration data will be collected by the participant, via a paper source document and will be transcribed by the authorized study personnel into the EDC system.

Approved on 09 Apr 2024 GMT
Downloaded by Flora Sam on 09 Apr 2024

Additionally, eCOA data (participant-focused outcome instrument) will be directly recorded by the participant, into an instrument (for example, handheld smart phone or tablet). The eCOA data will serve as the source documentation and the investigator does not maintain a separate written or electronic record of these data.

Data collected via the sponsor-provided data capture system(s) will be stored at third parties. The investigator will have continuous access to the data during the study and until decommissioning of the data capture system(s). Prior to decommissioning, the investigator will receive or access an archival copy of pertinent data for retention.

Data managed by a central vendor, such as laboratory test data, will be stored electronically in the central vendor's database system and reports will be provided to the investigator for review and retention. Data will subsequently be transferred from the central vendor to the sponsor data warehouse.

Data from complaint forms submitted to the sponsor will be encoded and stored in the global PC management system.

10.1.8. Source Documents

- Source documents provide evidence for the existence of the participant and substantiate the integrity of the data collected. Source documents are filed at the investigator's site.
- Data reported on or entered in the CRF and are transcribed from source documents must be consistent with the source documents or the discrepancies must be explained. The investigator may need to request previous medical records or transfer records, depending on the study. Also, current medical records must be available.
- Definition of what constitutes source data can be found in Section 10.1.7.

Final

10.1.9. Study and Site Start and Closure

First act of recruitment

The study start date is the date on which the clinical study will be open for recruitment of participants.

Study or site termination

The sponsor or sponsor's designee reserves the right to close the study site or terminate the study at any time for any reason at the sole discretion of the sponsor. Study sites will be closed upon study completion. A study site is considered closed when all required documents and study supplies have been collected and a study-site closure visit has been performed.

The investigator may initiate study-site closure at any time, provided there is reasonable cause and sufficient notice is given in advance of the intended termination.

Reasons for the early closure of a study site by the sponsor or investigator may include but are not limited to:

For study termination:

- Discontinuation of further study intervention development

For site termination:

Approved on 09 Apr 2024 GMT
Downloaded by Flora Sam on 09 Apr 2024

- Failure of the investigator to comply with the protocol, the requirements of the IRB/IEC or local health authorities, the sponsor's procedures, or GCP guidelines
- Inadequate recruitment (evaluated after a reasonable amount of time) of participants by the investigator
- Total number of participants included earlier than expected.

If the study is prematurely terminated or suspended, the sponsor shall promptly inform the investigators, the IRBs/IECs, the regulatory authorities, and any contract research organization(s) used in the study of the reason for termination or suspension, as specified by the applicable regulatory requirements. The investigator shall promptly inform the participant and should assure appropriate participant therapy and/or follow-up.

10.1.10. Publication Policy

In accordance with the sponsor's publication policy, the results of this study will be submitted for publication by a peer-reviewed journal.

10.1.11. Investigator Information

Researchers with appropriate education, training, and experience, as determined by the sponsor, will participate as investigators in this clinical trial.

10.1.12. Sample Retention

Sample retention enables use of new technologies, response to regulatory questions, and investigation of variable response that may not be observed until later in the development of LY3540378 or after LY3540378 become(s) commercially available.

Sample Type	Custodian	Maximum Retention Period After Last Participant Visit[a]
Exploratory Biomarkers	Sponsor or Designee	7 years
Pharmacokinetics	Sponsor or Designee	1 year
Genetics	Sponsor or Designee	7 years
Immunogenicity	Sponsor or Designee	15 years

[a] Retention periods may differ locally.

Final

Approved on 09 Apr 2024 GMT
Downloaded by Flora Sam on 09 Apr 2024

10.2. Appendix 2: Clinical Laboratory Tests

The tests detailed in the table below will be performed by the Lilly-designated laboratory or by the local laboratory as specified in the table below.

In circumstances where the sponsor approves local laboratory testing in lieu of central laboratory testing (in the table below), the local laboratory must be qualified in accordance with applicable local regulations.

Protocol-specific requirements for inclusion or exclusion of participants are detailed in Section 5 of the protocol.

Additional tests may be performed at any time during the study as determined necessary by the investigator or required by local regulations.

Investigators must document their review of the laboratory safety results.

Laboratory results that could unblind the study will not be reported to investigative sites or other blinded.

Final

Approved on 09 Apr 2024 GMT
Downloaded by Flora Sam on 09 Apr 2024

Clinical Laboratory Tests	Comments
**Hematology**	Assayed by Lilly-designated laboratory.
Hemoglobin	
Hematocrit	
Erythrocyte count (RBCs)	
Mean cell volume	
Mean cell hemoglobin	
Mean cell hemoglobin concentration	
Leukocytes (WBCs)	
Differential	
Percent and Absolute Count of:	
Neutrophils, segmented	
Bands	
Lymphocytes	
Monocytes	
Eosinophils	
Basophils	
Platelets	
Cell morphology (RBC and WBC)	
**Clinical Chemistry**	Assayed by Lilly-designated laboratory.
Sodium	
Potassium	
Chloride	
Bicarbonate	
Total bilirubin	
Direct bilirubin	
ALP	
ALT	
AST	
GGT	
BUN	
Creatinine	Test will be performed locally for screening purposes and by Lilly-designated laboratory for statistical data evaluation. For all other visits, results will be generated by Lilly-designated laboratory.
CK	
Uric acid	
Total protein	
Albumin	
Calcium	
Phosphorus	
Glucose	
**Lipid Panel**	

Final

Approved on 09 Apr 2024 GMT
Downloaded by Flora Sam on 09 Apr 2024

Clinical Laboratory Tests	Comments
HDL-C	
Non-HDL-C	
LDL-C	This value will be calculated. If triglycerides are >400 mg/dL, the direct LDL will be assayed.
VLDL-C	
Cholesterol	
Triglycerides	
**Urinalysis**	Assayed by Lilly-designated laboratory.
Specific gravity	
pH	
Protein	
Glucose	
Ketones	
Bilirubin	
Urobilinogen	
Blood	
Nitrite	
Urine leukocyte esterase	
Microscopic examination of sediment	
**Hormones (female)**	
FSH	Assayed by Lilly-designated laboratory.
**Urine Chemistry**	Assayed by Lilly-designated laboratory.
Albumin	
Creatinine	
Potassium	
Sodium	
**Calculations**	
eGFR (CKD-EPI) calculated using creatinine	Test will be performed locally for screening purposes and by Lilly-designated laboratory for statistical data evaluation. For all other visits, results will be generated by Lilly-designated laboratory.
eGFR (CKD-EPI) calculated using cystatin-c	Generated by Lilly-designated laboratory. Results will not be provided to the investigative sites.
eGFR (CKD-EPI) calculated using creatinine and cystatin-c	Generated by Lilly-designated laboratory. Results will not be provided to the investigative sites.
UACR	Generated by Lilly-designated laboratory.
**Pharmacokinetics Sample -** LY3540378 concentration	Assayed by Lilly-designated laboratory. Results will not be provided to the investigative sites.
**Additional Testing**	
NT-proBNP	Test will be performed locally for screening purposes and by Lilly-designated laboratory for statistical data evaluation.

Final

Approved on 09 Apr 2024 GMT
Downloaded by Flora Sam on 09 Apr 2024

Clinical Laboratory Tests	Comments
	For all other visits, results will be generated by Lilly-designated laboratory. Results will not be provided to the investigative sites.
BNP	Test will be performed locally for screening purposes and by Lilly-designated laboratory for statistical data evaluation.<br>For all other visits, results will be generated by Lilly-designated laboratory. Results will not be provided to the investigative sites.
Cystatin-c	Assayed by Lilly-designated laboratory.
hs-cTnT	Assayed by Lilly-designated laboratory.
hsCRP	Assayed by Lilly-designated laboratory.
HbA1c	Assayed by Lilly-designated laboratory.
HLA – Class II	Assayed by Lilly-designated laboratory<br>Results will not be provided to the investigative sites.
DHEA	Assayed by Lilly-designated laboratory.<br>Results will not be provided to the investigative sites.
Androstenedione	Assayed by Lilly-designated laboratory.<br>Results will not be provided to the investigative sites.
**Genetics Sample**	Assayed by Lilly-designated laboratory.<br>Results will not be provided to the investigative sites.
**Exploratory Biomarker Storage Samples**	Assayed by Lilly-designated laboratory.<br>Results will not be provided to the investigative sites.
Serum	
Plasma (EDTA)	
Urine	
**Immunogenicity Samples**	Assayed by Lilly-designated laboratory.<br>Results will not be provided to the investigative sites.
Anti-LY3540378 antibodies	

Abbreviations: ALP = alkaline phosphatase; ALT = alanine aminotransferase; AST = aspartate aminotransferase; BNP = Brain natriuretic peptide; BUN = blood urea nitrogen; CK = creatine kinase; CKD-EPI = Chronic Kidney Disease Epidemiology Collaboration; DHEA = Dehydroepiandrosterone; EDTA = Ethylenediaminetetraacetic acid; eGFR = estimated glomerular filtration rate; GGT = Gamma-glutamyl transferase; FSH = Follicle-stimulating hormone; HbA1c = Hemoglobin A1c; HDL-C = High-density lipoprotein cholesterol; HLA = Human leukocyte antigen; hsCRP = C-reactive protein, high- sensitivity; hs-cTnT = Troponin. high sensitivity; LDL-C = Low-density lipoprotein cholesterol; NT-proBNP = N-terminal pro BNP; RBC = red blood cells; UACR = Urinary albumin/creatinine ratio; VLDL-C = Very-low-density lipoprotein cholesterol; WBC = white blood cells.

Final

Approved on 09 Apr 2024 GMT
Downloaded by Flora Sam on 09 Apr 2024

10.2.1. Laboratory Samples Obtained at the Time of a Systemic Hypersensitivity Event

Purpose of collecting samples after a systemic hypersensitivity event

The samples listed in this appendix are not collected for acute study participant management. The sponsor will use the laboratory tests results from these samples to characterize hypersensitivity events across the clinical development program.

When to collect samples after a systemic hypersensitivity event occurs

Collect the samples listed below if a systemic hypersensitivity event is suspected. The timing should be as designated in the table, assuming the participant has been stabilized.

Obtain follow-up predose samples at the next regularly scheduled laboratory sample collection, ideally prior to the next dose after the event, to assess post-event return to baseline values.

Timing	Sample Type	Laboratory Test[a]
Collect from 30 min to 4 hr after the start of the event.<br>● Note: The optimal collection time is 1 to 2 hr after the start of event.	Serum	total tryptase
	Serum	complements (C3, C3a, and C5a)
	Serum	cytokine panel (IL-6, IL-1β, IL-10 or any cytokine panel that includes these 3 cytokines)
Collect only if not already collected on the same day as the event.<br>● Note: If collecting, collected up to 12 hr after the start of the event.	Serum	LY3540378 ADA
	Plasma	LY3540378 concentration

Abbreviations: ADA = anti-drug antibodies; IL = interleukin.

[a] All samples for hypersensitivity testing will be assayed by Lilly-designated laboratory. Results will not be provided to the study site. If samples are not collected or are collected outside the specified time period, this will not be considered a protocol deviation.

What information to record

Record the date and time when the samples are collected.

Allowed additional testing for participant management

The investigator may perform additional tests locally, if clinically indicated, for acute study participant management.

Final

Approved on 09 Apr 2024 GMT
Downloaded by Flora Sam on 09 Apr 2024

10.3. Appendix 3: Adverse Events and Serious Adverse Events: Definitions and Procedures for Recording, Evaluating, Follow-up, and Reporting

10.3.1. Definition of AE

AE Definition
• An AE is any untoward medical occurrence in a participant administered a pharmaceutical product and which does not necessarily have a causal relationship with the study intervention. An AE can therefore be any unfavorable and unintended sign (including an abnormal laboratory finding), symptom, or disease (new or exacerbated) temporally associated with the use of a medicinal (investigational) product, whether or not related to the medicinal (investigational) product.

Events Meeting the AE Definition
• Any abnormal laboratory test results (hematology, clinical chemistry, or urinalysis) or other safety assessments (for example, ECG, radiological scans, vital signs measurements), including those that worsen from baseline, considered clinically significant in the medical and scientific judgment of the investigator (that is, not related to progression of underlying disease).<br>• Exacerbation of a chronic or intermittent preexisting condition including either an increase in frequency and/or intensity of the condition.<br>• New condition detected or diagnosed after study intervention administration even though it may have been present before the start of the study.<br>• Signs, symptoms, or the clinical sequelae of a suspected drug-drug interaction.<br>• Medication error, misuse, or abuse of IMP, including signs, symptoms, or clinical sequelae.<br>• Lack of efficacy or failure of expected pharmacological action per se will not be reported as an AE or SAE. Such instances will be captured in the efficacy assessments. However, the signs, symptoms, and/or clinical sequelae resulting from lack of efficacy will be reported as AE or SAE if they fulfill the definition of an AE or SAE.

Events NOT Meeting the AE Definition
• Any clinically significant abnormal laboratory findings or other abnormal safety assessments that are associated with the underlying disease, unless judged by the investigator to be more severe than expected for the participant's condition.<br>• The disease/disorder being studied or expected progression, signs, or symptoms of the disease/disorder being studied, unless more severe than expected for the participant's condition.<br>• Medical or surgical procedure (for example, endoscopy, appendectomy): the condition that leads to the procedure is the AE.

Final

Approved on 09 Apr 2024 GMT
Downloaded by Flora Sam on 09 Apr 2024

- Situations in which an untoward medical occurrence did not occur (social and/or convenience admission to a hospital).
- Anticipated day-to-day fluctuations of preexisting disease(s) or condition(s) present or detected at the start of the study that do not worsen.

10.3.2. Definition of SAE

**An SAE is defined as any untoward medical occurrence that, at any dose, meets 1 or more of the criteria listed:**
**Results in death**
**Is life-threatening**<br>The term *life-threatening* in the definition of *serious* refers to an event in which the participant was at risk of death at the time of the event. It does not refer to an event, which hypothetically might have caused death, if it were more severe.
**Requires inpatient hospitalization or prolongation of existing hospitalization**<br>• In general, hospitalization signifies that the participant has been admitted to hospital or emergency ward (usually involving at least an overnight stay) for observation and/or treatment that would not have been appropriate in the physician's office or outpatient setting. Complications that occur during hospitalization are AEs. If a complication prolongs hospitalization or fulfills any other serious criteria, the event is serious. When in doubt as to whether hospitalization occurred or was necessary, the AE should be considered serious.<br>• Hospitalization for elective treatment of a preexisting condition that did not worsen from baseline is not considered an AE.
**Results in persistent disability or incapacity**<br>• The term disability means a substantial disruption of a person's ability to conduct normal life functions.<br>• This definition is not intended to include experiences of relatively minor medical significance such as uncomplicated headache, nausea, vomiting, diarrhea, influenza, and accidental trauma (for example, sprained ankle) which may interfere with or prevent everyday life functions but do not constitute a substantial disruption.
**Is a congenital anomaly or birth defect**<br>• Abnormal pregnancy outcomes (for example, spontaneous abortion, fetal death, stillbirth, congenital anomalies, ectopic pregnancy) are considered SAEs.
**Other situations:**<br>• Medical or scientific judgment should be exercised by the investigator in deciding whether SAE reporting is appropriate in other situations such as important medical events that may not be immediately life-threatening or result in death or hospitalization but may jeopardize the participant or may require medical or surgical intervention to prevent one

Final

Approved on 09 Apr 2024 GMT
Downloaded by Flora Sam on 09 Apr 2024

of the other outcomes listed in the above definition. These events should usually be considered serious.<br>• Examples of such events include invasive or malignant cancers, intensive treatment in an emergency room or at home for allergic bronchospasm, blood dyscrasias or convulsions that do not result in hospitalization, or development of drug dependency or drug abuse.

10.3.3. Definition of Product Complaints

**Product Complaint**
• A PC is any written, electronic, or oral communication that alleges deficiencies related to the identity, quality, durability, reliability, safety, effectiveness, or performance of a study intervention. When the ability to use the study intervention safely is impacted, the following are also PCs:<br>o Deficiencies in labeling information, and<br>o Use errors for device or drug-device combination products due to ergonomic design elements of the product.<br>• PCs related to study interventions used in clinical trials are collected in order to ensure the safety of participants, monitor quality, and to facilitate process and product improvements.<br>• Investigators will instruct participants to contact the site as soon as possible if he or she has a PC or problem with the study intervention so that the situation can be assessed.<br>• An event may meet the definition of both a PC and an AE/SAE. In such cases, it should be reported as both a PC and as an AE/SAE.

Final

10.3.4. Recording and Follow-Up of AE and/or SAE and Product Complaints

**AE, SAE, and PC Recording**
• When an AE/SAE/PC occurs, it is the responsibility of the investigator to review all documentation (for example, hospital progress notes, laboratory reports, and diagnostics reports) related to the event.<br>• The investigator will then record all relevant AE/SAE/PC information in the participant's medical records, in accordance with the investigator's normal clinical practice. AE/SAE information is reported on the appropriate CRF page and PC information is reported on the PC Form.<br>Note: An event may meet the definition of both a PC and an AE/SAE. In such cases, it should be reported as both a PC and as an AE/SAE.<br>• It is **not** acceptable for the investigator to send photocopies of the participant's medical records to sponsor or designee in lieu of completion of the CRF page for AE/SAE and the PC Form for PCs.<br>• There may be instances when copies of medical records for certain cases are requested by Sponsor or designee. In this case, all participant identifiers, with the exception of the

Approved on 09 Apr 2024 GMT
Downloaded by Flora Sam on 09 Apr 2024

participant number, will be redacted on the copies of the medical records before submission to Sponsor or designee.

- The investigator will attempt to establish a diagnosis of the event based on signs, symptoms, and/or other clinical information. Whenever possible, the diagnosis (not the individual signs or symptoms) will be documented as the AE/SAE.

Assessment of Intensity

The investigator will make an assessment of intensity for each AE and SAE reported during the study and assign it to one of the following categories:

- Mild: A type of AE that is usually transient and may require only minimal treatment or therapeutic intervention. The event does not generally interfere with usual activities of daily living.
- Moderate: A type of AE that is usually alleviated with additional specific therapeutic intervention. The event interferes with usual activities of daily living, causing discomfort but poses no significant or permanent risk of harm to the research participant.
- Severe: A type of AE that interrupts usual activities of daily living, or significantly affects clinical status, or may require intensive therapeutic intervention. An AE that is assessed as severe should not be confused with a SAE. Severe is a category utilized for rating the intensity of an event; and both AEs and SAEs can be assessed as severe.

  An event is defined as 'serious' when it meets at least one of the pre-defined outcomes as described in the definition of an SAE, NOT when it is rated as severe.

Final

Assessment of Causality

- The investigator is obligated to assess the relationship between study intervention and each occurrence of each AE/SAE. The investigator will use clinical judgment to determine the relationship.
- A "reasonable possibility" of a relationship conveys that there are facts, evidence, and/or arguments to suggest a causal relationship, rather than a relationship cannot be ruled out.
- Alternative causes, such as underlying disease(s), concomitant therapy, and other risk factors, as well as the temporal relationship of the event to study intervention administration will be considered and investigated.
- The investigator will also consult the IB in their assessment.
- For each AE/SAE, the investigator must document in the medical notes that he/she has reviewed the AE/SAE and has provided an assessment of causality.
- There may be situations in which an SAE has occurred and the investigator has minimal information to include in the initial report to sponsor or designee. However, it is very important that the investigator always make an assessment of causality for every event before the initial transmission of the SAE data to sponsor or designee.
- The investigator may change their opinion of causality in light of follow-up information and send a SAE follow-up report with the updated causality assessment.

Approved on 09 Apr 2024 GMT
Downloaded by Flora Sam on 09 Apr 2024

• The causality assessment is one of the criteria used when determining regulatory reporting requirements.

Follow-Up of AEs and SAEs
• The investigator is obligated to perform or arrange for the conduct of supplemental measurements and/or evaluations as medically indicated or as requested by sponsor or designee to elucidate the nature and/or causality of the AE or SAE as fully as possible. This may include additional laboratory tests or investigations, histopathological examinations, or consultation with other health care professionals.<br>• If a participant dies during participation in the study or during a recognized follow-up period, the investigator will provide sponsor or designee with a copy of any post-mortem findings including histopathology.

10.3.5. Reporting of SAEs

SAE Reporting via an Electronic Data Collection Tool
• The primary mechanism for reporting an SAE will be the electronic data collection tool.<br>• If the electronic system is unavailable, then the site will use the SAE paper form (see next section) to report the event within 24 hours.<br>• The site will enter the SAE data into the electronic system as soon as it becomes available.<br>• After the study is completed at a given site, the electronic data collection tool will be taken off-line to prevent the entry of new data or changes to existing data.<br>• If a site receives a report of a new SAE from a study participant or receives updated data on a previously reported SAE after the electronic data collection tool has been taken off-line, then the site can report this information on a SAE paper form (see next section) or to the sponsor by telephone.<br>• Contacts for SAE reporting can be found in site training documents.

Final

10.3.6. Regulatory Reporting Requirements

SAE Regulatory Reporting
• Prompt notification by the investigator to the sponsor of a SAE is essential so that legal obligations and ethical responsibilities toward the safety of participants and the safety of a study intervention under clinical investigation are met.

Approved on 09 Apr 2024 GMT
Downloaded by Flora Sam on 09 Apr 2024

- The sponsor has a legal responsibility to notify both the local regulatory authority and other regulatory agencies about the safety of a study intervention under clinical investigation. The sponsor will evaluate the reported SAEs, including confirmation of relatedness and assessment of expectedness. The sponsor has processes for safety reports for identification, recording, and expedited reporting of suspected unexpected serious adverse reactions (SUSARs) according to local regulatory requirements. The sponsor will comply with country-specific regulatory requirements relating to safety reporting to the regulatory authority, IRB/IEC, and investigators.
- An investigator who receives an investigator safety report describing a SAE or other specific safety information (for example, summary or listing of SAEs) from the sponsor will review and then file it along with the IB and will notify the IRB/IEC, if appropriate according to local requirements.

Final

Approved on 09 Apr 2024 GMT
Downloaded by Flora Sam on 09 Apr 2024

10.4. Appendix 4: Contraceptive and Barrier Guidance

10.4.1. Definitions

Women of childbearing potential

Adult females are considered WOCBP unless they are WNOCBP.

Women not of childbearing potential

Females are considered WNOCBP if they

- have a congenital anomaly such as Müllerian agenesis
- are infertile due to surgical sterilization, or
- are postmenopausal.

Examples of surgical sterilization include total hysterectomy, bilateral salpingo-oophorectomy, bilateral salpingectomy, or bilateral oophorectomy.

Postmenopausal state

The postmenopausal state is defined as a woman:

- at any age at least 6 weeks post-surgical bilateral oophorectomy with or without hysterectomy, confirmed by operative note; or
- aged at least 40 years and up to 55 years with an intact uterus, not on hormone therapy[a], who has had cessation of menses for at least 12 consecutive months without an alternative medical cause, AND with a follicle-stimulating hormone >40 mIU/mL; or
- 55 years or older not on hormone therapy, who has had at least 12 months of spontaneous amenorrhea, or

[a] Women should not be taking medications during amenorrhea such as oral contraceptives, hormones, gonadotropin-releasing hormone, anti-estrogens, selective estrogen receptor modulators, or chemotherapy that could induce transient amenorrhea.

Final

Approved on 09 Apr 2024 GMT
Downloaded by Flora Sam on 09 Apr 2024

10.4.2. Contraception Guidance

The table below describes contraception guidance for men.

Topic	Guidance
For all men	should refrain from sperm donation for the duration of the study and for 135 days.
Contraception for men with partners of childbearing potential	• either remain abstinent (if this is their preferred and usual lifestyle), or<br>• must use condoms during intercourse for the duration of the study, and<br>• for 135 days after end of study.
Contraception for men in exclusively same sex relationships, as their preferred and usual lifestyle	Are not required to use contraception

Methods of contraception for male participants and their female partners

Methods	Examples
Highly effective contraception (less than 1% failure rate)	female sterilization<br>combination oral contraceptive pill<br>progestin-only contraceptive pill (mini-pill)<br>implanted contraceptives<br>injectable contraceptives<br>contraceptive patch (only women <198 pounds or 90 kg)<br>total abstinence<br>vasectomy (if only sexual partner)<br>fallopian tube implants (if confirmed by hysterosalpingogram)<br>combined contraceptive vaginal ring, or<br>intrauterine devices
Effective contraception	barrier method with use of a spermicide<br>• male condom with spermicide<br>• diaphragm with spermicide or cervical sponge, or<br>• female condom with spermicide<br>Note: The barrier method must include use of a spermicide (that is, condom with spermicide, diaphragm with spermicide, or female condom with spermicide) to be considered effective.
Ineffective forms of contraception whether used alone or in any combination	• spermicide alone<br>• periodic abstinence<br>• fertility awareness (calendar method, temperature method, cervical mucus, or symptothermal)<br>• withdrawal<br>• postcoital douche, or<br>• lactational amenorrhea

Final

Approved on 09 Apr 2024 GMT
Downloaded by Flora Sam on 09 Apr 2024

10.5. Appendix 5: Genetics

Use/Analysis of DNA

- Genetic variation may impact a participant's response to study intervention, susceptibility to, and severity and progression of disease. Variable response to study intervention may be due to genetic determinants that impact drug absorption, distribution, metabolism, and excretion; mechanism of action of the drug; disease etiology; and/or molecular subtype of the disease being treated. Therefore, where local regulations and IRB/IEC allow, an optional blood sample will be collected for DNA analysis from consenting participants.
- DNA samples will be used for research related to HF and related diseases. They may also be used to develop tests or assays including diagnostic tests related to LY3540378 and HFpEF. Genetic research may consist of the analysis of one or more candidate genes or the analysis of genetic markers throughout the genome or analysis of the entire genome (as appropriate).
- The samples may be analyzed as part of a multi-study assessment of genetic factors involved in the response to LY3540378 or study interventions of this class to understand study disease or related conditions.
- The results of genetic analyses may be reported in the CSR or in a separate study summary.
- The sponsor will store the DNA samples in a secure storage space with adequate measures to protect confidentiality.

The samples will be retained while research on LY3540378 continues but no longer than as indicated in Section 10.1.12.

Final

Approved on 09 Apr 2024 GMT
Downloaded by Flora Sam on 09 Apr 2024

10.6. Appendix 6: Liver Safety: Suggested Actions and Follow-up Assessments

10.6.1. Hepatic Evaluation Testing

See Section 8.3.5 for guidance on appropriate test selection.

The Lilly-designated central laboratory should complete the analysis of all selected testing except for testing listed in the investigator-designated local laboratory table. The central laboratory will report results if a validated test or calculation is available.

Local testing may be performed *in addition to central testing* when necessary for immediate participant management.

The local laboratory must be qualified in accordance with applicable local regulations. If testing is not available in certain regions based on local requirements, consult with Lilly-designated medical monitor.

**Tests assayed by Lilly-designated central laboratory**	
**Hepatic Hematology Panel**	**Hepatitis A virus (HAV) testing:**
Hemoglobin	HAV total antibody[a]
Hematocrit	HAV IgM antibody
Erythrocytes (RBCs - red blood cells)	**Hepatis B virus (HBV) testing:**
Leukocytes (WBCs - white blood cells)	Hepatitis B surface antigen (HBsAg)
Differential:	Hepatitis B surface antibody (anti-HBs)
Neutrophils	Hepatitis B core total antibody (anti-HBc)
Lymphocytes	Hepatitis B core IgM antibody
Monocytes	HBV DNA[b]
Basophils	**Hepatis C virus (HCV) testing:**
Eosinophils	HCV total antibody[a]
Platelets	HCV RNA[b]
Cell morphology (RBC and WBC)	**Hepatitis D virus (HDV) testing[c]:**
**Hepatic Clinical Chemistry Panel**	HDV total antibody[a]
Total bilirubin	HDV IgM antibody
Direct bilirubin	HDV RNA[b]
Alkaline phosphatase (ALP)	**Hepatitis E virus (HEV) testing:**
Alanine aminotransferase (ALT)	HEV IgG antibody
Aspartate aminotransferase (AST)	HEV IgM antibody
Gamma-glutamyl transferase (GGT)	HEV RNA[b]
Creatine kinase (CK)	**Anti-nuclear antibody (ANA)**
**Hepatic Coagulation Panel**	**Anti-smooth muscle antibody (ASMA) or anti-actin antibody**
Prothrombin time, INR (PT-INR)	**Immunoglobulin IgA (quantitative)**
**Urine Chemistry**	**Immunoglobulin IgG (quantitative)**
Drug screen	**Immunoglobulin IgM (quantitative)**
**Haptoglobin**	

**Tests assayed by investigator-designated local laboratory**

Approved on 09 Apr 2024 GMT
Downloaded by Flora Sam on 09 Apr 2024

**Acetaminophen**	**Cytomegalovirus (CMV) testing:**
**Acetaminophen protein adducts[d]**	CMV antibody
**Alkaline phosphatase isoenzymes**	CMV DNA[b]
**Ceruloplasmin**	**Herpes simplex virus (HSV) testing:**
**Copper**	HSV (Type 1 and 2) antibody
**Ethyl alcohol (EtOH)**	HSV (Type 1 and 2) DNA[b]
**Phosphatidylethanol (PEth)**	Liver kidney microsomal type 1 (LKM-1) antibody
**Urine Chemistry**	**Microbiology**
Ethyl glucuronide (EtG)	Culture:
**Epstein-Barr virus (EBV) testing:**	Blood
EBV antibody	Urine
EBV DNA[b]	

a If lab does not offer total antibody testing, IgG and IgM are acceptable substitute.

b Reflex/confirmation dependent on regulatory requirements, testing availability, or both.

c If HDV testing is not available, HBV testing may be sufficient. If HBV testing is positive, consult with the Lilly-designated medical monitor.

d Availability of acetaminophen protein adducts testing is limited, so testing may be performed at central labs, if needed.

Final

Approved on 09_Apr 2024 GMT
Downloaded by Flora Sam on 09 Apr 2024

Appendix 7: Provisions for Changes in Study Conduct During Exceptional Circumstances

Implementation of this appendix

The changes to procedures described in this appendix are temporary measures intended to be used only during specific time periods as directed by the sponsor in partnership with the investigator.

Exceptional circumstances

Exceptional circumstances are rare events that may cause disruptions to the conduct of the study. Examples include pandemics or natural disasters. These disruptions may limit the ability of the investigators, participants, or both to attend on-site visits or to conduct planned study procedures.

Implementing changes under exceptional circumstances

In an exceptional circumstance, after receiving the sponsor's written approval, sites may implement changes if permitted by local regulations.

After approval by local Ethical Review Boards, regulatory bodies, and any other relevant local authorities, implementation of these exceptional circumstance changes will not typically require additional notification to these groups, unless they have specific requirements in which notification is required (for example, upon implementation and suspension of changes). All approvals and notifications must be retained in the study records.

If the sponsor grants written approval for changes in study conduct, the sponsor will also provide additional written guidance, if needed.

Final

Considerations for making a change

The prevailing consideration for making a change is ensuring the safety of study participants. Additional important considerations for making a change are compliance with GCP, enabling participants to continue safely in the study and maintaining the integrity of the study.

Informed consent

Additional consent from the participant will be obtained, if required, for:

- participation in remote visits, as defined in Section "Remote Visits,"
- alternate delivery of study intervention and ancillary supplies, and
- provision of their personal or medical information required prior to implementation of these activities.

Changes in study conduct during exceptional circumstances

Changes in study conduct not described in this appendix, or not consistent with applicable local regulations, are not allowed.

The following changes in study conduct will not be considered protocol deviations.

Approved on 09 Apr 2024 GMT
Downloaded by Flora Sam on 09 Apr 2024

Remote visits

Types of remote visits

Telemedicine: Telephone or technology-assisted virtual visits, or both, are acceptable to complete appropriate assessments. Assessments to be completed in this manner include, but are not limited to, diary review, AE review, concomitant medications, etc.
Mobile healthcare: Healthcare visits may be performed by a mobile healthcare provider at locations other than the study site when participants cannot travel to the site due to an exceptional circumstance if written approval is provided by the sponsor. Procedures performed at such visits include, but are not limited to, concomitant medications, collection of blood samples, physical assessments, administration of PROs if validated for these types of visits, administration of study intervention, and collection of health information.
Other alternative locations: A local laboratory may be used for laboratory draws.

Data capture

In source documents and the CRF, the study site should capture the visit method, with a specific explanation for any data missing because of missed in-person site visits.

Safety reporting

Regardless of the type of remote visits implemented, the protocol requirements regarding the reporting of AEs, SAEs, and PCs remain unchanged.

Return to on-site visits

Every effort should be made to enable participants to return to on-site visits as soon as reasonably possible, while ensuring the safety of both the participants and the site staff.

Local laboratory testing option

Local laboratory testing may be conducted in lieu of central laboratory testing. However, central laboratory testing must be retained for NTpro-BNP. The local laboratory must be qualified in accordance with applicable local regulations.

Study intervention and ancillary supplies (including participant diaries)

When a participant is unable to go to the site to receive study supplies during normal on-site visits, the site should work with the sponsor to determine appropriate actions. These actions may include:

- asking the participant to go to the site and receive study supplies from site staff without completion of a full study visit,
- asking the participant's designee to go to the site and receive study supplies on a participant's behalf,
- arranging delivery of study supplies, and
- working with the sponsor to determine how study intervention that is typically administered on site will be administered to the participant; for example, during a mobile healthcare visit or at an alternate location such as an infusion center.

Final

Approved on 09 Apr 2024 GMT
Downloaded by Flora Sam on 09 Apr 2024

These requirements must be met before action is taken:

- Alternate delivery of study intervention should be performed in a manner that does not compromise treatment blinding and ensures product integrity. The existing protocol requirements for product accountability remain unchanged, including verification of participant's receipt of study supplies.
- When delivering supplies to a location other than the study site (for example, participant's home), the investigator, sponsor, or both should ensure oversight of the shipping process to ensure accountability and product quality (that is, storage conditions maintained and intact packaging upon receipt).
- Instructions may be provided to the participant or designee on the final disposition of any unused or completed study supplies.

If study intervention will be administered to the participant during a mobile healthcare visit or at an alternate location, this additional requirement must be met:

- Only authorized study personnel may supply, prepare, or administer study intervention.

Adjustments to visit windows

Whenever possible and safe to do so, as determined by the investigator's discretion, participants should complete the usual SoA. To maximize the possibility that these visits can be conducted as on-site visits, the windows for visits may be adjusted, upon further guidance from the sponsor. This minimizes missing data and preserves the intended conduct of the study.

Final

This table describes the allowed adjustments to visit windows.

Visit Number	Tolerance
Visits 3 through 24	±3 days from intended date
Visits 801 and 802	within 3 days before the intended date, or up to 7 days after the intended date

For participants whose visits have extended windows, additional study intervention may need to be provided to avoid interruption and maintain overall integrity of the study.

Documentation

Changes to study conduct will be documented

Sites will identify and document the details of how participants, visit types, and conducted activities were affected by exceptional circumstances. Dispensing or shipment records of study intervention and relevant communications, including delegation, should be filed with site study records.

Source documents at alternate locations

Approved on 09 Apr 2024 GMT
Downloaded by Flora Sam on 09 Apr 2024

Source documents generated at a location other than the study site should be part of the investigator's source documentation and should be transferred to the site in a secure and timely manner.

The below figure provides an overview of screening and randomization.

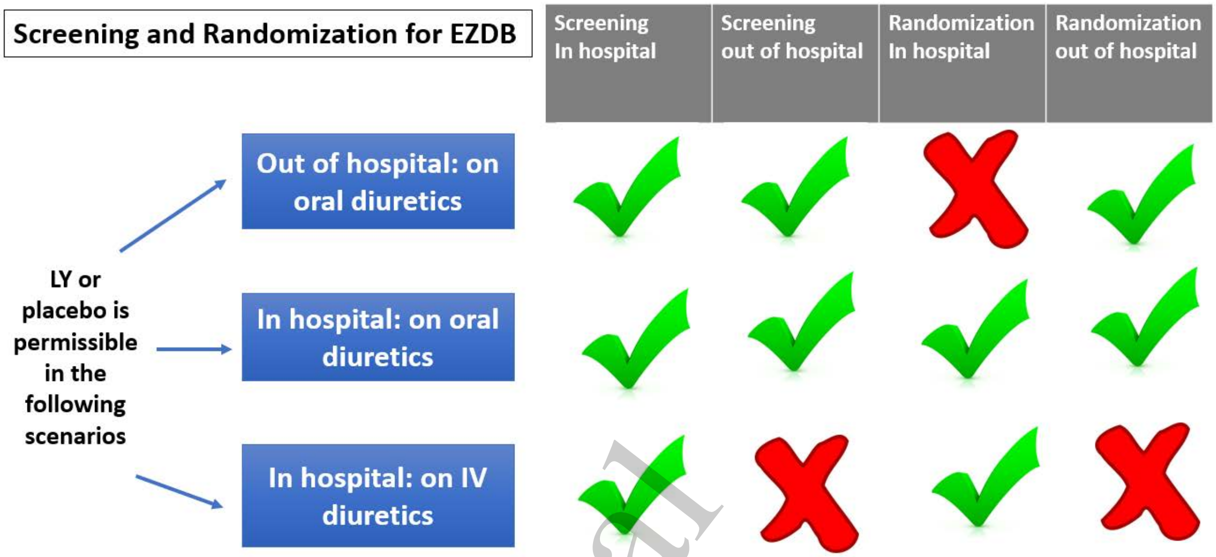

Final

Approved on 09 Apr 2024 GMT
Downloaded by Flora Sam on 09 Apr 2024

10.7. Appendix 8: Abbreviations and Definitions

Term	Definition
**Abuse**	Use of a study intervention for recreational purposes or to maintain an addiction or dependence
**ADA**	anti-drug antibody
**AE**	adverse event
**ALP**	alkaline phosphatase
**ALT**	alanine aminotransferase
**AST**	aspartate aminotransferase
**authorized IMP**	*Applicable to the EU only*: a medicinal product authorized in accordance with Regulation (EC) No 726/2004 or in any Member State concerned in accordance with Directive 2001/83/EC, irrespective of changes to the labelling of the medicinal product, which is used as an investigational medicinal product
**authorized AxMP**	*Applicable to the EU only*: a medicinal product authorized in accordance with Regulation (EC) No 726/2004, or in any Member State concerned in accordance with Directive 2001/83/EC, irrespective of changes to the labelling of the medicinal product, which is used as an auxiliary medicinal product
**AxMP**	auxiliary medicinal product. See also NIMP.<br>A medicinal product used for the needs of a clinical trial as described in the protocol, but not as an investigational medicinal product. Examples include rescue medication, challenge agents, agents to assess endpoints in the clinical trial, or background treatment. AxMP does not include investigational medicinal product (IMP) or concomitant medications. Concomitant medications are medications unrelated to the clinical trial and not relevant for the design of the clinical trial
**blinding**	A single-blind study is one in which the investigator and/or the investigator's staff are aware of the treatment but the participant is not, or vice versa, or when the sponsor is aware of the treatment but the investigator and/the investigator's staff and the participant are not.<br>A double-blind study is one in which neither the participant nor any of the investigator or sponsor staff who are involved in the treatment or clinical evaluation of the participants are aware of the treatment received.
**BNP**	brain natriuretic peptide
**BP**	blood pressure
**CKD-EPI**	Chronic Kidney Disease Epidemiology Collaboration
**Companion diagnostic**	An in vitro diagnostic device (assay or test) that provides information that is essential for the safe and effective use of a corresponding therapeutic product
**complaint**	A complaint is any written, electronic, or oral communication that alleges deficiencies related to the identity, quality, purity, durability, reliability, safety or effectiveness, or performance of a drug or drug delivery system.

Final

Approved on 09 Apr 2024 GMT
Downloaded by Flora Sam on 09 Apr 2024

**compliance**	Adherence to all study-related, good clinical practice (GCP), and applicable regulatory requirements.
**COPD**	chronic obstructive pulmonary disease
**CSR**	clinical study report
**CRF**	case report form; a printed, optical, or electronic document designed to record all of the protocol-required information to be reported to the sponsor for each trial participant.
**CT**	computed tomography
**CV**	cardiovascular
**DHEA**	dehydroepiandrosterone
**EAS**	efficacy analysis set
**ECG**	electrocardiogram
**EDC**	electronic data capture
**ECHO**	echocardiogram
**eCOA**	electronic clinical outcome assessment
**eGFR**	estimated glomerular filtration rate
**enroll**	The act of assigning a participant to a treatment. Participants who are enrolled in the study are those who have been assigned to a treatment.
**Enter**	Participants entered into a study are those who sign the informed consent form directly or through their legally acceptable representatives.
**GCP**	Good Clinical Practice
**GDPR**	EU-General Data Protection Regulation
**HF**	heart failure
**HFpEF**	heart failure with preserved ejection fraction
**HLA class II**	human leukocyte antigen class II
**IB**	Investigator's Brochure
**AC**	Assessment Committee
**ICF**	informed consent form
**ICH**	International Council for Harmonisation
**IMP**	Investigational Medicinal Product (see also "investigational product")<br>A medicinal product which is being tested or used as a reference, including as a placebo, in a clinical trial
**Index event**	A recent hospitalization for HF requiring at least 2 doses of intravenous diuretics or an out- of- hospital encounter (for example, Emergency Room, clinic visit, infusion clinic, etc.) for HF requiring at least 2 doses of intravenous diuretics.

Final

Approved on 09 Apr 2024 GMT
Downloaded by Flora Sam on 09 Apr 2024

**informed consent**	A process by which a participant voluntarily confirms their willingness to participate in a particular study, after having been informed of all aspects of the study that are relevant to the participant's decision to participate. Informed consent is documented by means of a written, signed, and dated informed consent form.
**investigational product**	A pharmaceutical form of an active ingredient or placebo being tested or used as a reference in a clinical trial, including products already on the market when used or assembled (formulated or packaged) in a way different from the authorized form, or marketed products used for an unauthorized indication, or marketed products used to gain further information about the authorized form.
**ISR**	Injection site reaction
**IV**	Intravenous
**IWRS**	interactive web-response system
**KCCQ**	Kansas City Cardiomyopathy Questionnaire
**LA**	left atrium
**LAEDVI**	left atrial end-diastolic volume index
**LAESVI**	left atrial end-systolic volume index
**LARS**	left atrial reservoir strain
**LV**	left ventricle
**LVAD**	left ventricular assist device
**LVEDV**	left ventricular end-diastolic volume
**LVESV**	left ventricular end-systolic volume
**LVEF**	left ventricular ejection fraction
**LVGLS**	left ventricular global longitudinal strain
**MAD**	multiple-ascending dose
**MedDRA**	Medical Dictionary for Regulatory Activities

Final

Approved on 09 Apr 2024 GMT
Downloaded by Flora Sam on 09 Apr 2024

**Medication error**	Errors in the prescribing, dispensing, or administration of a study intervention, regardless of whether or not the medication is administered to the participant or the error leads to an AE. Medication error generally involve a failure to uphold one or more of the five "rights" of medication use: the right participant, the right drug, the right dose, right route, at the right time.<br>In addition to the core five rights, the following may also represent medication errors:<br>• dose omission associated with an AE or a product complaint<br>• dispensing or use of expired medication<br>• use of medication past the recommended in-use date<br>• dispensing or use of an improperly stored medication<br>• use of an adulterated dosage form or administration technique inconsistent with the medication's labeling (for example, Summary of Product Characteristics, IB, local label, protocol), or<br>• shared use of cartridges, prefilled pens, or both.
**misuse**	Use of a study intervention for self-treatment that either is inconsistent with the prescribed dosing regimen, indication, or both, or is obtained without a prescription
**MMRM**	mixed model for repeated measures
**MRI**	magnetic resonance imaging
**NIMP**	non-investigational medicinal product See AxMP<br>A medicinal product used for the needs of a clinical trial as described in the protocol, but not as an investigational medicinal product. Examples include rescue medication, challenge agents, agents to assess endpoints in the clinical trial, or background treatment.
**NRS**	Numeric Rating Scale
**NT-proBNP**	N-terminal pro-B-type natriuretic peptide
**NYHA**	New York Heart Association
**participant**	Equivalent to CDISC term "subject": an individual who participates in a clinical trial, either as recipient of an investigational medicinal product or as a control
**PC**	product complaint
**PCWP**	pulmonary capillary wedge pressure
**PGIC**	Patient Global Impression of Change
**PGIS**	Patient Global Impression of Status
**PK/PD**	pharmacokinetics/pharmacodynamics
**PPS**	per-protocol set: The set of data generated by the subset of participant who sufficiently complied with the protocol to ensure that these data would be likely to exhibit the effects of treatment, according to the underlying scientific model.
**PRO/ePRO**	patient-reported outcomes/electronic patient-reported outcomes

Final

Approved on 09 Apr 2024 GMT
Downloaded by Flora Sam on 09 Apr 2024

**PT-INR**	prothrombin time – international normalized ratio
**QTc**	corrected QT interval
**QW**	once a week
**RXFP1**	relaxin family peptide receptor 1
**RXFP2**	relaxin family peptide receptor 2
**SAD**	single-ascending dose
**SAE**	serious adverse event
**SAP**	statistical analysis plan
**SBP**	systolic blood pressure
**SC**	subcutaneous
**screen**	The act of determining if an individual meets minimum requirements to become part of a pool of potential candidates for participation in a clinical study.
**SGLT-2i**	sodium-glucose cotransporter-2 inhibitor
**SOA**	schedule of activities
**SS**	safety analysis set
**SUSAR**	Suspected unexpected serious adverse reactions<br>Refers to an adverse event that occurs in a clinical trial participant, which is assessed by the sponsor and or study investigator as being unexpected, serious and as having a reasonable possibility of a causal relationship with the study intervention.
**TBL**	total bilirubin
**TD**	temporary discontinuation
**TE-ADA**	Treatment emergent antidrug antibodies
**TEAE**	Treatment-emergent adverse event: An untoward medical occurrence that emerges during a defined treatment period, having been absent pretreatment, or worsens relative to the pretreatment state, and does not necessarily have to have a causal relationship with this treatment.
**ULN**	upper limit of normal
**WHF**	worsening heart failure
**WOCBP**	women of childbearing potential

Final

Approved on 09_Apr 2024 GMT
Downloaded by Flora Sam on 09 Apr 2024

10.9. Appendix 9: Protocol Amendment History

The Protocol Amendment Summary of Changes Table for the current amendment is located directly before the Table of Contents (TOC).

Amendment [a]: (01-Jun-2023)

This amendment is considered to be substantial.

The amendment is considered to be substantial because it is likely to have a significant impact on the safety or the rights of the study participants.

Overall Rationale for the Amendment:

The overall rationale of this amendment is to modify the inclusion and exclusion criteria and schedule of activities.

Section # and Name	Description of Change	Brief Rationale
Title page	Included both EudraCT number and EU CT number	To bring the protocol under EU CT Regulation
1.1 Synopsis	Updated the section to align with the changes in the main body of the protocol and as per latest protocol template	For consistency
1.3. Schedule of Activities (SoA)<br>Visits 1 through 16	Updated the screening period to Visit 1 only. Initially the screening period was extended to Visit 3.	Correction
	In 'Weeks from randomization' row, replaced "-1" with "≤2" for Visit 1	For clarification
	Removed the Visit interval tolerance (days) for Visit 1	For participant's convenience
	Changed Visits 7, 8, and 9 to telehealth visits	For participant's convenience
	Deleted the measurement of Weight, Vital signs, Physical examination, Register visit with IWRS, Dispense study intervention via IWRS, Administer study intervention on site, Train participants and/or caregiver in study intervention administration, at Visits 7, 8, and 9.	For clarification
	Deleted the measurement of Patient-Reported Outcomes – Most bothersome symptom, Dyspnea NRS, Edema NRS, Fatigue NRS, PGIS-HF Overall Health, PGIC-HF Overall Health, PGIS-HF Symptom Severity, and PGIC-HF Symptom Severity at Visits 7 and 9.	For clarification
	To "Vital signs" row, added "Includes BP (supine and standing), pulse rate, respiratory rate, and temperature." in comments section	Correction

Final

Approved on 09 Apr 2024 GMT
Downloaded by Flora Sam on 09 Apr 2024

Section # and Name	Description of Change	Brief Rationale
	To “Genetics sample” row, added “is optional and”	Correction
	Removed “Diary dispensed” at Visit 12 and added to Visit 5	For clarification
	Removed “Diary review” at Visits 14 and 16 and added at Visit 10	For clarification
	Added “V7, V8, V9” in comments section	For clarification
1.3. Schedule of Activities (SoA)<br>• Visits 17 through 24, ED, and Follow-Up	Added “Concomitant medications”, “AEs”, “Vital signs”, “Physical examination”, “12-lead ECG” at UV visit	Correction
	To “Vital signs” row, added “Includes BP (supine and standing), pulse rate, respiratory rate, and temperature.” in comments section	Correction
	To “12-lead ECG” row, added “Collect ECG at UV if medically indicated at PI discretion”	For clarification
3. Objectives, Endpoints, and Estimands	Added “Log-transformed” to NT-proBNP endpoint	For clarification. Using log(NTproBNP) instead of NTproBNP alone will reduce the variability
	Removed “laboratory parameters”, “ECG”, and “vital signs” as endpoints for safety objective	Correction
	Added “Blood pressure and pulse rate” to tertiary endpoint	For clarification
	Added tertiary endpoint “Change from baseline to the average of Week 24 and Week 26 in log-transformed NT-proBNP”	Using log(NTproBNP) instead of NTproBNP alone will reduce the variability which may better detect the treatment effect
4.1. Overall Design	Changed the screening period from “13” to “14” days	For participant’s convenience
5.1. Inclusion Criteria	Modified the inclusion criterion 2	For clarification
	Modified the inclusion criterion 3	For clarification
	Modified the inclusion criterion 4	To expand the access to eligible participants
	Modified the inclusion criterion 6	To expand the access to eligible participants
	In inclusion criterion 7, replaced “900” with “600” and “300” with “200” pg/mL	To expand the access to eligible participants
	In inclusion criterion 8, changed the lower cut-off of eGFR from “>30” to “>20” and removed the upper cut-off of eGFR “and <75 ml/min/1.73 m^2”	To expand the access to eligible participants
	Modified the inclusion criterion 9	For clarification
5.2. Exclusion Criteria	Deleted exclusion criterion 17	For clarification

Final

Approved on 09 Apr 2024 GMT
Downloaded by Flora Sam on 09 Apr 2024

Section # and Name	Description of Change	Brief Rationale
	In exclusion criterion 14, added “and PVA (pulmonary vein isolation ablation)”	For clarification
	In exclusion criterion 19, added “uncorrected”	Correction
	Modified the exclusion criterion 20	For clarification
	Modified the exclusion criterion 21	For clarification
	Modified the exclusion criterion 22	To expand the access to eligible participants
	Added “HF” to exclusion criterion 23	For clarification
	Modified the exclusion criterion 24	For clarification
	Modified the exclusion criterion 26	For clarification
	Modified the exclusion criterion 27	For clarification
	Modified the exclusion criterion 29	For clarification
	In exclusion criterion 32, removed “platelet dysfunction, hemophilia, von Willebrand disease” and added “bleeding or”	For clarification
	Removed “seated” in exclusion criterion 33	For clarification
	Modified the exclusion criterion 38	For clarification
	In exclusion criterion 40, added “Although not inotropes, IV carperitide and IV Tolvaptan are permitted.”	For clarification
5.3. Lifestyle Considerations	Replaced “8 weeks” with “135 days”	For consistency
6. Study Interventions and Concomitant Therapy	Updated the language	Updated as per latest protocol template
6.5. Dose Modification	Removed “orthostatic hypotension (as defined in Section 8.3.7.3)” and updated the language	For participant’s convenience
	Added “And up titrate from 50 to 100 mg or lower back from 100 to 50 mg as needed” to third column of “Third occurrence” row	For clarification
7.1.4. Temporary Discontinuation	Removed “or significant orthostatic hypotension drop” and “orthostatic hypotension,”	For clarification
8.1.2. Other Efficacy Assessments	Added “Log-transformed” to NT-proBNP	For clarification
8.2.1. Physical Examinations	Added “, as specified in SoA”	For clarification
8.3.1. Timing and Mechanism for Collecting Events	Replaced “45” with “135” days in “Pregnancy in female participants and female partners of male participants” row	To update as per contraceptive guidance
	Changed the mechanism for reporting from “Pregnancy paper form” to	To update the mechanism of reporting

Final

Approved on 09 Apr 2024 GMT
Downloaded by Flora Sam on 09 Apr 2024

Section # and Name	Description of Change	Brief Rationale
	"Pregnancy CRF" in "Pregnancy in female participants and female partners of male participants" row	
8.3.6. Major Adverse Cardiovascular Events (MACE)	Added "urgent HF visits" to the list of nonfatal cardiovascular AEs to be adjudicated	Correction
8.3.7.2. Breast screening	Added "Abnormal finding during breast screening per local guidelines will be recorded as AE."	For clarification
8.4. Pharmacokinetics	Added "safety"	For clarification
8.6. Genetics	Replaced "will" with "may" to modify the language to allow for collection of genetic sampling to be optional	Correction
9.1. Statistical Hypotheses	Modified the language in the first paragraph	For clarification
9.3.1. General Considerations	Removed paragraph 3	For clarification
	Removed continuous data and categorical data paragraphs	Correction
	Removed "atrial fibrillation/atrial flutter (defined as evidence of atrial fibrillation or atrial flutter on the screening ECG) (yes/no)"	For clarification
	Modified the language regarding the additional covariates and regression models	For clarification
9.3.4. Tertiary Endpoints Analysis	Removed the text	For clarification
9.3.5.1. Central Laboratory Measures, Vital Signs, and Electrocardiograms	Removed paragraphs 2 and 3	Correction
9.4. Interim Analysis	Modified the language to include up to 4 interim analyses	For re-evaluation of sample size OR to trigger activities for future development
	Added language to clarify early access to PK and PD data	For clarification
10.1.4. Data Protection	Updated the language	Updated as per latest protocol template
10.1.5. Committees Structure	Removed text related to "Internal assessment committee" and added "(hospitalization and urgent visit)" in last paragraph	For clarification
10.3.6. Regulatory Reporting Requirements	Updated the language	Updated as per latest protocol template
10.5. Appendix 5: Genetics	Added "optional"	Correction
Throughout the protocol	Minor formatting and editorial changes	Minor, therefore, not detailed

Final

Approved on 09 Apr 2024 GMT
Downloaded by Flora Sam on 09 Apr 2024

11. References

Anker SD, Butler J, Filippatos G, et al. Empagliflozin in heart failure with a preserved ejection fraction. *N Engl J Med*. 2021;385(16):1451-1461. https://doi.org/10.1056/NEJMoa2107038

Bathgate RAD, Halls ML, van der Westhuizen ET, et al. Relaxin family peptides and their receptors. *Physiol Rev*. 2013;93(1):405-480. https://doi.org/10.1152/physrev.00001.2012

Benjamin EJ, Muntner P, Alonso A, et al. Heart Disease and Stroke Statistics -2019 Update: A Report from the American Heart Association. *Circulation*. 2019;139(10):e56-e528. https://doi.org/10.1161/CIR.0000000000000659

Conrad KP. Maternal vasodilation in pregnancy: the emerging role of relaxin. *Am J Physiol Regul Integr Comp Physiol*. 2011;301(2):R267-R275. https://doi.org/10.1152/ajpregu.00156.2011

Dschietzig T, Teichman S, Unemori E, et al. Intravenous recombinant human relaxin in compensated heart failure: a safety, tolerability, and pharmacodynamic trial. *J Card Fail*. 2009;15(3):182-190. https://doi.org/10.1016/j.cardfail.2009.01.008

[FDA] Food and Drug Administration. FDA-Approved Drugs. Accessed 9 April 2022. https://www.accessdata.fda.gov/scripts/cder/daf/

Gentile F, Ghionzoli N, Borrelli C, et al. Epidemiological and clinical boundaries of heart failure with preserved ejection fraction. *Eur J Prev Cardiol*. 2021;zwab077. https://doi.org/10.1093/eurjpc/zwab077

Green CP, Porter CB, Bresnahan DR, Spertus JA. Development and evaluation of the Kansas City Cardiomyopathy Questionnaire: a new health status measure for heart failure. J Am Coll Cardiol. 2000;35(5):1245-1255. https://doi.org/10.1016/S0735-1097(00)00531-3

Heidenreich PA, Albert NM, Allen LA, et al. Forecasting the impact of heart failure in the United States: a policy statement from the American heart association. *Circ Heart Fail.* 2013;6(3):606-619. https://doi.org/10.1161/HHF.0b013e318291329a

Heidenreich PA, Bozkurt B, Aguilar D, et al. 2022 AHA/ACC/HFSA Guideline for the Management of Heart Failure: A Report of the American College of Cardiology/American Heart Association Joint Committee on Clinical Practice Guidelines. *J Am Coll Cardiol.* 2022a;79(17):e263-e421. https://doi.org/10.1016/j.jacc.2021.12.012

Heidenreich PA, Fonarow GC, Opsha Yekaterina O, et al. Economic Issues in Heart Failure in the United States. *J Card Fail.* 2022b;28(3):453-466. https://doi.org/10.1016/j.cardfail.2021.12.017

Inker LA, Eneanya ND, Coresh J, et al. New Creatinine- and Cystatin C-Based Equations to Estimate GFR without Race. *N Engl J Med.* 2021;385(19):1737-1749. https://doi.org/10.1056/NEJMoa2102953

Joseph SM, Novak E, Arnold SV, et al. Comparable performance of the Kansas City Cardiomyopathy Questionnaire in patients with heart failure with preserved and reduced ejection fraction. Circ Heart Fail. 2013;6(6):1139-1146. https://doi.org/10.1161/CIRCHEARTFAILURE.113.000359

Final

Approved on 09 Apr 2024 GMT
Downloaded by Flora Sam on 09 Apr 2024

Lekavich CL, Abraham D, Fudim M, et al. Early identification of patients at risk for incident heart failure with preserved ejection fraction: novel approach to echocardiographic trends. *J Card Fail.* 2021;27(9):942-948. https://doi.org/10.1016/j.cardfail.2021.03.013

[Lilly] Eli Lilly and Company. October 2020. Investigator's Brochure for LY3540378.

Morris DA, Belyavskiy E, Aravind-Kumar R, et al. Potential Usefulness and Clinical Relevance of Adding Left Atrial Strain to Left Atrial Volume Index in the Detection of Left Ventricular Diastolic Dysfunction. *JACC Cardiovasc Imaging*. 2018;11(10):1405-1415. https://doi.org/10.1016/j.jcmg.2017.07.029

Owan TE, Hodge DO, Herges RM, et al. Trends in prevalence and outcome of heart failure with preserved ejection fraction. *N Engl J Med*. 2006;355(3):251-259. https://doi:10.1056/NEJMoa052256

Patel RB, Lam CSP, Svedlund S, et al. Disproportionate left atrial myopathy in heart failure with preserved ejection fraction among participants of the PROMIS-HFpEF study. *Sci Rep.* 2021;11(1):4885. https://doi.org/10.1038/s41598-021-84133-9

Ponikowski P, Mitrovic V, Ruda M, et al. A randomized, double-blind, placebo-controlled, multicentre study to assess haemodynamic effects of serelaxin in patients with acute heart failure. *Eur Heart J*. 2014;35(7):431-441. https://doi.org/10.1093/eurheartj/eht459

Santos AB, Roca GQ, Claggett B, et al. Prognostic Relevance of Left Atrial Dysfunction in Heart Failure With Preserved Ejection Fraction. *Circ Heart Fail*. 2016;9(4):e002763.https://doi.org/10.1161/CIRCHEARTFAILURE.115.002763

Savarese G and Lund LH. Global Public Health Burden of Heart Failure. *Card Fail Rev*. 2017;3(1):7-11. https://doi.org/10.15420/cfr.2016:25:2

Shah AM, Clagett B, Sweitzer NK, et al. Prognostic importance of impaired systolic function in heart failure with preserved ejection fraction and the impact of spironolactone. *Circulation.* 2015;132(5):402-414. https://doi.org/10.1161/CIRCULATIONAHA.115.015884

Solomon SD, Dobson J, Pocock S, et al. Candesartan in Heart Failure: Assessment of Reduction in Mortality and Morbidity (CHARM) Investigators. *Circulation*. 2007;116(13):1482-1487. https://doi.org/10.1161/CIRCULATIONAHA.107.696906

Tan TS, Akbulut IM, Demirtola AI, et al. LA reservoir strain: a sensitive parameter for estimating LV filling pressure in patients with preserved EF. *Int J Cardiovasc Imaging.* 2021;37(9):2707–2716. https://doi.org/10.1007/s10554-021-02235-x

Tromp J, MacDonald MR, Tay WT, et al. Heart failure with preserved ejection fraction in the young. *Circulation*. 2018;138(24):2763-2773. https://doi.org/10.1161/CIRCULATIONAHA.118.034720

van der Meer P, Gaggin HK, Dec GW. ACC/AHA Versus ESC Guidelines on Heart Failure: JACC Guideline Comparison. *J Am Coll Cardiol.* 2019;73(21):2756-2768. https://doi.org/10.1016/j.jacc.2019.03.478.

van Heerebeek L and Paulus WJ. Understanding heart failure with preserved ejection fraction: where are we today? *Neth Heart J*. 2016;24(4):227-236. https://doi.org/10.1007/s12471-016-0810-1

Final

Approved on 09 Apr 2024 GMT
Downloaded by Flora Sam on 09 Apr 2024

Voors AA, Dahlke M, Meyer S, et al. Renal hemodynamic effects of serelaxin in patients with chronic heart failure: a randomized, placebo-controlled study. *Circ Heart Fail*. 2014;7(6):994-1002. https://doi/org/10.1161/CIRCHEARTFAILURE.114.001536

Wakami K, Ohte N, Asada K, et al. Correlation between left ventricular end-diastolic pressure and peak left atrial wall strain during left ventricular systole. *J Am Soc Echocardiogr*. 2009;22(7):847-851.https://doi/org/10.1016/j.echo.2009.04.026

Yancy CW, Jessup M, Bozkurt B, et al. 2013 ACCF/AHA guideline for the management of heart failure: executive summary: a report of the American College of Cardiology Foundation/American Heart Association Task Force on practice guidelines. 2013;128(16):1810-1852. https://doi.org/10.1161/CIR.0b013e31829e8807

Zile MR and Brutsaert DL. New concepts in diastolic dysfunction and diastolic heart failure: part I: diagnosis, prognosis, and measurements of diastolic function. *Circulation*. 2002;105(11):1387-1393. https://doi.org/10.1161/hc1102.105289

Final

Approved on 09 Apr 2024 GMT
Downloaded by Flora Sam on 09 Apr 2024

Signature Page for VV-CLIN-119494 v2.0

Approval	Axel Haupt<br>Medical Director<br>09-Apr-2024 12:00:50 GMT+0000

Approval	Yong Lin<br>Statistician<br>09-Apr-2024 12:55:08 GMT+0000

Signature Page for VV-CLIN-119494 v2.0

Final

Approved on 09_Apr 2024 GMT
Downloaded by Flora Sam on 09 Apr 2024
