# Supplementary material for: Effects of volenrelaxin in worsening heart failure with preserved ejection fraction: a phase 2 randomized trial
Source: Nat Med. 2025 Aug 31;31(11):3853–61. doi: 10.1038/s41591-025-03939-6 (PMC12618225; doi:10.1038/s41591-025-03939-6)
Supplement: Supplementary file 2 — Reporting Summary [file 41591_2025_3939_MOESM2_ESM.pdf]

nature portfolio

Corresponding author(s): Barry A Borlaug

Last updated by author(s): 07/08/2025

Reporting Summary

Nature Portfolio wishes to improve the reproducibility of the work that we publish. This form provides structure for consistency and transparency in reporting. For further information on Nature Portfolio policies, see our Editorial Policies and the Editorial Policy Checklist.

Statistics

For all statistical analyses, confirm that the following items are present in the figure legend, table legend, main text, or Methods section.

n/a	Confirmed	
☐	☒	The exact sample size (*n*) for each experimental group/condition, given as a discrete number and unit of measurement
☐	☒	A statement on whether measurements were taken from distinct samples or whether the same sample was measured repeatedly
☐	☒	The statistical test(s) used AND whether they are one- or two-sided<br>*Only common tests should be described solely by name; describe more complex techniques in the Methods section.*
☐	☒	A description of all covariates tested
☐	☒	A description of any assumptions or corrections, such as tests of normality and adjustment for multiple comparisons
☐	☒	A full description of the statistical parameters including central tendency (e.g. means) or other basic estimates (e.g. regression coefficient) AND variation (e.g. standard deviation) or associated estimates of uncertainty (e.g. confidence intervals)
☐	☒	For null hypothesis testing, the test statistic (e.g. *F*, *t*, *r*) with confidence intervals, effect sizes, degrees of freedom and *P* value noted<br>*Give P values as exact values whenever suitable.*
☒	☐	For Bayesian analysis, information on the choice of priors and Markov chain Monte Carlo settings
☒	☐	For hierarchical and complex designs, identification of the appropriate level for tests and full reporting of outcomes
☐	☒	Estimates of effect sizes (e.g. Cohen's *d*, Pearson's *r*), indicating how they were calculated

Our web collection on statistics for biologists contains articles on many of the points above.

Software and code

Policy information about availability of computer code

Data collection	No software was used for data collection.
Data analysis	No custom code used for analysis. All analyses were performed in SAS.

For manuscripts utilizing custom algorithms or software that are central to the research but not yet described in published literature, software must be made available to editors and reviewers. We strongly encourage code deposition in a community repository (e.g. GitHub). See the Nature Portfolio guidelines for submitting code & software for further information.

Data

Policy information about availability of data

All manuscripts must include a data availability statement. This statement should provide the following information, where applicable:

- Accession codes, unique identifiers, or web links for publicly available datasets
- A description of any restrictions on data availability
- For clinical datasets or third party data, please ensure that the statement adheres to our policy

Eli Lilly and Company provides access to all individual participant data collected during the trial, after anonymization, except for pharmacokinetic or genetic data. Data are available to request 6months after the indication studied has been approved in the USA and European Union and after primary publication acceptance, whichever is later. No expiration date of data requests is currently set once data have been made available. Access is provided after a proposal has been approved by an independent review committee identified for this purpose and after receipt of a signed data-sharing agreement. Data and documents, including the study

protocol, statistical analysis plan, clinical study report and blank or annotated case report forms, will be provided in a secure data-sharing environment. For details on submitting a request, see the instructions provided at www.vivli.org.

Research involving human participants, their data, or biological material

Policy information about studies with human participants or human data. See also policy information about sex, gender (identity/presentation), and sexual orientation and race, ethnicity and racism.

Reporting on sex and gender	The terms of sex and gender are used appropriately throughout the manuscript.
Reporting on race, ethnicity, or other socially relevant groupings	Data on race and ethnicity are provided.
Population characteristics	Detailed baseline characteristics are presented in Table 1.
Recruitment	Patients with HFpEF were recruited for cardiology and heart failure clinics an participating enrolling centers. There is no identifiable self-selection or other bias, as all patient potentially meeting the eligibility criteria were evaluated for potential enrollment.
Ethics oversight	Individual site IRBs across the 64 centers.

Note that full information on the approval of the study protocol must also be provided in the manuscript.

Field-specific reporting

Please select the one below that is the best fit for your research. If you are not sure, read the appropriate sections before making your selection.

☒ Life sciences ☐ Behavioural & social sciences ☐ Ecological, evolutionary & environmental sciences

For a reference copy of the document with all sections, see nature.com/documents/nr-reporting-summary-flat.pdf

Life sciences study design

All studies must disclose on these points even when the disclosure is negative.

Sample size	All available patients over the study period were included, and the trial was powered to detect an effect on the primary outcome of left atrial reservoir strain.
Data exclusions	Data collected after permanent discontinuation of study drug was excluded. The sponsor identified serious breach of protocol from one site in November 2023, in which all enrolled subjects were deemed not to meet a key inclusion criterion. Participants from that site (n=26) were excluded from efficacy analysis.
Replication	Replicate measurements were not obtained for clinical or laboratory data. However there was replicate confirmation for statistical analysis. Each statistical analysis was conducted independently by two analysts: a primary analyst and a validation analyst. The validation analysts independently verified the results, and all analyses yielded matching outcomes between the two analysts.
Randomization	This was a randomized trial of volenrelaxin (dosages of 25, 50, and 100 mg) vs placebo, originally allocated 1:1:1:1, then changed to 1:2:2:2 to ensure greater representation from the volenrelaxin 50 mg and 100 mg groups based upon the pharmacokinetic profile and pharmacodynamic effects on renal plasma flow and safety from study in healthy volunteers.
Blinding	This was a double blind trial.

Reporting for specific materials, systems and methods

We require information from authors about some types of materials, experimental systems and methods used in many studies. Here, indicate whether each material, system or method listed is relevant to your study. If you are not sure if a list item applies to your research, read the appropriate section before selecting a response.

Materials & experimental systems

n/a	Involved in the study
☒	☐ Antibodies
☒	☐ Eukaryotic cell lines
☒	☐ Palaeontology and archaeology
☒	☐ Animals and other organisms
☐	☒ Clinical data
☒	☐ Dual use research of concern
☒	☐ Plants

Methods

n/a	Involved in the study
☒	☐ ChIP-seq
☒	☐ Flow cytometry
☒	☐ MRI-based neuroimaging

Clinical data

Policy information about clinical studies

All manuscripts should comply with the ICMJE guidelines for publication of clinical research and a completed CONSORT checklist must be included with all submissions.

Clinical trial registration	NCT05592275
Study protocol	Included as supplemental material, along with the statistical analysis plan.
Data collection	Data were collected in individual case report forms in a clinical or hospital setting. Between February 3, 2023, and January 22, 2025, 503 patients were screened and 332 patients enrolled and randomly assigned to volenrelaxin 25 mg (n=79), 50 mg (n=83), 100 mg (n=81), or placebo (n=89) at 64 centers in 10 countries.
Outcomes	The primary endpoint of this mechanistic trial was LA reservoir strain, measured by speckle-tracking echocardiography. Secondary endpoints included measures of hemodynamic congestion, specifically changes in NT-proBNP and LA volumes, and measures of kidney function, specifically changes in eGFR, serum creatinine, and cystatin-C, and safety endpoints, including serious adverse events, and adjudicated heart failure events.<br><br>Multiple additional mechanistic measures of hemodynamic congestion were evaluated by echocardiography, including left ventricular (LV) end diastolic volume, LV global longitudinal strain, and LA filling pressure estimated by the E/e' ratio. Cardiac output was measured by echocardiography. New York Heart Association (NYHA) class was evaluated as a measure of physician-rated HF severity. Patient reported symptom severity and health impairment was quantified by the Kansas City Cardiomyopathy Questionnaire (KCCQ) total symptom score (TSS), clinical summary score (CSS) and overall summary score (OSS).

Plants

Seed stocks	NA
Novel plant genotypes	NA
Authentication	NA
